# Plasma‐Assisted Immobilization of a Phosphonium Salt and Its Use as a Catalyst in the Valorization of CO_2_

**DOI:** 10.1002/cssc.201903384

**Published:** 2020-03-17

**Authors:** Yuya Hu, Sandra Peglow, Lars Longwitz, Marcus Frank, Jan Dirk Epping, Volker Brüser, Thomas Werner

**Affiliations:** ^1^ Leibniz-Institute for Catalysis at the University of Rostock Albert-Einstein-Strasse 29a 18059 Rostock Germany; ^2^ Leibniz-Institute for Plasma Science and Technology (INP) Felix-Hausdorff-Strasse 2 17489 Greifswald Germany; ^3^ Medical Biology and Electron Microscopy Center University Medicine Rostock Stremelstrasse 14 18057 Rostock Germany; ^4^ Department Life, Light & Matter University of Rostock Albert-Einstein-Strasse 25 18059 Rostock Germany; ^5^ Institute of Chemistry Technical University of Berlin Strasse des 17 Juni 135 10623 Berlin Germany

**Keywords:** carbon dioxide fixation, cyclic carbonates, immobilization, organocatalysis, plasma chemistry

## Abstract

The first plasma‐assisted immobilization of an organocatalyst, namely a bifunctional phosphonium salt in an amorphous hydrogenated carbon coating, is reported. This method makes the requirement for prefunctionalized supports redundant. The immobilized catalyst was characterized by solid‐state ^13^C and ^31^P NMR spectroscopy, SEM, and energy‐dispersive X‐ray spectroscopy. The immobilized catalyst (1 mol %) was employed in the synthesis of cyclic carbonates from epoxides and CO_2_. Notably, the efficiency of the plasma‐treated catalyst on SiO_2_ was higher than those of the SiO_2_ support impregnated with the catalyst and even the homogeneous counterpart. After optimization of the reaction conditions, 13 terminal and four internal epoxides were converted with CO_2_ to the respective cyclic carbonates in yields of up to 99 %. Furthermore, the possibility to recycle the immobilized catalyst was evaluated. Even though the catalyst could be reused, the yields gradually decreased from the third run. However, this is the first example of the recycling of a plasma‐immobilized catalyst, which opens new possibilities in the recovery and reuse of catalysts.

## Graphical abstract

**Plasma will fix it**: Plasma polymerization is used to immobilize a phosphonium salt organocatalyst (P_cat_) in an amorphous hydrogenated carbon coating on unfunctionalized metal oxide supports. The resulting catalysts are employed in the synthesis of cyclic carbonates from epoxides and CO_2_ in up to 99 % yield under mild conditions. Furthermore, the unprecedented recycling of a catalyst immobilized by a plasma technique is reported.

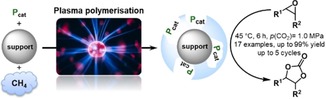

## Introduction

A crucial point in the development of sustainable catalytic processes is the separation and recycling of the catalysts.^1^ In contrast to many other separation techniques,^2^ immobilization of catalysts allows facile separation from the product without tedious purification and isolation steps as well as easy recovery and reuse of the catalyst.^3^ Numerous transformations can be catalyzed by organocatalysts, which are typically readily available and nontoxic.^4^ A significant benefit of organocatalysts is the carbon‐based scaffold, which allows facile structural modification, catalyst tuning, and catalyst immobilization.^5^

Amorphous hydrogenated carbon (a‐C:H) thin films generated with plasma techniques are promising materials owing to their chemical inertness and interesting physical properties, such as high density, thermal stability, low friction, high wear resistance, and hardness.^6^ These films are applied as protective coatings for optical windows,^7^ antireflective coatings for crystalline silicon solar cells,^8^ biomedical applications,^9^ and wear‐resistant coatings for tools.^10^ Owing to their unique properties, a‐C:H thin films are highly attractive materials for the immobilization of catalysts. An additional advantage in the use of plasma‐generated a‐C:H films is the direct attachment of the polymeric film to a desired surface without any pretreatment. Compared to other coating procedures, it reduces preparative steps and allows, in principle, the direct incorporation of a functionalized catalyst.

So far, there are only a limited number of reports regarding the immobilization of catalysts by plasma techniques. For example, Kruth et al. encapsulated Ru dyes^11^ and Ir dyes^12^ with plasma polyallylamine (PPAAm) on TiO_2_. The prepared stable TiO_2_/N_3_ (Ru dye complex)/PPAAm catalyst assemblies and encapsulated Ru sensitizer at the TiO_2_ surface showed improved catalytic performance in visible‐light‐driven hydrogen evolution. Additionally, significant enhancement of photoefficiency was observed with the PPAAm‐encapsulated Ir dye/titania catalyst assemblies. There are also some examples concerning plasma immobilization techniques in biology, for instance, the entrapment of enzymes. In this respect, Belhacene et al.^13^ and Elagli et al.^14^ reported the polymerization of tetramethyldisiloxane to immobilize β‐galactosidase by plasma‐enhanced chemical vapor deposition. Furthermore, Heyse et al.^15^ described the simultaneous injection of an enzyme solution and acetylene or pyrrole into an atmospheric plasma to immobilize enzymes while preserving their bioactivity.

**Figure 1:**
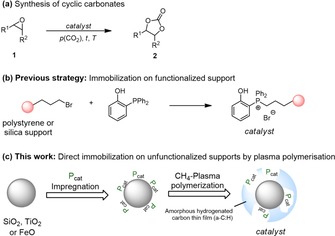
a) Synthesis of cyclic carbonates **2** from CO_2_ and epoxides **1**. b) Previous strategy for the immobilization of bifunctional phosphonium salts using functionalized supports. c) Concept for the immobilization of phosphonium salt catalysts in an a‐C:H thin film by using plasma polymerization techniques.

The atom‐economic addition of carbon dioxide to epoxides yielding cyclic carbonates is an interesting and frequently studied reaction (Figure 1 a).^16^ Lately, highly active systems based on OH‐functionalized organocatalysts were reported for the synthesis of cyclic carbonates.^17^ The superior activity of these catalysts is attributed to epoxide activation and stabilization of intermediates by hydrogen bonding.^18^ We are interested in the development of bifunctional onium salt catalysts for synthesis of cyclic carbonates as well as their recovery and reuse.17a, 17b, 19 In this respect, one strategy is the immobilization of the onium salt catalyst on organic or inorganic supports. The immobilization of monofunctional phosphonium salt catalysts was studied previously.^20^ Pioneering work on the immobilization of bifunctional structural motifs has been reported by Dai et al.^21^ and Liu et al.^22^ Recently, we reported the immobilization of a bifunctional phosphonium bromide bearing a phenol moiety utilizing functionalized polystyrene and silica supports (Figure 1 b).19b Herein, we report the use of plasma techniques for the direct immobilization of P‐based organocatalysts on unfunctionalized titanium dioxide, iron oxide, and silica (Figure 1 c). Furthermore, the efficiency and recyclability of the immobilized catalysts were studied in the synthesis of cyclic carbonates.

## Results and Discussion

**Scheme 1:**
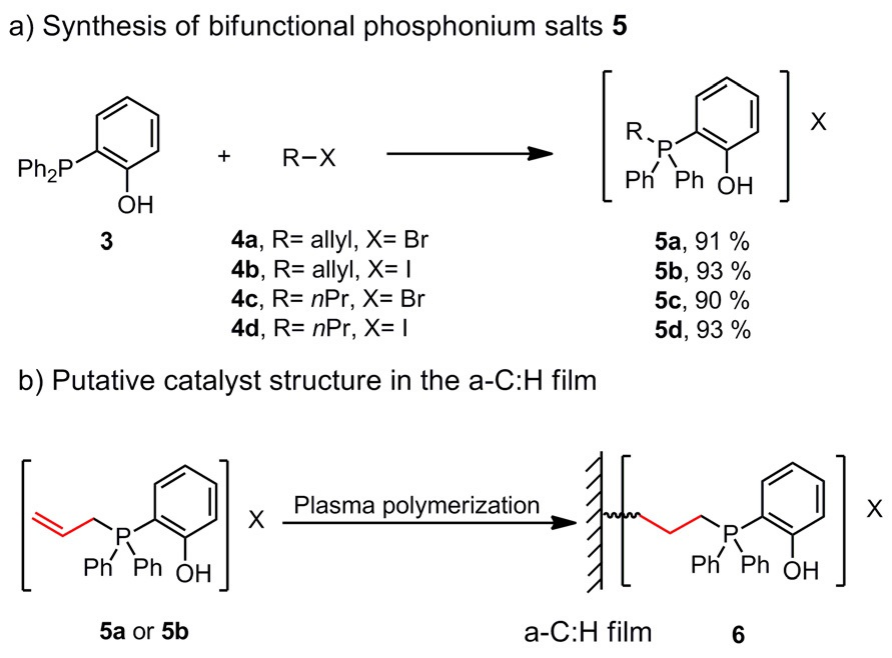
a) Synthesis of phosphonium salts **5**. b) Putative structure **6** of the immobilized phosphonium salts **5 a** and **5 b**.

Bifunctional phosphonium salts bearing a hydroxyl group in the 2‐position proved to be a superior structural motif in the cycloaddition of CO_2_ and epoxides to form cyclic carbonates.^23^ We envisioned that an allyl substituent might allow subsequent immobilization in an a‐C:H thin film generated by plasma techniques. Thus, bifunctional phosphonium salts **5 a** and **5 b** were synthesized by allylation of 2‐(diphenylphosphanyl)phenol (**3**) with allyl bromide (**4 a**) and allyl iodide (**4 b**), as shown in Scheme 1 a. The incorporation of **5 a** and **5 b** into the a‐C:H films most probably leads to a saturated linkage in the immobilized catalyst **6** (Scheme 1 b). Hence, we additionally prepared salts **5 c** and **5 d** bearing a saturated side chain for comparison of the activity.

Subsequently, we tested catalysts **5** (1 mol %) in the model reaction of 1,2‐butylene oxide (**1 a**) with CO_2_ to generate cyclic carbonate **2 a** (Table 1). At 90 °C and a CO_2_ pressure of 1.0 MPa, bromide **5 a** and iodide **5 b** showed similar activity, giving the desired carbonate **2 a** after 2 h in 68 and 67 % yield, respectively (Table 1, entries 1 and 2). Propyl‐substituted phosphonium bromide **5 c** gave **2 a** in only 40 % yield (Table 1, entry 3). Notably, iodide **5 d** gave the best result under these reaction conditions, and 1,2‐butylene carbonate (**2 a**) was obtained in 83 % yield (Table 1, entry 4). On the basis of these results, phosphonium salt **5 b** was chosen for immobilization in a‐C:H films on TiO_2_, FeO, and SiO_2_.

**Table 1:** Comparison of phosphonium salts **5** as catalysts in the synthesis of carbonate **2 a**. 
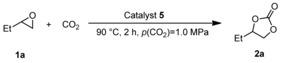

Entry	Catalyst	Loading [mol %]	Yield of **2 a** ^[a]^ [%]
1	**5 a**	1	68
2	**5 b**	1	67
3	**5 c**	1	40
4	**5 d**	1	83

Reaction conditions: epoxide **1 a** (13.9 mmol, 1.0 equiv.), catalyst **5** (1 mol %), 90 °C, 2 h, *p*(CO_2_)=1.0 MPa, solvent‐free. [a] Yields determined by ^1^H NMR spectroscopy with mesitylene as internal standard.

Initially, the supports were tested in the model reaction and proved not to facilitate the reaction of **1 a** with CO_2_ (Table 2, entries 1–3). Subsequently, these supports were treated with low‐pressure plasma to generate an a‐C:H coating.^[24]^ Also, in the presence of the plasma‐treated supports, the formation of **2 a** was not observed (Table 2, entries 4–6). The supports were impregnated with catalyst **5 b** and tested in the model reaction (Table 2, entries 7–9). The catalyst retained its catalytic activity, and all three catalysts **5 b**@TiO_2_, **5 b**@FeO, and **5 b**@SiO_2_ gave 1,2‐butylene carbonate (**2 a**) in high yields of 87, 78, and 88 %, respectively (Table 2, entries 7–9). Subsequently, the impregnated supports **5 b**@TiO_2_, **5 b**@FeO, and **5 b**@SiO_2_ were treated with a low‐pressure plasma. The obtained catalysts **5 bb**℗TiO_2_, **5 bb**℗FeO, and **5 bb**℗SiO_2_ were tested in the model reaction (Table 2, entries 10–12). Notably, with 1 mol % catalyst loading, TiO_2_‐ and SiO_2_‐supported catalysts converted 1,2‐butylene oxide (**1 a**) to 1,2‐butylene carbonate (**2 a**) in 93 and 99 % yield (Table 2, entries 10 and 12), whereas with the FeO‐supported catalyst a moderate yield of 72 % was obtained (Table 2, entry 11). These yields are comparable to those obtained with the impregnated supports (Table 2, entries 7–9 versus 10–12).

**Table 2:** Screening of supports and immobilized catalysts. 
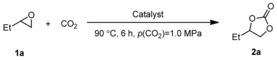

Entry	Support	**5 b** [mol %]	Cat.	*t* ^[a]^ [min]	Yield of **2 a** ^[b]^ [%]
1	TiO_2_	–		–	0
2	FeO	–		–	0
3	SiO_2_	–		–	0
4	TiO_2_	–		25	0
5	FeO	–		25	0
6	SiO_2_	–		25	0
7	TiO_2_	1	**5 b**@TiO_2_	–	87
8	FeO	1	**5 b**@FeO	–	78
9	SiO_2_	1	**5 b**@SiO_2_	–	88 (65)^[c]^
10	TiO_2_	1	**5 bb**℗TiO_2_	25	93
11	FeO	1	**5 bb**℗FeO	25	72
12	SiO_2_	1	**5 bb**℗SiO_2_	25	99 (77)^[c]^

Reaction conditions: epoxide **1 a** (13.9 mmol, 1.0 equiv.), support or catalyst (500 mg), 90 °C, 6 h, *p*(CO_2_)=1.0 MPa, solvent‐free. [a] Plasma‐treating time. [b] Yield determined by ^1^H NMR spectroscopy with mesitylene as internal standard. [c] 2 h reaction time.

**Figure 2:**
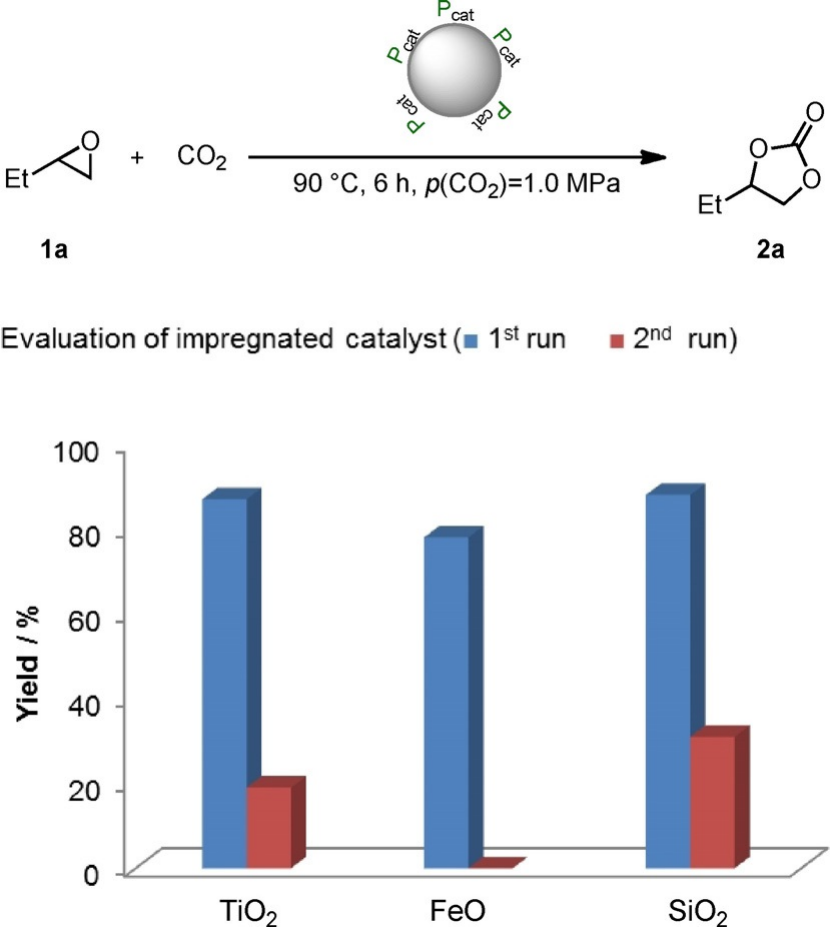
Recyclability evaluation of impregnated catalysts **5 b**@TiO_2_, **5 b**@FeO, and **5 b**@SiO_2_. Reaction conditions: epoxide **1 a** (13.9 mmol, 1.0 equiv.), immobilized catalyst (500 mg, 1 mol % catalyst loading in respect to **1 a**), 90 °C, 6 h, *p*(CO_2_)=1.0 MPa, solvent‐free. For the first runs yields of isolated products are given. For the second runs the yield was determined by ^1^H NMR spectroscopy with mesitylene as internal standard.

The nominal layer thickness of the a‐C:H coating is related to the plasma‐treating time. Longer treating times result in a thicker film and better coverage of the particles. This may lead to stronger catalyst binding to the surface, which reduces leaching of the catalyst and enhances its recyclability. The nominal layer thickness was determined by profilometry of an a‐C:H coating deposited on a planar glass plate.^[24]^ This is only an approximation for films on particles because the planar glass plate is homogeneously coated, whereas the deposition on particles is nonuniform and partial. Profilometric measurements of the nominal layer thickness of a‐C:H films obtained after 6.5, 25, and 39 min of plasma treatment gave layer thicknesses of 53.3, 136.8, and 190 nm, respectively. We studied the impact of different plasma‐treating times (6.5, 25, and 39 min) on the catalytic activity of **5 b** on TiO_2_, FeO, and SiO_2_ and the effect of the catalyst recyclability in our model reaction. To reveal the effect of the plasma treatment, the recycling of the non‐plasma‐treated impregnated catalysts **5 b**@TiO_2_, **5 b**@FeO, and **5 b**@SiO_2_ was initially investigated (Figure 2). In the model reaction all three catalysts gave good yields of up to 88 % after 6 h at 90 °C and 1.0 MPa CO_2_ pressure in the first run. The product was obtained after simple filtration, and the recovered catalyst was reused in a second run under the same reaction conditions. Notably, the yields dropped significantly. The best yield achieved in the second run was only 31 % with **5 b**@SiO_2_. We assumed that the low yields can be explained by leaching of catalyst **5 b** into the liquid phase. This is easily possible because the catalyst is not covalently bonded to the supports. The ^31^P NMR spectrum of the product mixture showed a signal at *δ*=20.2 ppm, which was assigned to homogeneous catalyst **5 b**. This consequently confirms the proposed leaching.

**Figure 3:**
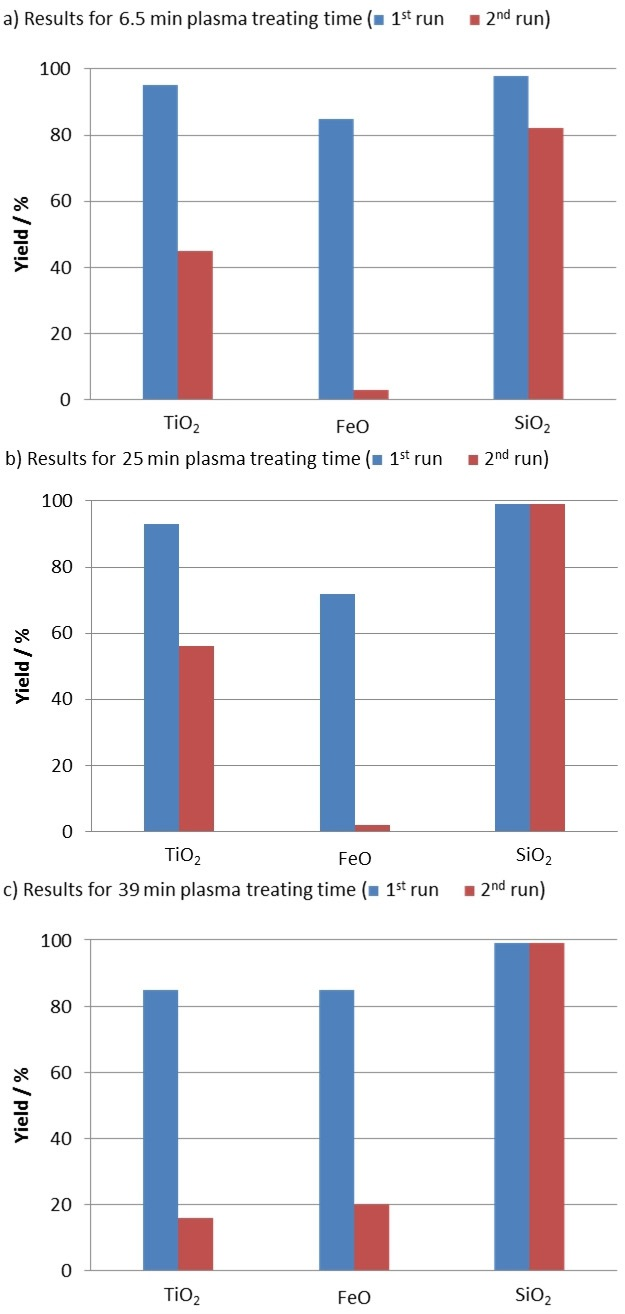
Recyclability evaluation of catalyst **5 b** on TiO_2_, FeO, and SiO_2_ with different plasma‐treating times: a) 6.5 min, b) 25 min, c) 39 min plasma‐treating time. Reaction conditions: epoxide **1 a** (13.9 mmol, 1.0 equiv.), immobilized catalyst (500 mg, 1 mol % catalyst loading with respect to **1 a**), 90 °C, 6 h, *p*(CO_2_)=1.0 MPa, solvent‐free. For the first runs yields of isolated products are given. For the second runs the yield was determined by ^1^H NMR spectroscopy with mesitylene as internal standard.

We studied immobilized catalyst **5 b** on different supports (TiO_2_, FeO, and SiO_2_) after 6.5 min plasma‐treating time under the same conditions. Catalysts **5 ba**℗TiO_2_, **5 ba**℗FeO, and **5 ba**℗SiO_2_ gave the desired carbonate **2 a** in good to excellent yields up to 98 % (Figure 3 a). Even though with catalysts **5 ba**℗TiO_2_ and **5 ba**℗FeO the yields dropped significantly in the second run, in the presence of **5 ba**℗SiO_2_ carbonate **2 a** was obtained in greater than 80 % yield. These results might be explained by insufficient immobilization owing to the short plasma‐treating time. Nevertheless, compared with the impregnated catalysts, the plasma treatment led to a significant improvement of the yield (Figure 2 vs. Figure 3 a).

Hence, the same set of experiments was repeated with catalysts **5 bb**℗TiO_2_, **5 bb**℗FeO, and **5 bb**℗SiO_2_ obtained after 25 min plasma‐treating time (Figure 3 b). In the first run, all three catalysts gave results comparable to those of **5 ba**℗TiO_2,_ **5 ba**℗FeO, and **5 ba**℗SiO_2_ (Figure 3 a vs. b, 1^st^ run). The yields for **2 a** were significantly increased in the case of the TiO_2_‐ and SiO_2_‐supported catalysts (**5 bb**℗TiO_2_ and **5 bb**℗SiO_2_) in the second run (Figure 3 a vs. b, 2^nd^ run). This indicates that prolonged plasma‐treating time leads to improved catalyst binding to the a‐C:H coatings. Finally, the plasma‐treating time was extended to 39 min, and the prepared catalysts were tested under the standard conditions (Figure 3 c). In the case of **5 bc**℗TiO_2_ the yields dropped in the first and second runs compared to the results for shorter treating times (Figure 3 a and b). In contrast, **5 bc**℗FeO showed increased yields compared with the previous experiments. Again, the best result was obtained with **5 bc**℗SiO_**2**_, which gave a 99 % yield of **2 a** in the first and second runs.

As observed for **5 bc**℗TiO_2_, longer plasma‐treating times may lead to better recyclability, most probably owing to improved immobilization (Figure 3 a and b). However, if the plasma‐treating time is too long, for example, 39 min for **5 bc**℗TiO_2_, this may lead to partial coverage of the catalyst and thus lower yields (Figure 3 c vs. a and b). In the case of **5 bb**℗FeO the enhanced yield and recyclability for a plasma‐treating time of 39 min indicated better immobilization of **5 b** on the support. This suggests that not only the plasma‐treating time but also the nature of the support material is of crucial importance for the efficiency and recyclability of the catalyst.

**Figure 4:**
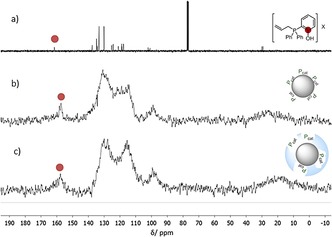
a) ^13^C NMR spectrum of homogeneous catalyst **5 b** in CDCl_3_. b) Solid‐state ^13^C NMR spectrum of impregnated catalyst **5 b**@SiO_2_. c) Solid‐state ^13^C NMR spectrum of plasma‐immobilized catalyst **5 bb**℗SiO_2_.

On the basis of these results, **5 bb**℗SiO_2_ was identified to be the most promising catalyst. Thus, **5 bb**℗SiO_2_ was characterized with various analytical methods and compared with homogeneous catalyst **5 b** and **5 b**‐impregnated SiO_2_ (**5 b**@SiO_2_). As expected, the elemental analysis of both impregnated catalyst **5 b**@SiO_2_ and plasma‐treated catalyst **5 bb**℗SiO_2_ showed the presence of phosphorus and iodine. The solid‐state ^31^P NMR spectrum of plasma‐treated catalyst **5 bb**℗SiO_2_ showed a broad signal at *δ*=16.9 ppm, which is in a similar range to that in the ^31^P NMR spectrum of homogeneous catalyst **5 b** (*δ*=20.2 ppm), indicating the presence of a phosphonium motif. The solid‐state ^13^C NMR spectra of impregnated **5 b**@SiO_2_ and plasma‐treated **5 bb**℗SiO_2_ showed the expected signals compared with the ^13^C NMR spectrum of homogeneous catalyst **5 b** (Figure 4 a–c). Notably, the characteristic signal for the phenolic carbon atom at *δ*=161 ppm for **5 b** can clearly be identified in the solid‐state spectrum of **5 b**@SiO_2_ and **5 bb**℗SiO_2_. This is of particular importance because it indicates that the bifunctional nature of the immobilized catalyst stays intact, which is crucial for its superior catalytic activity.

**Figure 5:**
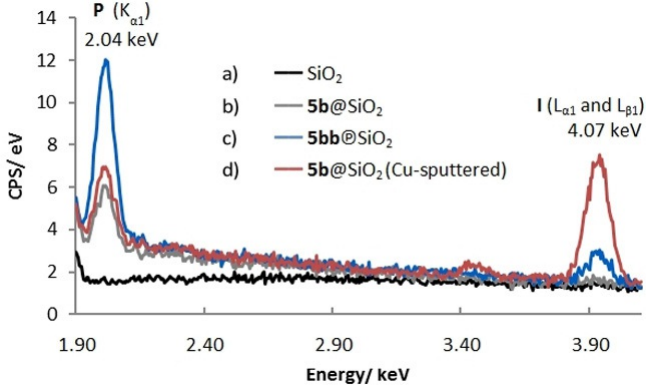
Sections of EDX spectra between 1.90 and 4.10 keV for a) the SiO_2_ support (black, carbon‐coated), b) the impregnated catalyst **5 b**@SiO_2_ (gray, carbon‐coated), c) the plasma‐treated catalyst **5 bb**℗SiO_2_ (blue, carbon‐coated), and d) the impregnated catalyst **5 b**@SiO_2_ (red, Cu‐sputtered).^[24]^

Energy‐dispersive X‐ray (EDX) spectroscopy was performed on impregnated **5 b**@SiO_2_, plasma‐treated **5 bb**℗SiO_2_, and the SiO_2_ support.^[24]^ The EDX spectrum of the SiO_2_ support showed no signal in the range between 1.90 and 4.10 keV (Figure 5 a). In contrast, the impregnated and plasma‐treated materials showed signals at 2.04 keV (P K_α_), which indicate the presence of phosphorus (Figure 5 b and c). Notably, **5 b**@SiO_2_ does not show an iodine signal (Figure 5 b), whereas **5 bb**℗SiO_2_ has a low‐intensity signal at 4.07 keV (I L_α1_ and L_β1_), which is characteristic for iodine (Figure 5 c). The absence of the signal for I L_α1_ and L_β1_ in Figure 5 b and the low intensity of the signal in Figure 5 c can be explained by the difficulty of detecting surface‐associated iodine, which results from the high energy required for excitation of the iodine L transitions. This can be overcome by changing the sample pretreatment; for example, the EDX spectrum of copper‐sputtered **5 b**@SiO_2_ clearly showed the presence of iodide (Figure 5 d). The copper layer (*z=*29, 10 nm) altered the penetration and spread of the electron beam in the sample surface compared with the rather electron‐transparent carbon coating (*z=*6, 10–15 nm). Notably, comparable peaks for phosphorus are obtained under both pretreatment conditions (Figure 5 b and d).

**Figure 6:**
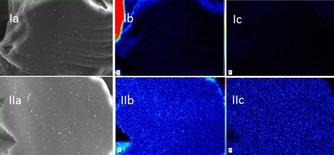
SEM images of the silica support (Ia) and catalyst **5 bb**℗SiO_2_ (IIa). EDX mapping with color‐coded intensity range of carbon (Ib) and phosphorus (Ic) for the silica support. EDX mapping with color‐coded intensity range of carbon (IIb) and phosphorus (IIc) for the immobilized catalyst **5 bb**℗SiO_2_.^[24]^

Moreover, we studied **5 bb**℗SiO_2_ by SEM and EDX mapping in comparison to the neat support (Figure 6). The SEM images of the silica support and **5 bb**℗SiO_2_ are shown in Figure 6 Ia and IIa. The carbon EDX mapping of these particles shows clearly an increase in carbon surface coating owing to plasma treatment (Figure 6 Ib vs. IIb). The mapping for phosphorus indicates that the catalyst is evenly distributed over the support, and the absence of phosphorous on the neat support (Figure 6 Ic and IIc).

Subsequently we studied the performance of catalyst **5 bb**℗SiO_2_ under different reaction conditions (Table 3). Under the conditions of the catalyst screening, the desired product **2 a** was obtained in 99 % yield (Table 3, entry 1). Decreasing the reaction time to 3 h gave **2 a** in 99 % yield of isolated product (Table 3, entry 2), and even after 1 h a yield of 57 % was obtained (Table 3, entry 3). The influence of the CO_2_ pressure was also investigated. Decreasing the CO_2_ pressure to 0.5 MPa led to a lower yield of 88 % compared to the standard conditions (Table 3, entry 1 vs. 4). Next, the reaction temperature was decreased to 45 °C. Even at 45 °C the immobilized catalyst **5 bb**℗SiO_2_ led to full conversion and 99 % yield after 6 h (Table 3, entry 5). Notably, a 21 % yield of **2 a** was still obtained after 3 h at this temperature (Table 3, entry 6).

**Table 3:** Optimization of catalytic reaction conditions for the conversion of 1,2‐butylene oxide (**1 a**). 
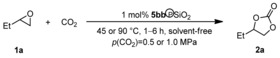

Entry	*T* [°C]	*p* [MPa]	*t* [h]	Yield **2 a** ^[a]^ [%]
1	90	1.0	6	99
2	90	1.0	3	99
3	90	1.0	1	57
4	90	0.5	6	88
5	45	1.0	6	99
6	45	1.0	3	21

Reaction conditions: epoxide **1 a** (13.9 mmol, 1.0 equiv.), immobilized catalyst **5 bb**℗SiO_2_ (500 mg, 1 mol % catalyst loading with respect to **1 a**), *T*, *t*, *p*, solvent‐free. [a] Yields of isolated products are given.

**Scheme 2:**
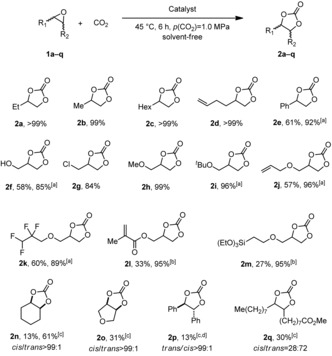
Evaluation of the substrate scope with catalyst **5 bb**℗SiO_2_. Reaction conditions: epoxide **1** (13.9 mmol, 1.0 equiv.), **5 bb**℗SiO_2_ (500 mg, 1 mol % catalyst loading with respect to **1**), 45 °C, 6 h, *p*(CO_2_)=1.0 MPa, solvent‐free. Yields of isolated products are given. [a] 24 h. [b] 90 °C. [c] 90 °C, 24 h. [d] 1.0 mL *n*‐BuOH was used as the solvent.

On the basis of these results we determined reaction conditions suitable for the evaluation of the substrate scope (1 mol % catalyst **5 bb**℗SiO_2_, 45 °C, 6 h, *p*(CO_2_)=1.0 MPa, solvent‐free). As shown in Scheme 2, terminal aliphatic epoxides **1 a**–**d** were converted to the respective carbonates **2 a**–**d** in yields of up to >99 % under these conditions. In contrast, styrene oxide (**1 e**) showed only moderate conversion, and **2 e** was obtained in a yield of 61 %. However, with a prolonged reaction time of 24 h, full conversion was achieved, and the desired product was isolated in 92 % yield. In this reaction acetophenone from a Meinwald rearrangement was observed as a byproduct.^25^

Glycerol has become widely available because it is the major byproduct in the manufacturing of biodiesel.^[26]^ “Biodiesel” is a popular term for the fatty acid methyl esters formed by transesterification of vegetable oils with methanol.^[27]^ It has been shown that the use of glycerol as the feedstock for the synthesis of carbonates can lead to a significant reduction in the carbon footprint of their production compared with the use of fossil resources.^28^ Glycidol (**1 f**), epichlorohydrin (**1 g**), and their derivatives **1 h**–**1 m** can be obtained from glycerol as renewable feedstock.^[29]^ The respective carbonates often show unique properties and are used as synthetic building blocks, monomers, and solvents.^[30]^ Hence, we were particularly interested in the preparation of carbonates **2 f**–**2 m**. Despite notable progress that was recently reported in the reaction of glycidol (**1 f**) with CO_2_ to form carbonate **2 f**, the conversion of **1 f** is challenging.^31^ Especially the use of heterogeneous catalysts in this reaction typically requires drastic reaction conditions such as high reaction temperatures (≥110 °C) and high CO_2_ pressure (≥1 MPa).^[32]^ Under the standard reaction conditions, **2 f** was obtained in 58 % yield and in 85 % yield on extending the reaction time. In contrast, **2 g** and **2 h** were isolated in 84 and 99 % yield, respectively, after 6 h. However, to achieve full conversion of the other glycidol derivatives **1 i**–**1 m**, the reaction conditions were adjusted, and high yields of up to 96 % of the respective carbonates **2 i**–**2 m** were achieved. Of particular interest is product **2 k**, which was obtained in 89 % yield and is used as an electrolyte in lithium‐ion batteries,^[33]^ as well as glycerol carbonate methacrylate **2 l** and siloxane **2 m**, both of which were isolated in 95 % yield and are used as monomers and adhesion promotors.^[34]^

We then turned our attention to the conversion of internal epoxides with CO_2_ which is in general more challenging. Under the standard conditions, **2 n** was obtained in only 13 % yield. At a higher reaction temperature of 90 °C, carbonate **2 n** was obtained in 61 % yield after 24 h, which is a good result for an internal epoxide considering that a heterogeneous organocatalyst with low loading (1 mol %) was used. Full conversion was achieved for the reaction between 3,4‐epoxytetrahydrofuran (**1 o**) and CO_2_. However, owing to partial polymerization only 31 % of the desired product **2 o** was isolated. The conversion of *cis*‐stilbene oxide (*cis*‐**1 p**) and epoxidized methyl oleate (*cis*‐**1 q**) gave the desired cyclic carbonates in yields of 13 and 30 %, respectively. For the reaction of *cis*‐**1 p** a solvent was required because both the substrate and product are solid. With respect to the stereochemistry, in the case of *cis*‐**1 p** the only product observed was the thermodynamically more stable *trans*‐**2 p**, which indicates that in this case the reaction proceeds via a cationic intermediate and by an S_N_1‐type mechanism.^35^ Similarly, the conversion of biobased *cis*‐**1 q** led to **2 q** as a mixture of *cis*/*trans* isomers (28:72).

**Figure 7:**
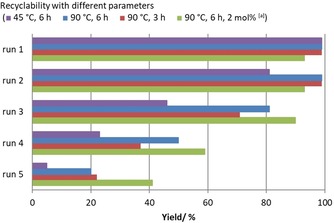
Recyclability investigation for catalyst **5 bb**℗SiO_2_ at different reaction temperatures and times. Reaction conditions: epoxide **1 a** (13.9 mmol, 1.0 equiv.), immobilized catalyst (500 mg, 1 mol % catalyst loading in respect to **1 a**), *T*, *t*, *p*(CO_2_)=1.0 MPa, solvent‐free. Yields of isolated products are given for the first run. For runs 2–5 the yields were determined by ^1^H NMR spectroscopy with mesitylene as internal standard. [a] 2 mol % catalyst loading with respect to **1 a**.

Finally, we studied the recyclability of the plasma‐treated catalyst on SiO_2_ in more detail. At first the impact of the different reaction parameters on the outcome of the model reaction over five runs with **5 bb**℗SiO_2_ as catalyst was evaluated. Under the standard conditions of the substrate screening the recycling experiments revealed that at 45 °C the yield decreased from greater than 99 % in the first run to 81 % in the second run to less than 10 % in the fifth run (Figure 7).

Improved yields were achieved at a higher reaction temperature of 90 °C. At this temperature **2 a** was obtained in greater than 99 % yield in the first and second runs. In the subsequent runs the yield gradually decreased to 20 %. We envisioned that catalyst leaching is responsible for the decreased yields and postulated that the degree of leaching might correlate to the reaction time. Thus, we reduced the reaction time to 3 h and repeated catalyst recycling (Figure 7). Even though similar results were obtained in the first and second runs, the yields in the following runs could not be improved. As expected, with a higher catalyst loading of 2 mol %, the yields of **2 a** were significantly improved in runs 3–5, though in this set of experiments the yield gradually decreased from 90 % in the third run to 41 % in the last run.

**Figure 8:**
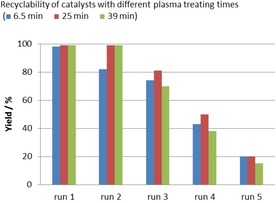
Recyclability investigation of SiO_2_‐supported catalyst **5 b** with different plasma‐treating times: **5 ba**℗SiO_2_ (6.5 min), **5 bb**℗SiO_2_ (25 min), and **5 bc**℗SiO_2_ (39 min). Reaction conditions: epoxide **1 a** (13.9 mmol, 1.0 equiv.), immobilized catalyst (500 mg, 1 mol % catalyst loading in respect to **1 a**), 90 °C, 6 h, *p*(CO_2_)=1.0 MPa, solvent‐free. Yields of isolated products are given for the first run. For runs 2–5 the yields were determined by ^1^H NMR spectroscopy with mesitylene as internal standard.

Owing to these results we were especially interested in the impact of different plasma‐treating times (6.5 min for **5 ba**℗SiO_2_, 25 min for **5 bb**℗SiO_2_, and 39 min for **5 bc**℗SiO_2_; Figure 8) on the recyclability of the catalysts. Full conversions and yields greater than 99 % were achieved in the first run for all three catalysts. The same results were achieved with catalysts **5 bb**℗SiO_2_ and **5 bc**℗SiO_2_ in the second run, whereas **5 ba**℗SiO_2_ gave a lower yield of 82 %, which might be attributable to insufficient immobilization owing to the short treatment time. In the third run the yields in the presence of all three catalysts were decreased. Catalyst **5 bb**℗SiO_2_ gave the best result, yielding **2 a** in 81 % yield, whereas **5 ba**℗SiO_2_ and **5 bc**℗SiO_2_ gave **2 a** in similar yields of 70 and 74 % respectively. This trend further continued for all three catalysts, and yields of 20 % or less were observed in the fifth run. Apparently, a plasma‐treating time of 25 min for **5 bb**℗SiO_2_ led to a good balance between binding to the a‐C:H coating (compared with **5 ba**℗SiO_2_) and its thickness, to avoid coverage of the catalytically active species (compared with **5 bc**℗SiO_2_).

**Figure 9:**
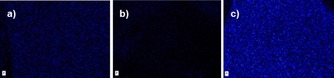
EDX mapping with color‐coded intensity range of phosphorus. a) **5 bb**℗SiO_2_ after five reaction cycles, b) neat SiO_2_ support, and c) immobilized catalyst **5 bb**℗SiO_2_.^[24]^

To get better insight into catalyst deactivation, **5 bb**℗SiO_2_ was isolated after the fifth run and analyzed by solid‐state NMR spectroscopy, SEM, EDX spectroscopy, and elemental analysis. Notably, the elemental analysis indicated that the phosphonium salt is detached from the surface of the SiO_2_ support. This is supported by the ^31^P NMR spectrum, which did not show any phosphorus signal, and the solid‐state ^13^C NMR spectrum, which did not show the expected signals from the aryl substituents at the phosphorus atom in the aromatic region. In contrast the ^31^P NMR spectra of the products obtained in the first and second runs clearly indicated leaching of the catalyst into the product. Notably, the elemental analysis of the used catalyst showed higher carbon and hydrogen contents, and the solid‐state ^13^C NMR spectrum showed several new multiplets between 0 and 80 ppm, which indicate product deposition on the catalyst surface. However, considering that the sample still showed catalytic activity, the concentration of the catalyst on the surface may be below the detection limit of these methods. In contrast, the EDX mapping showed the presence of a small amount of evenly dispersed phosphorus compared to neat support (Figure 9 a vs. b). However, the concentration of phosphorus after the fifth run was still significantly lower than that of the fresh catalyst (Figure 9 a vs. c).

## Conclusion

We designed and synthesized a functionalized phosphonium salt suitable for plasma immobilization. The obtained catalysts were tested in the synthesis of 1,2‐butylene carbonate from CO_2_ and 1,2‐butylene oxide as the model reaction. Among the three tested potential supports (TiO_2_, FeO, and SiO_2_), SiO_2_ proved to be the most suitable. In initial recycling experiments the support impregnated with the catalyst was compared with its plasma‐treated counterpart. These experiments revealed a clear advantage of the plasma treatment. Remarkably, the immobilized catalyst even showed efficiency similar to (or higher than) that of its homogeneous analogue. Furthermore, the impact of different plasma‐treating times on the efficiency and recyclability was investigated. The best catalytic material was characterized by solid‐state NMR spectroscopy, elemental analysis, SEM, and energy‐dispersive X‐ray spectroscopy. The analysis revealed the formation of an a‐C:H coating and the presence of the catalytically active species. After optimization of the reaction conditions, 13 terminal and four internal epoxides were converted with CO_2_ to the respective cyclic carbonates in yields of up to 99 %. Special attention was paid to the conversion of eight glycerol derivatives that can be obtained from glycerol, which is a byproduct of biodiesel production. Considering that a heterogeneous catalyst was used, it is noteworthy that most of the terminal substrates could be efficiently converted to the desired products under mild reaction conditions (45 °C, 6 h, *p*(CO_2_)=1.0 MPa) with a low catalyst loading of 1 mol %. Subsequently, we studied the recyclability of the catalyst for the model reaction in detail. Even though the catalyst could be used in five consecutive runs, the yields gradually decreased from the second to the fifth run. The analysis of the produced cyclic carbonate as well as the characterization of the catalyst after the fifth run revealed catalyst leaching into the product phase. The optimization of the coating process may allow the reduction of the catalyst leaching and is currently under investigation. To the best of our knowledge, this is the first example on the successful recycling of a plasmaimmobilized catalyst. This proof of concept opens the opportunity for further studies on the application of plasma polymerization techniques in catalyst recycling.

## Experimental Section

### Preparation of bifunctional catalysts 5

A mixture of phosphane **3** (1.0 equiv.) and alkyl halides **4** (5.0 equiv.) was stirred for 24 h at 23–102 °C under argon atmosphere. The crude product was washed with diethyl ether and dried under vacuum.

### Procedure for the screening of homogeneous catalyst

A 45 cm^3^ stainless‐steel autoclave was charged with catalyst **5** (1 mol %). Subsequently, 1,2‐butylene oxide (**1 a**, 1.00 g, 13.9 mmol, 1.0 equiv.) was added. The autoclave was purged with CO_2_ and heated to 90 °C for 2 h, while *p*(CO_2_, 90 °C) was kept constant at 1.0 MPa. The reactor was cooled with an ice bath below 20 °C, and CO_2_ was released slowly. The conversion of the epoxide **1 a** and yield of the carbonate **2 a** were determined by ^1^H NMR spectroscopy from the reaction mixture using mesitylene as internal standard.

### Procedure for the impregnation of different supports with catalyst 5 b

Phosphonium salt **5 b** (119 mg, 0.278 mmol), was dissolved in CH_2_Cl_2_ (125 mL). The respective support (TiO_2_, FeO, or SiO_2_, 1.00 g) was added to the solution. The suspension was shaken for 16 h at 23 °C. Subsequently all volatile substances were removed under vacuum to obtain the support impregnated with catalyst **5 b** (12 wt % on TiO_2_, FeO, or SiO_2_).

### Procedure for the plasma‐assisted immobilization of catalyst 5 b on different supports

TiO_2_, FeO, or SiO_2_ impregnated with catalyst **5 b** (2.00 g, 12 wt % **5 b**) was dispersed on a sample holder in the vacuum chamber of the plasma‐deposition device. After a pumping time of approximately 2 h, a gas mixture consisting of argon and methane in ratio 1:1 (40 sccm) was admitted. After a waiting period of 5 min the plasma power (600 W, 13.56 MHz) was switched on. The pressure of 15 Pa was controlled by pressure gauge and butterfly valve. The plasma‐treatment time was varied between 6.5, 25, and 39 min.

### Catalyst and parameter screening

A 45 cm^3^ stainless‐steel autoclave was charged with the impregnated or plasma‐treated catalyst (500 mg, 1 or 2 mol %) and 1,2‐butylene oxide (**1 a**, 1.00 g, 13.9 mmol, 1.0 equiv.). The autoclave was purged with CO_2_, and the reactor was heated to 45 or 90 °C for 3–24 h, while *p*(CO_2_, 90 °C) was kept constant at 1.0 MPa. The reactor was cooled with an ice bath to below 20 °C, and CO_2_ was released slowly. The conversion of the epoxide **1 a** and the yield of the carbonate **2 a** were determined by ^1^H NMR spectroscopy of the reaction mixture with mesitylene as internal standard.

### Protocol for the catalyst recycling experiments

A 45 cm^3^ stainless‐steel autoclave was charged with the catalyst **5 bb**℗SiO_2_ (500 mg, 1 or 2 mol % loading) and 1,2‐butylene oxide (**1 a**, 1.0 g,13.9 mmol, 1.0 equiv.). The autoclave was purged with CO_2_ and heated to 45 or 90 °C for 2 or 6 h, while *p*(CO_2_, 90 °C) was kept constant at 1.0 MPa. Subsequently the reactor was cooled to below 20 °C with an ice bath, and CO_2_ was released slowly. The reaction mixture was removed by extraction with Et_2_O (3×30 mL). All volatile substances were removed under vacuum to yield 1,2‐butylene carbonate (**2 a**). The catalyst was dried in air overnight and reused. The conversion of the epoxide **1 a** and yield of the desired carbonate were determined either for isolated product or by ^1^H NMR spectroscopy with mesitylene as internal standard.

## Conflict of interest

*The authors declare no conflict of interest*.

## Supporting information

As a service to our authors and readers, this journal provides supporting information supplied by the authors. Such materials are peer reviewed and may be re‐organized for online delivery, but are not copy‐edited or typeset. Technical support issues arising from supporting information (other than missing files) should be addressed to the authors.

Supplementary
