# Supplementary material for: Plasma‐Assisted Immobilization of a Phosphonium Salt and Its Use as a Catalyst in the Valorization of CO2
Source: ChemSusChem. 2020 Mar 17;13(7):1825–33. doi: 10.1002/cssc.201903384 (PMC7186948; doi:10.1002/cssc.201903384)
Supplement: Supplementary file 1 — Supplementary [file CSSC-13-1825-s001.pdf]

CHEMISTRY & SUSTAINABILITY
CHEMSUSCHEM
ENERGY & MATERIALS

Supporting Information

Plasma-Assisted Immobilization of a Phosphonium Salt and Its Use as a Catalyst in the Valorization of CO_2

Yuya Hu,[a] Sandra Peglow,[b] Lars Longwitz,[a] Marcus Frank,[c, d] Jan Dirk Epping,[e] Volker Brüser,[b] and Thomas Werner*[a]

cssc_201903384_sm_miscellaneous_information.pdf

Content

1. General considerations S1

2. Preparation and characterization of phosphonium salt catalysts S2

2.1 Preparation of the homogeneous phosphonium salt catalysts S2

2.2 Procedure for the screening of homogeneous catalyst (Table 1) S4

2.3 Procedure for the impregnation of different supports with 5b S4

2.4 Amorphous hydrogenated carbon (a-C:H) thin films S4

2.5 Procedure for the plasma assisted immobilization of catalyst 5b on different supports S5

2.6 NMR spectra for the preparation phosphonium salt catalysts S7

3. Experimental procedure for catalyst, parameter, substrate screening and catalyst recycling S23

3.1 Catalyst and parameter screening (Table 2 and Table 3) S23

3.2 Protocol for the catalyst recycling experiments S23

3.3 Solid state ^{13}C NMR and ^{31}P NMR spectra of recycled catalyst after 5 runs S27

4. SEM images and EDX mappings S29

5. Synthesis of cyclic carbonates 2 S33

6. NMR spectra of the synthesized carbonates S38

7. References S55

1. General considerations

All chemicals were purchased from commercial sources in purities of ≥95% and used without further purification. The TiO_2 nanopowder (21 nm particle size, >99.5%) and FeO (10 mesh, 99.8% trace metals basis) were purchased from *Sigma-Aldrich Chemie GmbH*, SiO_2 (60, 230–400 mesh) was purchased from *Roth GmbH*. Deuterated solvents were ordered from *Deutero GmbH* and stored over molecular sieves (3 Å). NMR spectra were received using *Bruker* 300 Fourier, *Bruker* AV 300 and *Bruker* AV 400 spectrometers. Chemical shifts are reported in ppm relative to the deuterated solvent. Coupling constants are expressed in Hertz (Hz). The following abbreviations are used: s= singlet, d= doublet, t= triplet and m= multiplet. NMR yields were determined by using mesitylene as internal standard. Elementary analysis was performed on a TruSpec CHMS Micro from *Leco*. IR spectra were recorded on a Nicolet iS10 MIR FT-IR-spectrometer from *Thermo Fisher Scientific*. Thin layer chromatography was performed on *Merck* TLC-plates with fluorescence indication (silica type 60, F_{254}), spots were visualized using UV-light or potassium permanganate. Flash chromatography was performed using silica gel with a grain size of 40–63 µm from *Macherey-Nagel*. Solid state NMR spectra were recorded with a *Bruker* Avance 400 MHz spectrometer operating at 100.56 MHz for ^{13}C, 161.87 MHz for ^{31}P and 399.88 MHz for ^{1}H. All experiments were carried out at a MAS rate of 10 kHz using a 4 mm MAS HX double resonance probe. The ^{1}H and ^{31}P π/2 pulse lengths were 3.1 µs and 2.0 µs, respectively. Two pulse phase modulation (TPPM) heteronuclear dipolar decoupling was used during acquisition. ^{1}H-^{13}C cross polarization magic angle spinning (CP-MAS) NMR experiments were measured using contact time of 2.0 ms for ^{13}C and recycle delays of 2 s. All ^{13}C spectra are referenced to external TMS at 0 ppm using adamantane as a secondary reference. All ^{31}P spectra were measured with recycle delays of 20 s and referenced to a 85% solution of phosphoric acid in water at 0 ppm using ammonium dihydrogen phosphate as a secondary reference. For the plasma immobilization of the catalysts on the support materials by encapsulation in amorphous hydrogenated carbon (a-C:H) coatings, a PECVD process (plasma enhanced chemical vapor deposition) was performed using a Piccolo plasma deposition device from Plasma Electronic GmbH, Neuenburg). The SEM and EDX measurements were performed at a working distance of 9.6 mm at 12.0 keV with a Merlin VP Compact field emission scanning electron microscope (*Carl Zeiss*), respectively. The microscope is equipped with an Inlens Duo-Detector and a HE-SE Detector (high efficiency Everhard Thornley detector) for morphological analysis and a *Bruker* XFlash 6/30 energy dispersive X-ray-spectrometer (EDX spectrometer) for elemental analysis. Sample Preparation: The sample was disposed without any pretreatment on an aluminium stub (12.5 mm diameter, G301F, *Plano GmbH*) with adhesive carbon tape (3347, *Plano GmbH*). After carbon coating (EM SCD 500, *Leica Microsystems*) the sample was transferred to the microscope. In addition, selected samples were sputter-coated with a 10 nm copper layer (Emitech K575, equipped with film-thickness monitor, *Quorum Technologies Ltd.*). After morphological examination elemental mapping was performed on selected grains with surfaces uniformly exposed into the direction of the detector for 300 seconds using a mapping window of 800x600 pixels, in addition an elemental spectrum with 2×10^6 counts was recorded on the grain surface area at higher magnification. SEM images shown in the manuscript (Fig. 5 and 9) are depicted in later section of the supporting information with a scale (SI6.1–

SI6.4). The color intensity ranges used in the EDX mappings are 0–100 for silicon and 0–20 for silicon or phosphorus. Images with a color scale are shown in chapter 5 of the supporting information (SI6.5 and SI6.6). For the quantification of intensity, the following maxima were used: Phosphorus ($K_{\alpha 1}$) at 2.04 keV and iodine ($L_{\alpha 1}$ and $L_{\beta 1}$) at 4.07 keV. Full overview spectra corresponding to the depicted mappings in the manuscript are shown in chapter 4 of the ESI (S3–S12).

2. Preparation and characterization of phosphonium salt catalysts

2.1 Preparation of the homogeneous phosphonium salt catalysts

2-(Diphenylphosphanyl)phenol[1] (3)

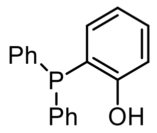

A mixture of *ortho*-iodophenol (660 mg, 3.00 mmol), $Pd(OAc)_2$ (6.70 mg, 0.029 mmol, 0.03 equiv), NaOAc (271 mg, 3.30 mmol, 1.1 equiv) was added in a Schlenk tube and purged with argon. The mixture was dissolved in anhydrous DMA (9.00 mL) and Ph_2PH was added (559 mg, 3.00 mmol, 1.00 equiv) at 25°C. The mixture was heated to 110°C and stirred for 17 h. Afterwards, the reaction mixture was cooled to 25°C and filtered over celite using CH_2Cl_2 as eluent. Subsequently, all volatiles were removed in vacuo. The crude product was then purified via column chromatography (SiO_2, CH_2Cl_2, R_f= 0.52) to give **3** as a colorless solid (710 mg, 2.55 mmol, 83%). ^1H NMR (300 MHz, $CDCl_3$, 25 °C): δ= 6.26–6.28 (br s, 1H), 6.88–7.03 (m, 3H), 7.29–7.39 (m, 11H) ppm. ^{31}P NMR (122 MHz, $CDCl_3$, 25 °C): δ= –28.61 ppm.

Allyl(2-hydroxyphenyl)diphenylphosphonium bromide (5a)

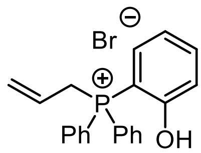

A mixture of **3** (1.49 g, 5.00 mmol) and **4a** (3.02 g, 25.0 mmol) was stirred for 24 h at 70 °C in a pressure tube. The obtained solid was washed with CH_2Cl_2 (3×20 mL) and Et_2O (5×30 mL). Subsequently, all volatiles were removed in vacuo to yield **5a** (1.85 g, 4.63 mmol, 93%) as a colorless solid. ^1H NMR (400 MHz, $CDCl_3$, 25 °C) δ= 4.06 (dd, *J*= 16.5, 7.3 Hz, 2H), 5.35–5.50 (m, 2H), 5.76 (m, 1H), 6.76 (m, 1H), 6.93 (m, 1H), 7.50–7.72 (m, 9H), 7.74–7.82 (m, 2H), 8.11 (m, 1H), 11.28 (br s, 1H) ppm. ^{13}C NMR (75 MHz, $CDCl_3$, 25 °C): δ= 29.36, 100.34, 101.53, 117.98, 118.80, 119.14, 120.38, 120.55, 124.53, 124.98, 125.16, 129.93, 130.01, 132.40, 133.20, 133.33, 133.97, 134.08, 134.70, 137.58, 162.30 ^{31}P NMR (162 MHz, $CDCl_3$, 25 °C): δ= 20.21 ppm. IR Neat: 839.34 (m), 1105.30 (m), 1347.66 (m), 1438.80 (m), 1588.61 (m), 2953 cm^{-1} (br, O–H). LCMS (ESI-MS): *m/z* calcd. $C_{21}H_{20}OP$ [M^+]: 287.0921; *m/z* found $C_{10}H_{20}O_6SiNa$ [M^++Na]: 287.0924.

Allyl(2-hydroxyphenyl)diphenylphosphonium iodide (5b)

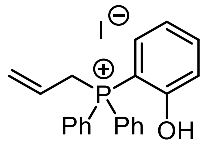

A mixture of **3** (1.50 g, 5.00 mmol) and **4b** (4.20 g, 25.0 mmol) was stirred for 24 h at 25 °C in a pressure tube. The obtained solid was washed with CH_2Cl_2 (3×20 mL) and Et_2O (5×30 mL). Subsequently, all volatiles were removed in vacuo to yield **5b** (2.08 g, 4.70 mmol, 93%) as a yellow solid. ^{1}H NMR (300 MHz, $CDCl_3$, 25 °C): δ = 4.07 (dd, *J*= 16.4, 7.3 Hz, 2H) 5.31–5.57 (m, 2H), 5.70 (d, *J*= 5.7 Hz, 1H), 6.86 (d, *J*= 1.6 Hz, 1H), 6.94–7.03 (m, 1H), 7.57 (s, 10H), 7.73–7.83 (m, 1H), 8.10 (ddd, *J*= 8.5, 5.9, 0.9 Hz, 1H), 9.82 (br s, 1H) ppm. ^{13}C NMR (75 MHz, $CDCl_3$, 25 °C): δ = 29.44, 100.92, 102.11, 117.78, 118.72, 118.18, 118.94, 120.92, 121.09, 124.23, 124.36, 125.16, 125.34, 130.02, 130.18, 133.23, 133.36, 134.77, 134.81, 137.58, 161.55 ppm. ^{31}P NMR (121 MHz, $CDCl_3$, 25 °C): δ = 20.23 ppm. IR Neat: 864.86 (m), 940.14 (m), 1076.51 (w), 1437.54 (m), 1588.91 (m), 2895.45 (m), 3009,5 cm^{-1} (br, O–H). LCMS (ESI-MS): *m/z* calcd. $C_{21}H_{20}OP$ [M^+-I^-]: 319.1252; *m/z* found $C_{21}H_{20}OP$ [M^+-I^-]: 319.0.

(2-Hydroxyphenyl)diphenyl(propyl)phosphonium bromide (5c)[2]

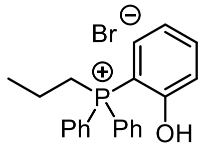

A mixture of **3** (500 mg, 1.80 mmol) and **4c** (1.08 g, 9.00 mmol) was stirred for 24 h at 70 °C in a pressure tube. The obtained solid was washed with CH_2Cl_2 (3×20 mL) and Et_2O (3×30 mL). Subsequently, all volatiles were removed in vacuo to yield **5c** (0.67 g, 1.67 mmol, 93%) as a colorless solid. ^{1}H NMR (300 MHz, $CDCl_3$, 25 °C) δ = 1.11 (t, *J*= 7.3 Hz, 3H), 1.55–1.85 (m, 2H), 3.12 (m, 1H), 6.7–7.04 (m, 2H), 7.48–7.67 (m, 10H), 7.71–7.82 (m, 2H), 8.01–8.14 (m, 1H), 11.11 (s, 1H) ppm. ^{31}P NMR (122 MHz, $CDCl_3$, 25 °C): δ = 23.45 ppm.

(2-Hydroxyphenyl)diphenyl(propyl)phosphonium iodide (5d)[3]

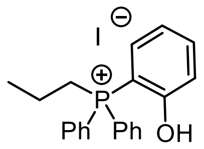

A mixture of **3** (510 mg, 2.55 mmol) and **4d** (2.17 g, 12.8 mmol) was stirred for 24 h at 102 °C in a pressure tube. The obtained solid was washed with CH_2Cl_2 (3×20 mL) and Et_2O (3×30 mL). Subsequently, all volatiles were removed in vacuo to yield **5d** (1.06 g, 2.37 mmol, 93%) as a colorless solid. ^{1}H NMR (300 MHz, $CDCl_3$, 25 °C): δ = 1.07 (t, *J*= 7.3, 3H), 1.66 (m, 2H), 3.08–3.18 (m, 2H), 6.84–6.92 (m, 1H), 6.94–7.01 (m, 1H), 7.53–7.69 (m, 9H), 7.75–7.82 (m, 2H), 7.96–8.01 (m, 1H), 9.93 (br s, 1H) ppm. ^{31}P NMR (122 MHz, $CDCl_3$, 25 °C) δ= 23.46 ppm.

2.2 Procedure for the screening of homogeneous catalyst (Table 1):

A 45 cm^3 stainless-steel autoclave was charged with catalyst **5** (1.0 mol%). Subsequently, 1,2-butylene oxide (**1a**, 1.00 g, 13.9 mmol, 1.0 equiv) was added. The autoclave was purged with CO_2 and heated to 90 °C for 2 h, while $p(CO_2$, 90 °C) was kept constant at 1.0 MPa. The reactor was cooled with an ice bath below 20 °C and CO_2 was released slowly. The conversion of the epoxide **1a** and yield of the carbonate **2a** were determined by ^1H NMR spectroscopy from the reaction mixture using mesitylene as internal standard.

2.3 Procedure for the Impregnation of different supports with catalyst 5b:

Phosphonium salt **5b** (119 mg, 0.278 mmol), was dissolved in CH_2Cl_2 (125 mL). The respective support (TiO_2, FeO or SiO_2, 1.00 g) was added to the solution. The suspension was shaken for 16 h at 23 °C. Subsequently all volatiles were removed in vacuo to obtain the support impregnated with catalyst **5b** (12 wt.% on TiO_2, FeO or SiO_2).

2.4 Amorphous hydrogenated carbon (a-C:H) thin films

Amorphous hydrogenated carbon coatings (a-C:H) are characterized by an irregular network of C atoms, which are partially saturated with hydrogen atoms. It is known that a-C:H coatings have good chemical resistance and undergo degradation only at temperatures greater than 350 °C. A high proportion of hydrogen in the layer leads to large sp^3 C-H bond fractions and rather causes soft layers. By reducing the hydrogen content, the proportion of sp^3 C-C bonds is increased resulting in harder layers (diamond-like). When using methane as a precursor it can be assumed that due to the relatively high H / C ratio, especially soft layers are produced. This is an important feature for the use of the a-C: H coatings as a polymer for encapsulating the catalysts, since this conserves the mechanical parts of the equipment (e.g., circulation pumps) for chemical catalysis and leads to less abrasion.

Amorphous hydrogenated carbon coatings were deposited directly onto the powdery support that has been impregnated with the catalyst before. The polymer deposition was performed in capacitively coupled radio frequency (rf) plasma generated by a rod electrode in vacuum chamber ('Piccolo', Plasma Electronic GmbH, Neuenburg/Germany). A schematic view of the experimental setup is shown in Figure S1.

In this way, the catalyst was partially encapsulated by the plasma polymer layer. For the successful encapsulation with simultaneous accessibility of the catalyst for chemical reactions, the layer thickness of the plasma polymer is crucial. The polymer layer thickness is represented by the nominal layer thickness. This indicates the layer thickness on a smooth, flat surface and was determined in these experiments by profilometer measurements of the plasma polymers on slide glasses.

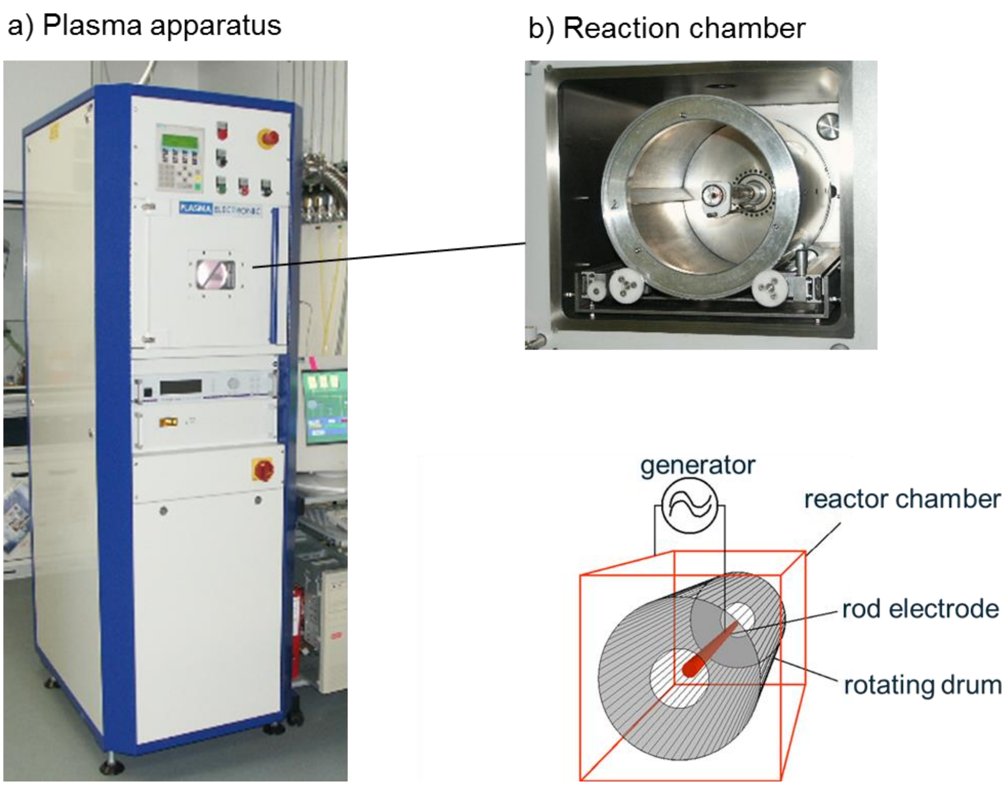

Figure S1 Plasma apparatus.

At above mentioned plasma treatment times of 6.5, 25 and 39 min, respectively, a thickness of 53.3, 136.8 and 190 nm polymer layer has been obtained (Figure S2). These values are determined by depositing the a-C:H coatings on planar glass plates and measuring the coating thickness by profilometry.

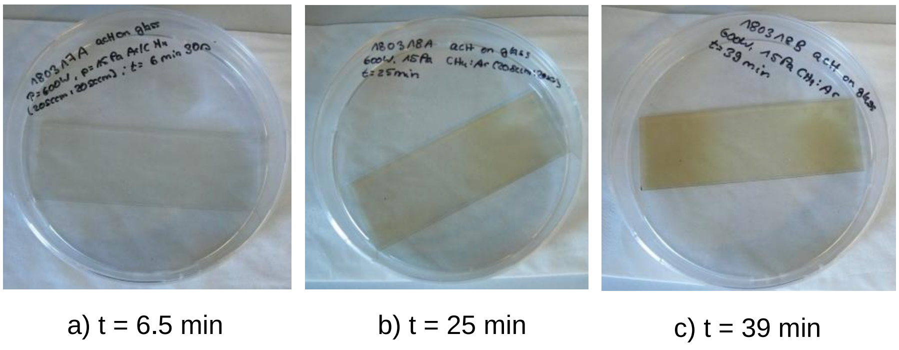

Figure S2 Amorphous hydrogenated carbon coatings (a-C:H) on planar glass plates with (a) 6.5 min, (b) 25 min and (c) 39 min plasma treating times.

2.5 Procedure for the plasma assisted immobilization of catalyst 5b on different supports:

Catalyst **5b** impregnated on TiO_2, FeO or SiO_2 (2.00 g, 12 wt.% **5b**) was dispersed on a sample holder in a vacuum chamber of the plasma deposition device. After a pumping time of about 2 hours, a gas mixture consisting of argon and methane in the ratio 1: 1 (40 sccm) was admitted. After a waiting period

of 5 minutes the plasma power (600 W, 13.56 MHz) was switched on. The pressure of 15 Pa was controlled by pressure gauge and butterfly valve. The plasma treatment time was varied between 6.5, 25 and 39 min.

2.6 NMR spectra for the preparation phosphonium salt catalysts

2.6.1 NMR Spectra for precursor 3 and homogeneous catalysts 5

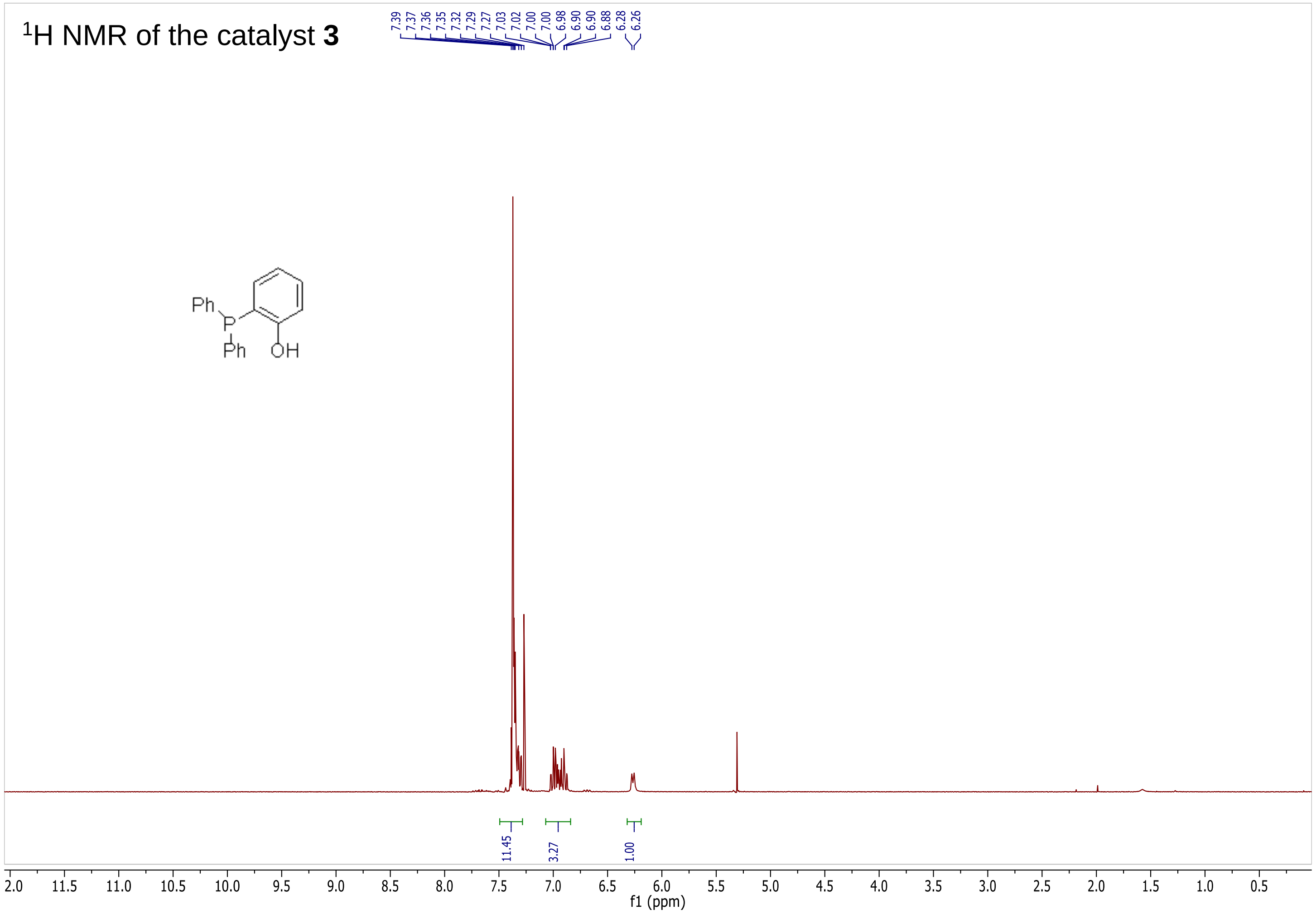

^{31}P NMR of the catalyst **3**

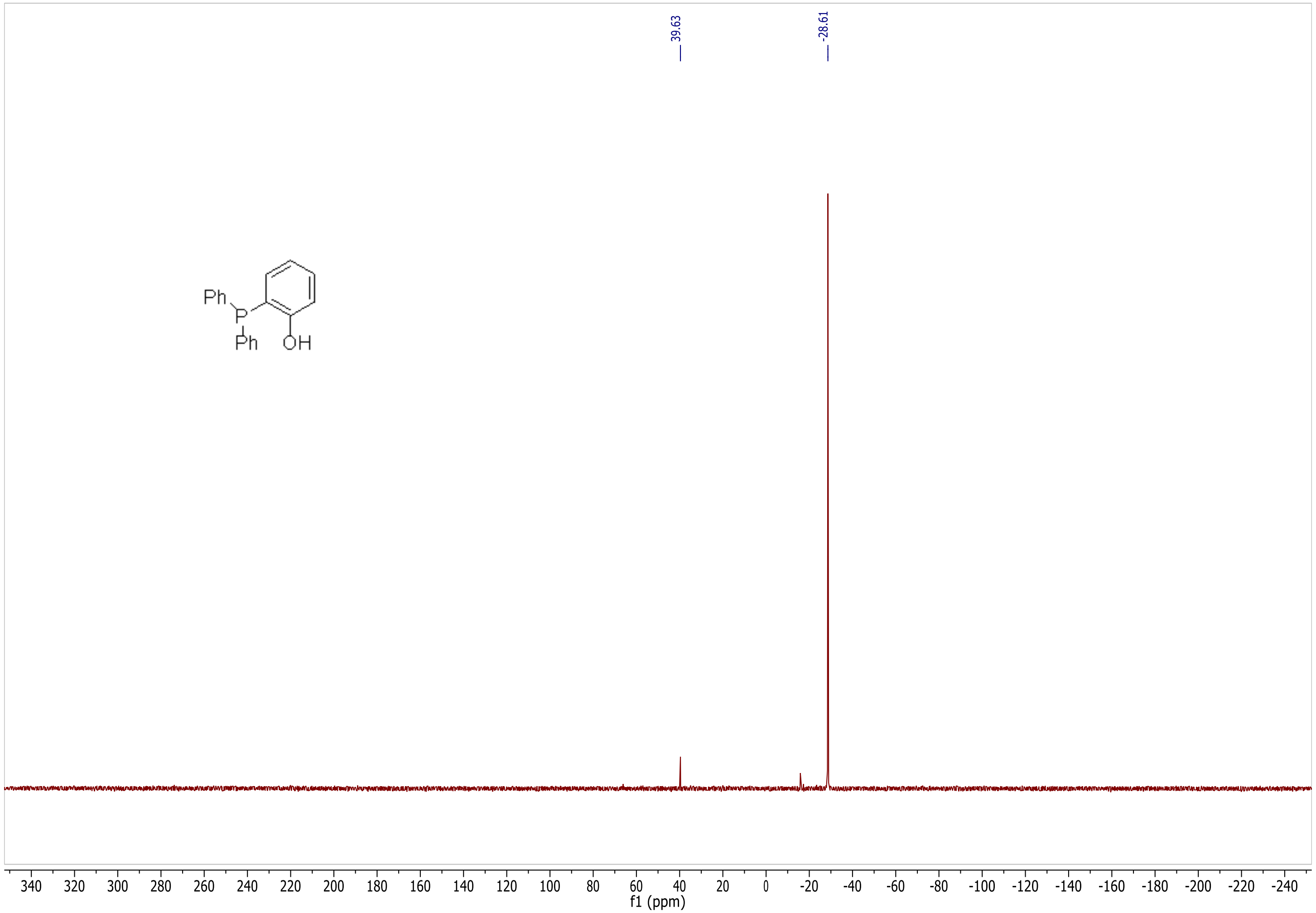

^{1}H NMR of the catalyst **5a**

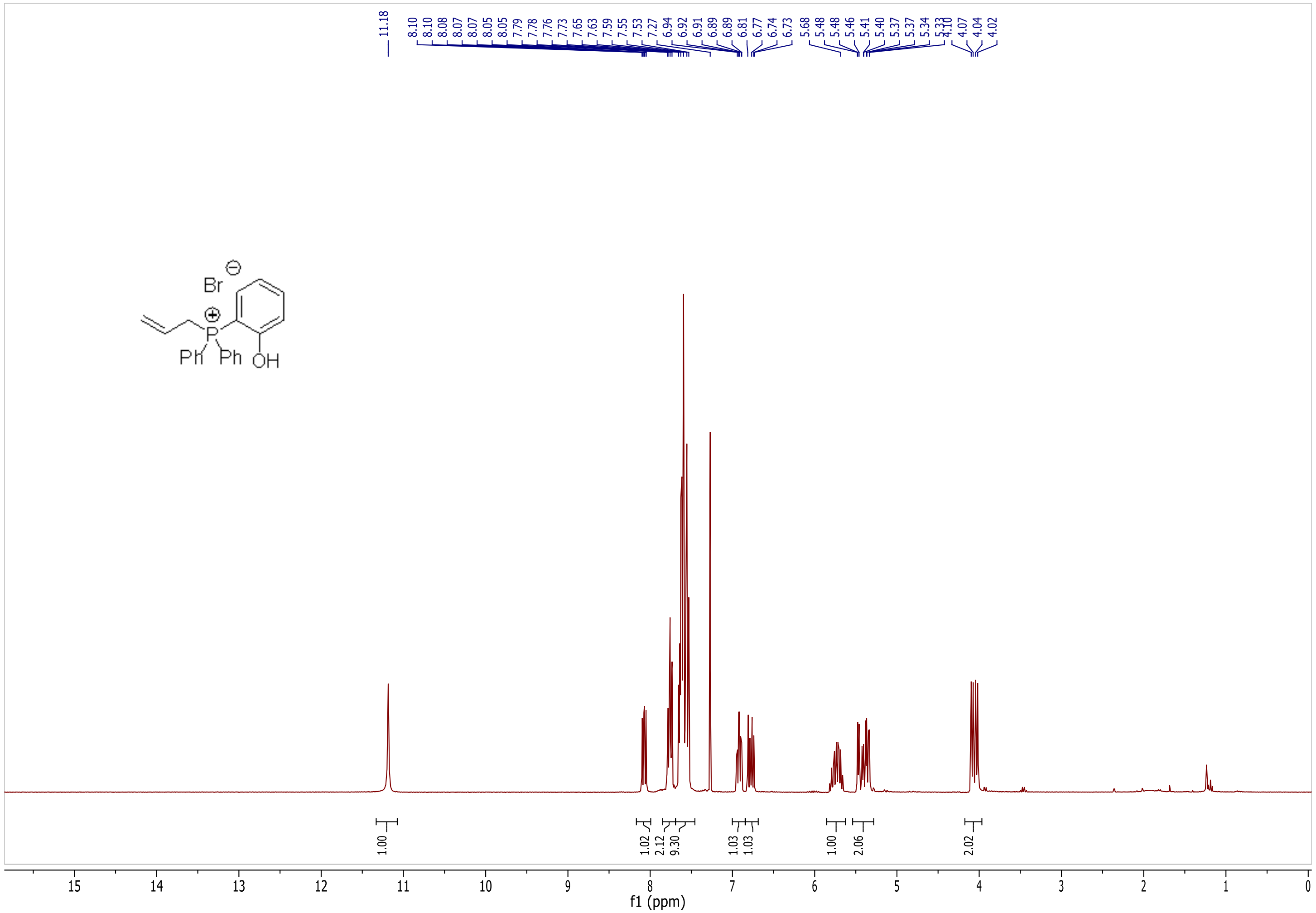

^{13}C NMR of the catalyst **5a**

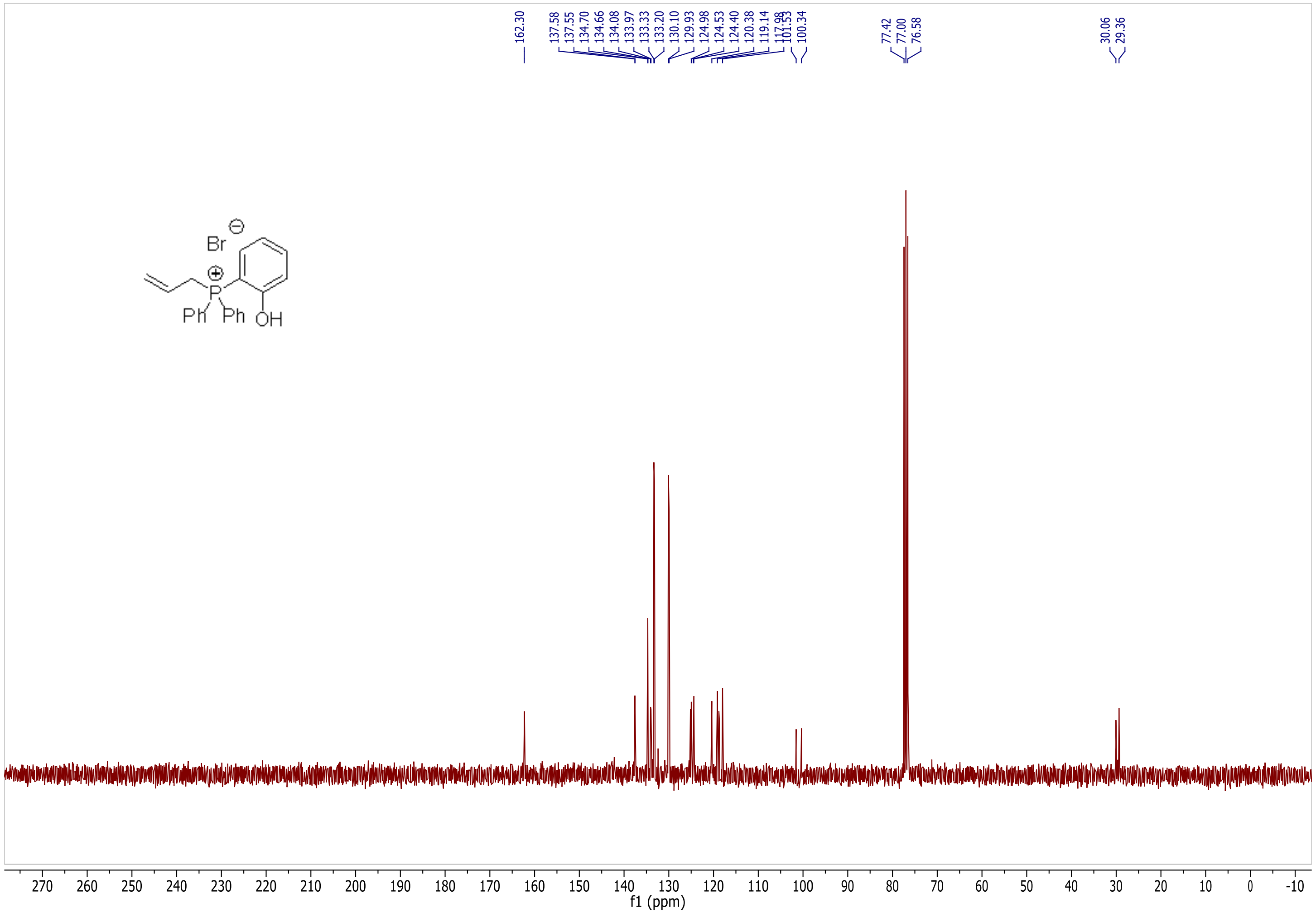

^{31}P NMR of the catalyst **5a**

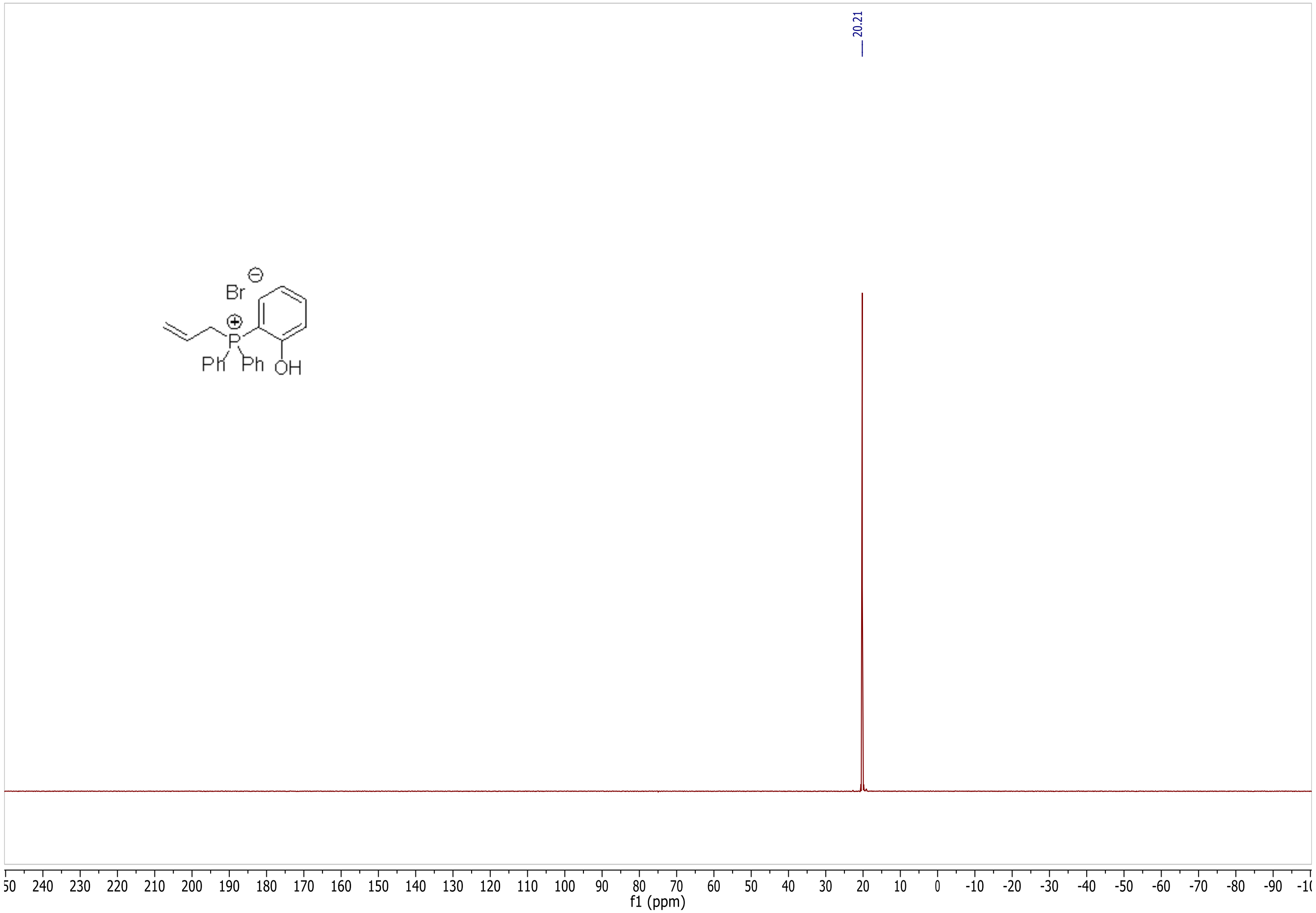

^{1}H NMR of the catalyst **5b**

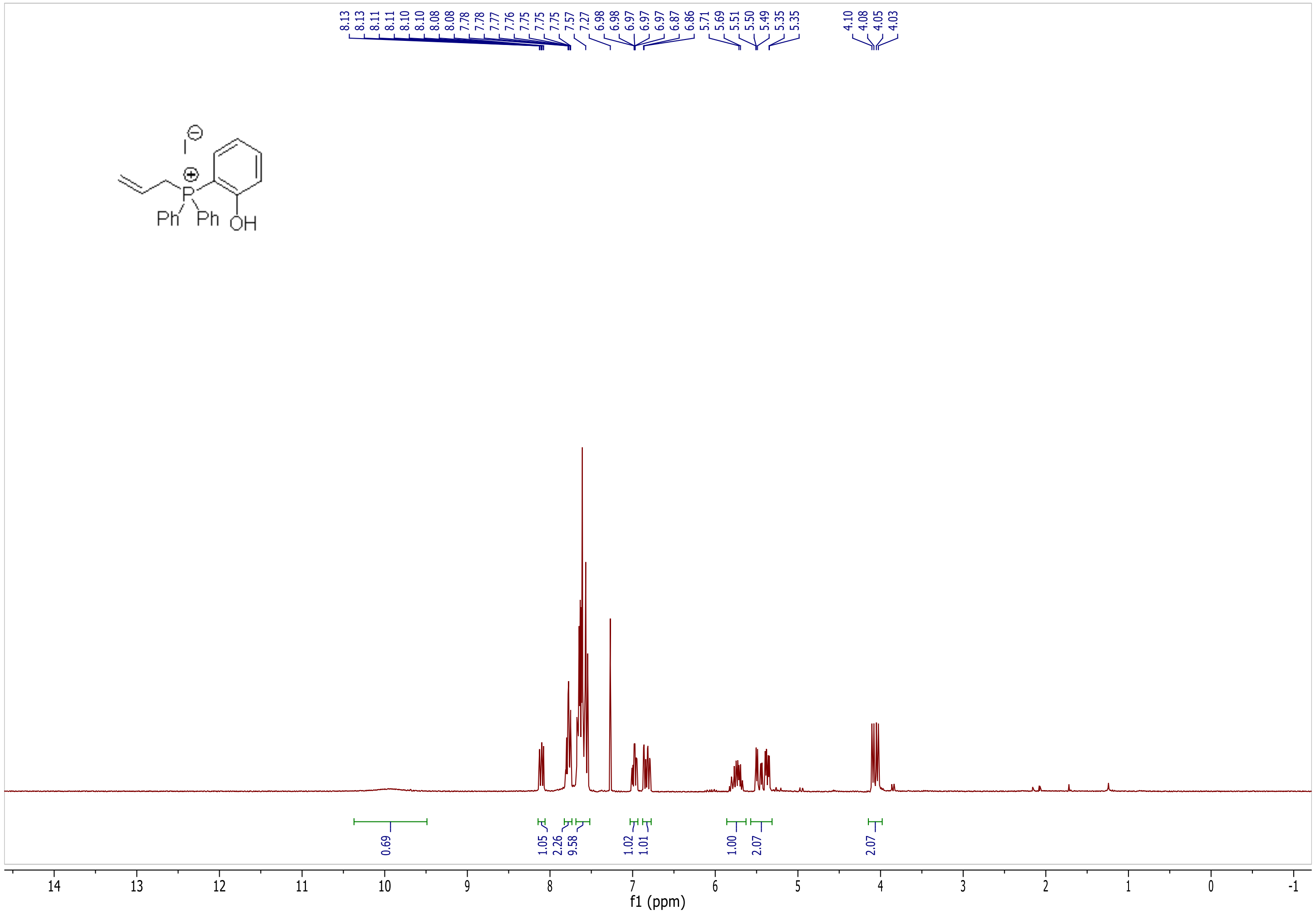

^{13}C NMR of the catalyst **5b**

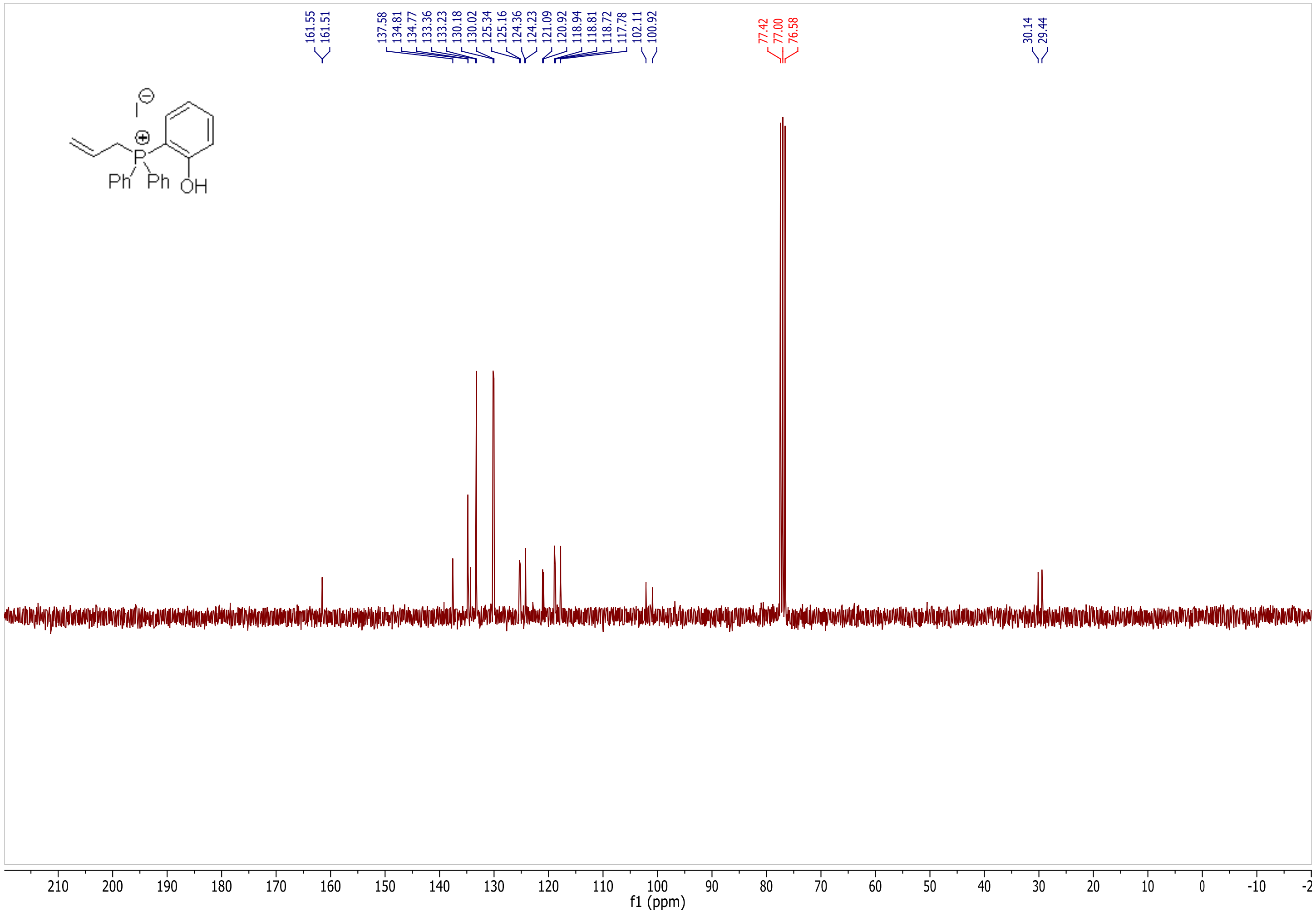

^{31}P NMR of the catalyst **5b**

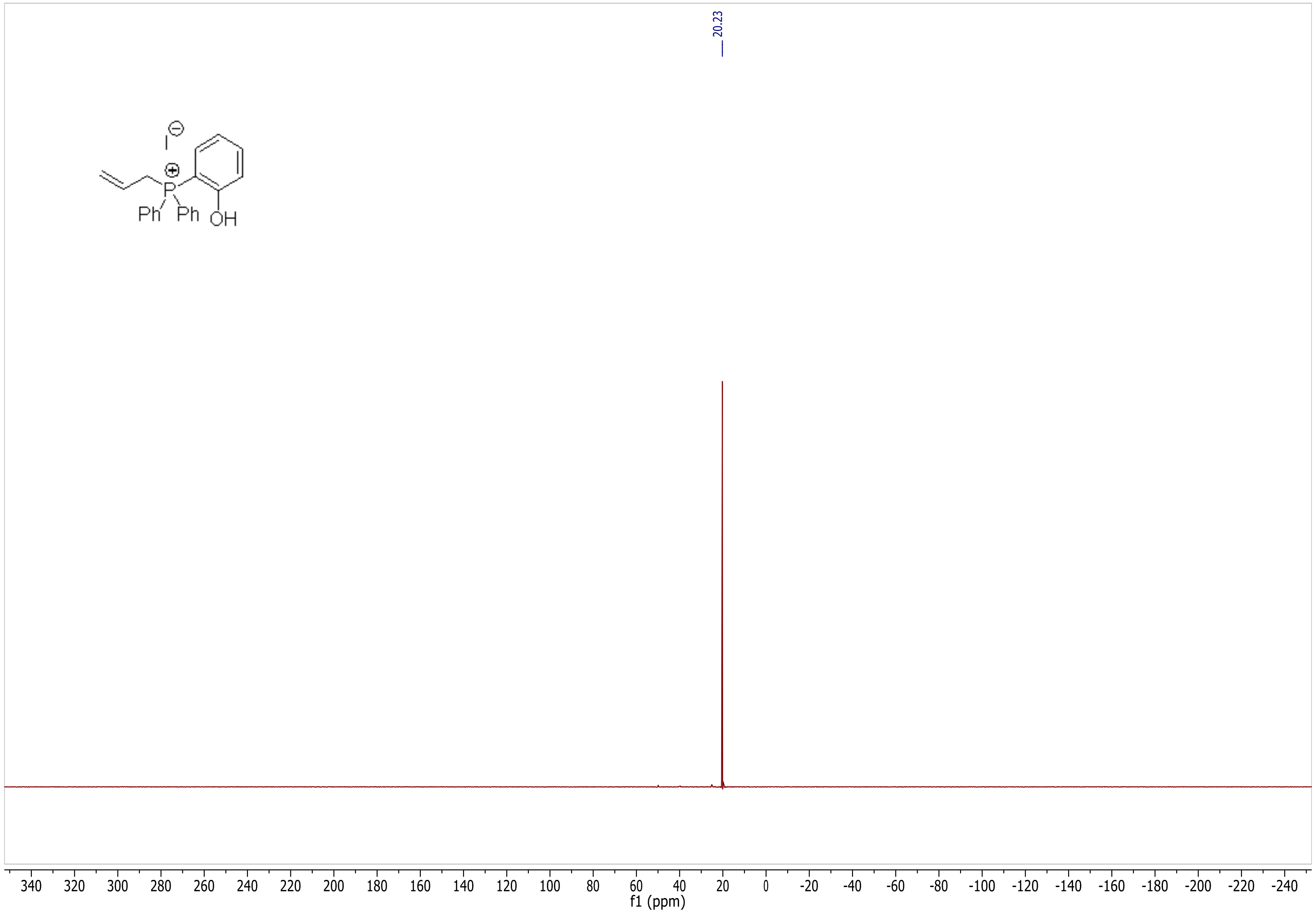

^{1}H NMR of the catalyst **5c**

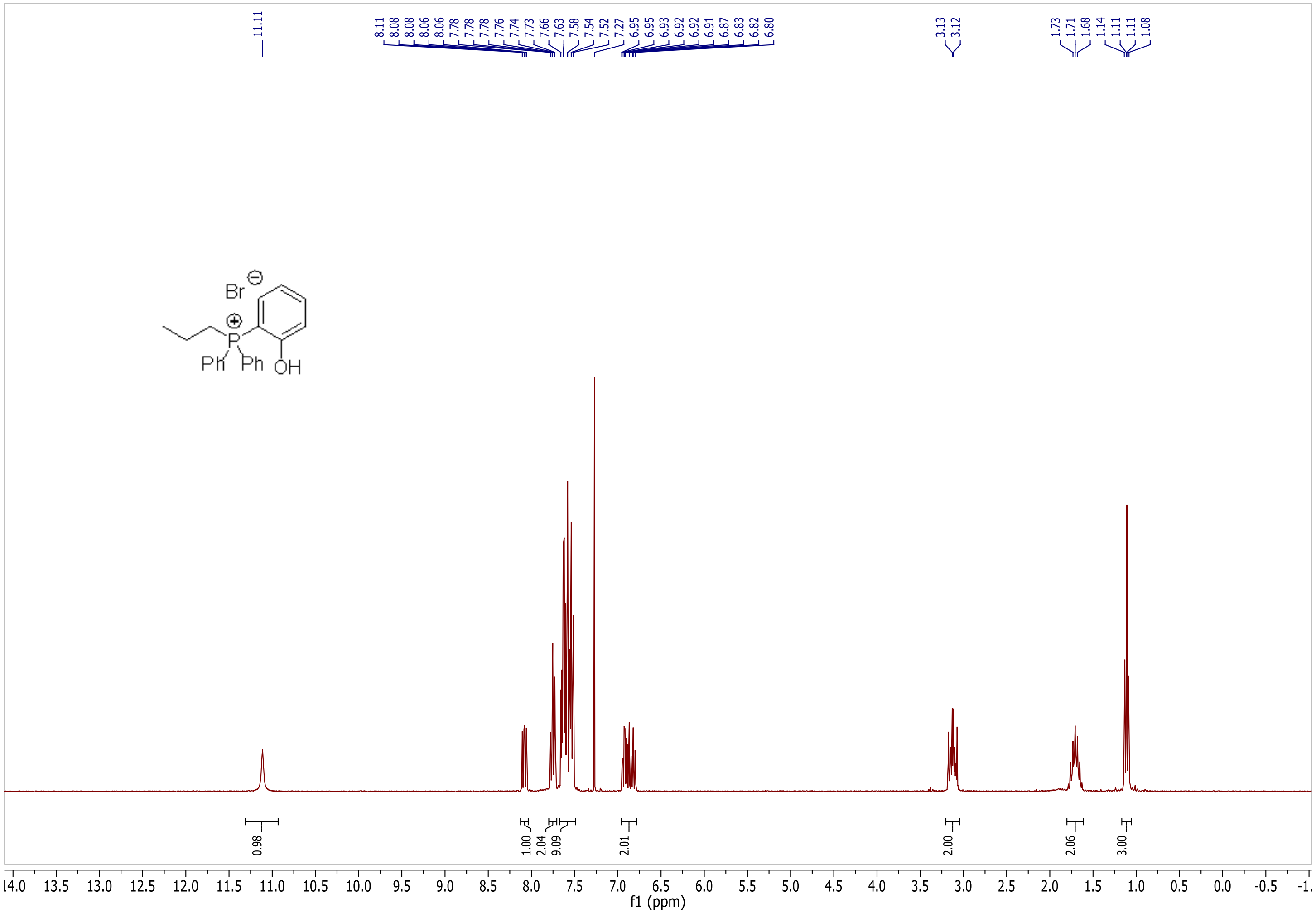

^{31}P NMR of the catalyst **5c**

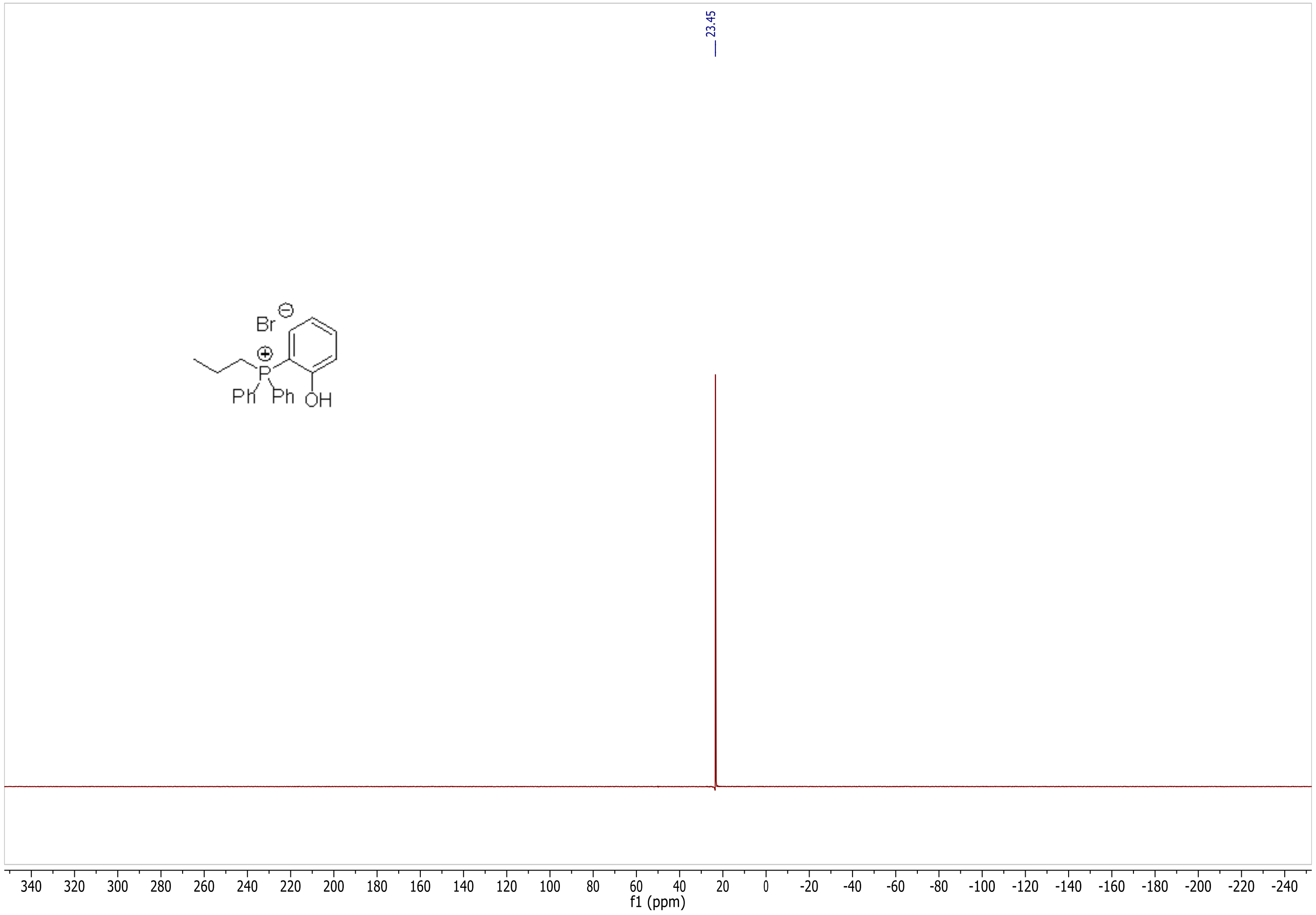

^{1}H NMR of the catalyst **5d**

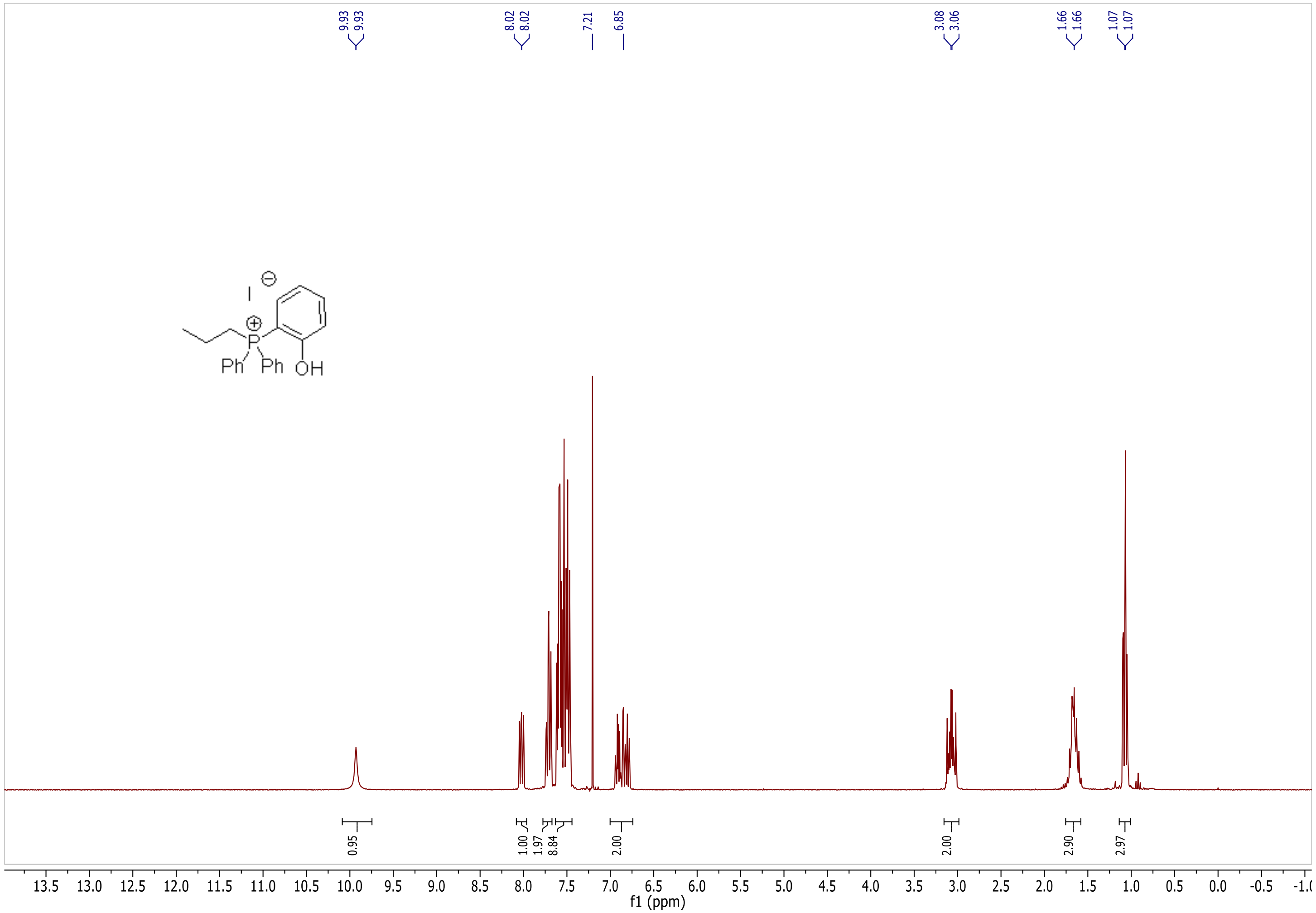

^{31}P NMR of the catalyst **5d**

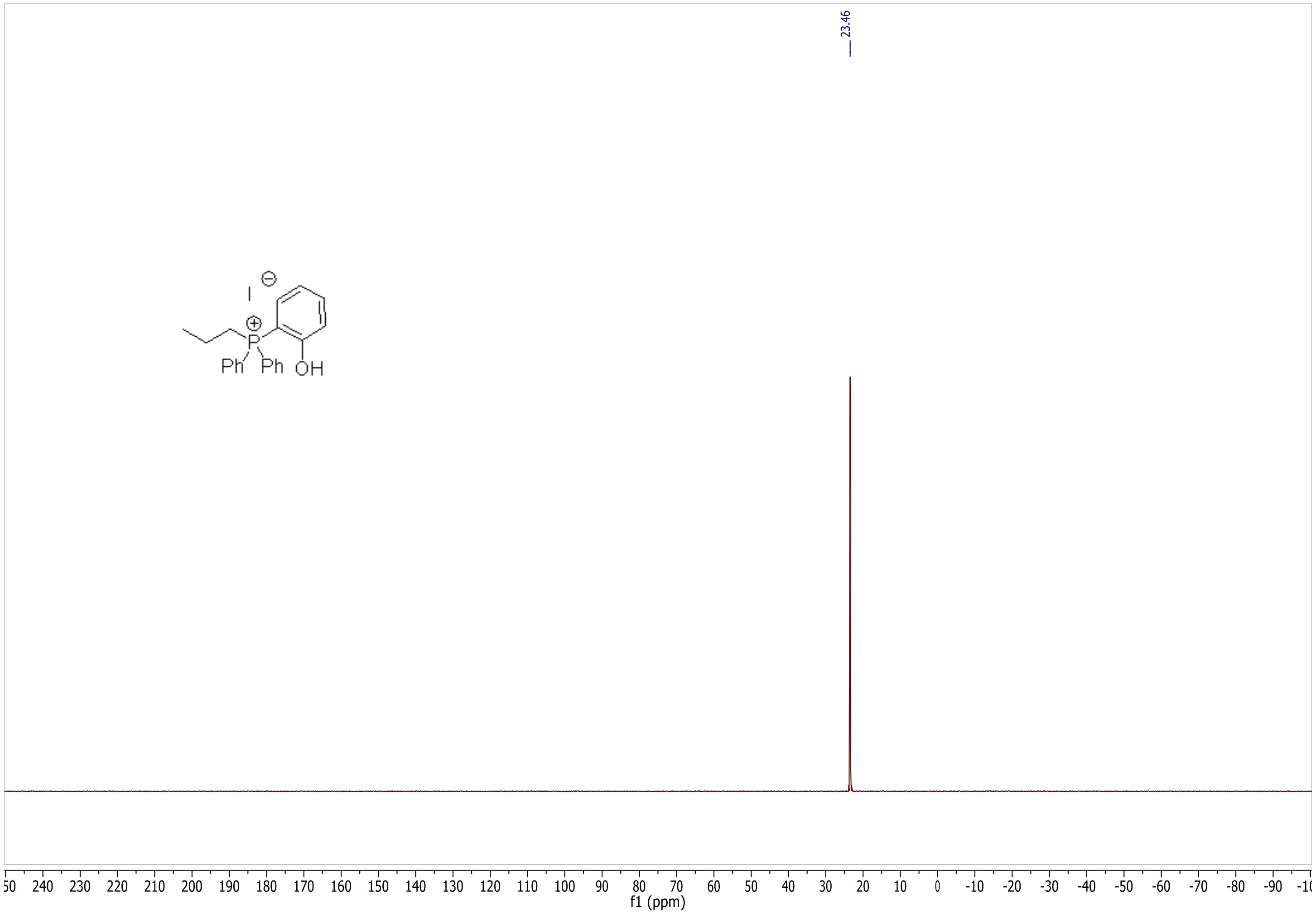

2.6.2 Solid state ^{13}C NMR and ^{31}P NMR of impregnated catalyst 5b@SiO$_2$ and plasma treated catalyst 5bb℗SiO$_2$

Solid state ^{13}C NMR of impregnated catalyst 5b@SiO$_2$

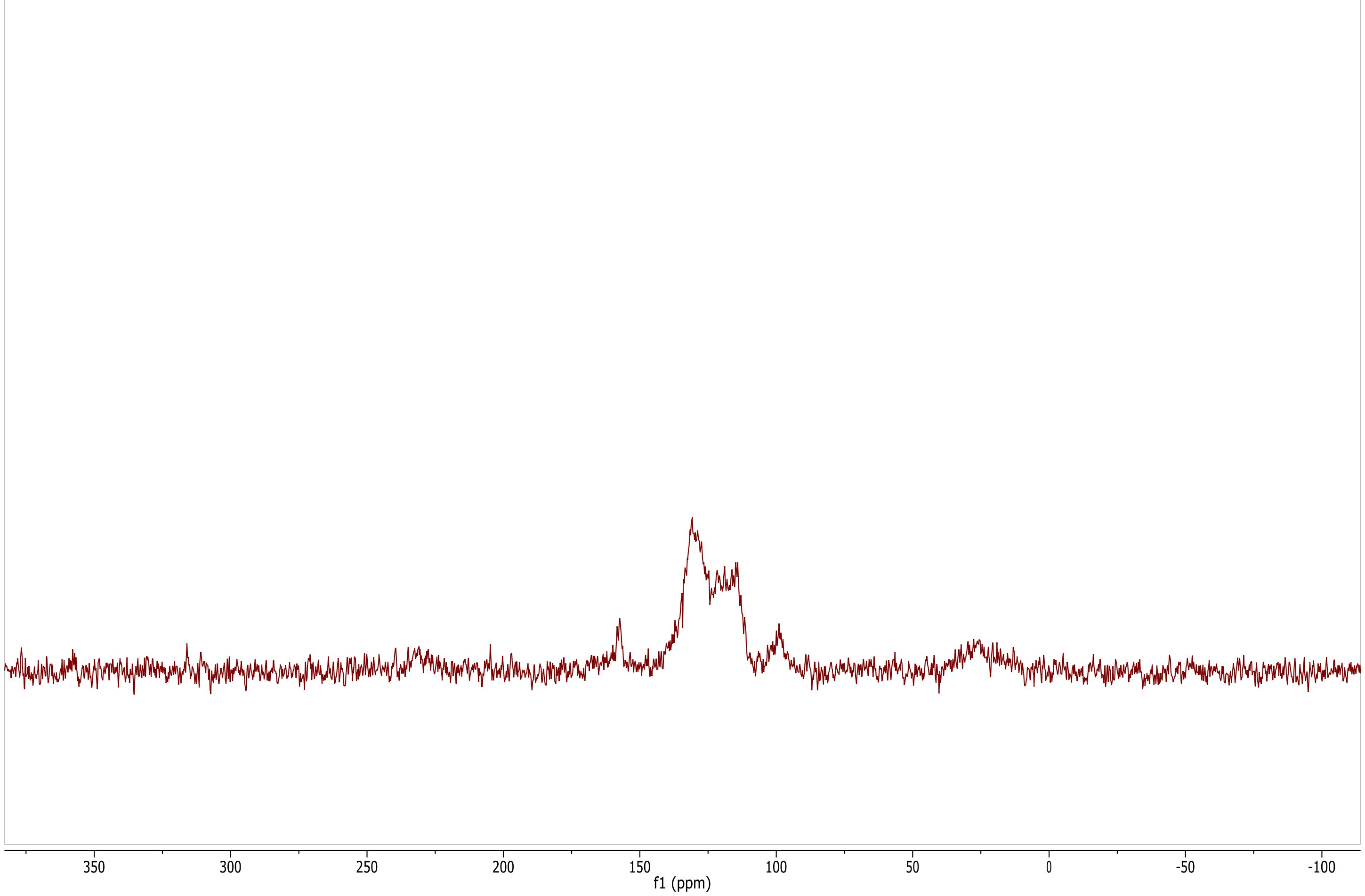

Solid state ^{31}P NMR of impregnated catalyst 5b@SiO_2

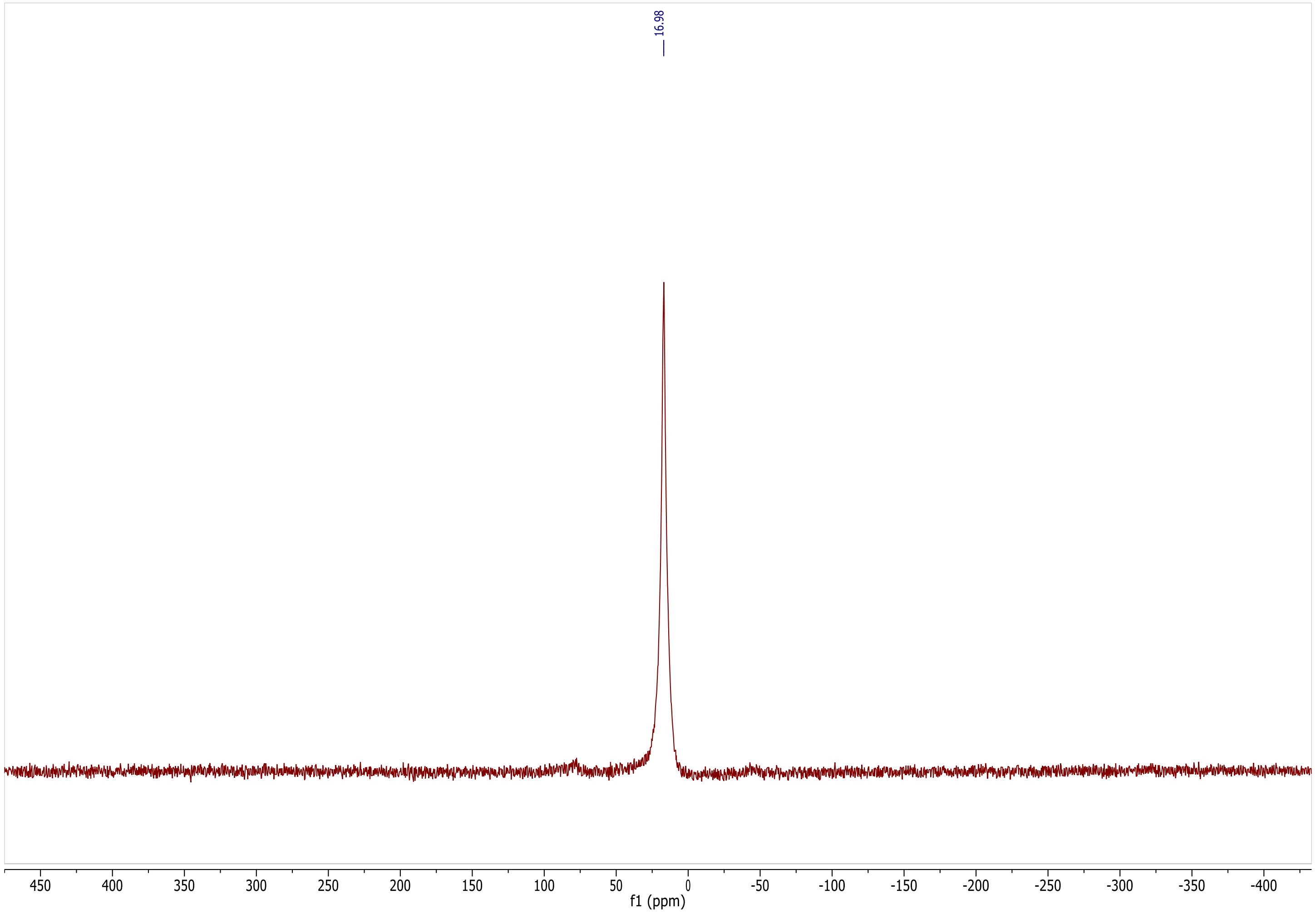

Solid state ^{13}C NMR of plasma treated catalyst 5bb℗SiO_2

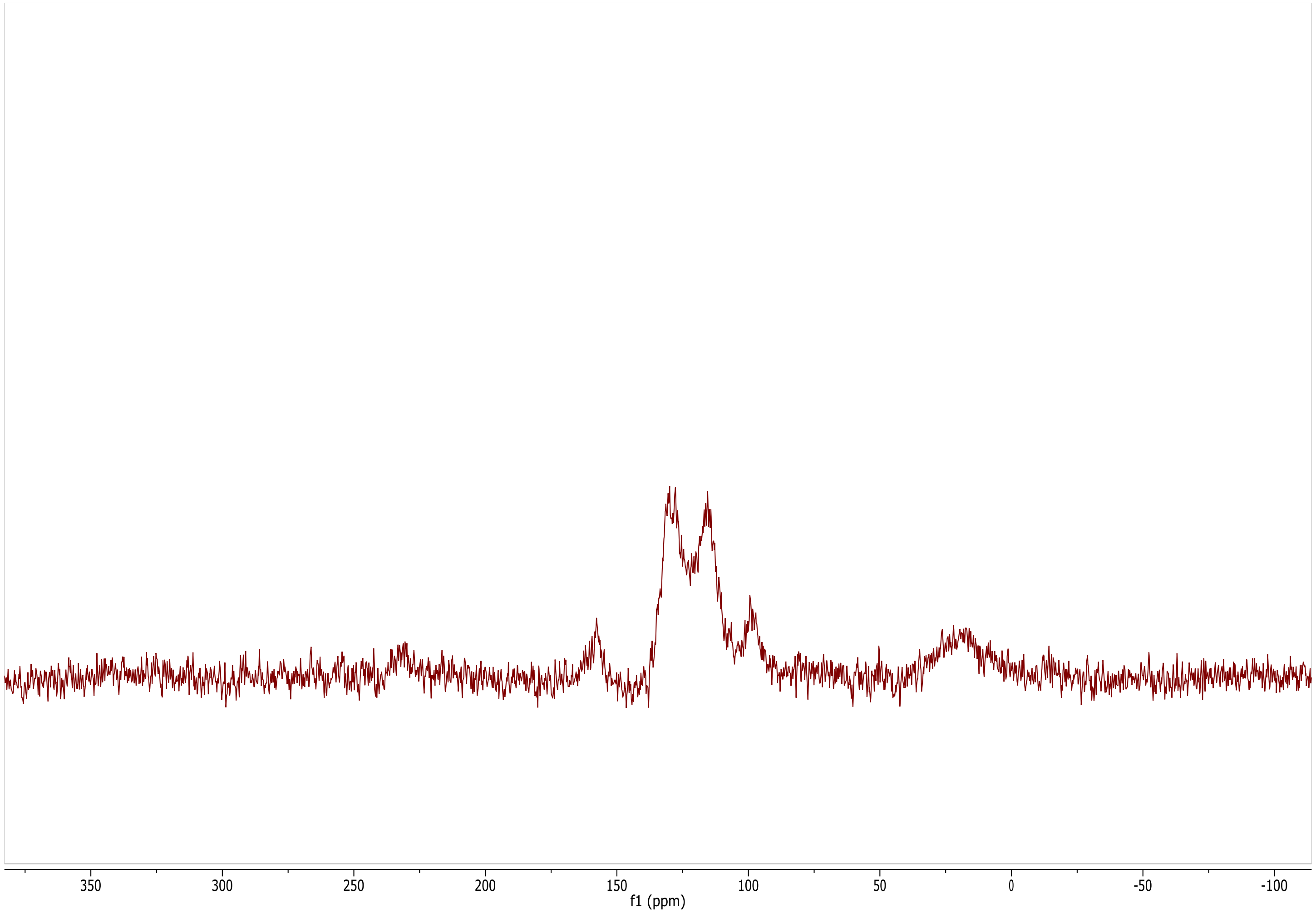

Solid state ^{31}P NMR of plasma treated catalyst 5bb®SiO$_2$

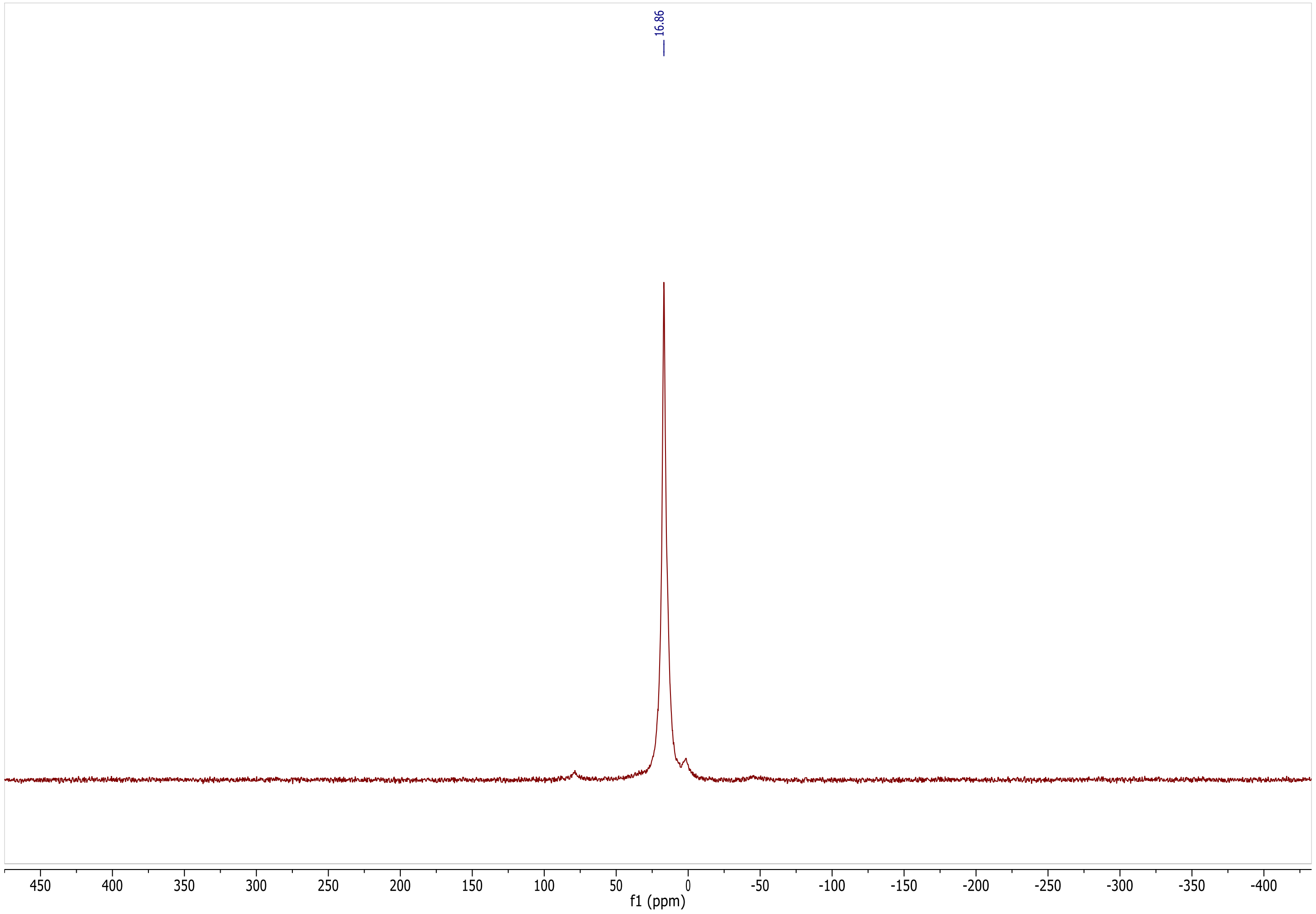

3. Experimental procedure for catalyst, parameter, substrate screening and catalyst recycling

3.1 Catalyst and parameter screening (Table 2 and Table 3):

A 45 cm^3 stainless-steel autoclave was charged with the impregnated or plasma treated catalyst (500 mg, 1.0 mol% or 2.0 mol%) and 1,2-butylene oxide (**1a**, 1.00 g, 13.9 mmol, 1.0 equiv). The autoclave was purged with CO_2 and the reactor was heated to 45 °C or 90 °C for 3–24 h, while $p(CO_2$, 90 °C) was kept constant at 1.0 MPa. The reactor was cooled with an ice bath below 20 °C and CO_2 was released slowly. The conversion of the epoxide **1a** and the yield of the carbonate **2a** were determined by ^1H NMR spectroscopy from the reaction mixture using mesitylene as internal standard.

3.2 Protocol for the catalyst recycling experiments:

A 45 cm^3 stainless-steel autoclave was charged with the catalyst **5bb**ⓅSiO₂ (500 mg, 1.0 mol% or 2.0 mol% loading), 1,2-butylene oxide (**1a**, 1.0 g,13.9 mmol, 1.0 equiv). The autoclave was purged with CO_2 and heated to 45°C or 90 °C for 2 h or 6 h, while $p(CO_2$, 90 °C) was kept constant at 1.0 MPa. Subsequently the reactor was cooled to ≤20 °C with an ice bath and CO_2 was released slowly. The reaction mixture was removed by extraction with Et_2O (3×30 mL). All volatiles were removed in vacuo to yield 1,2-butylene carbonate **2a**. The catalyst was dried in air overnight and reused. The conversion of the epoxide **1a** and yield of the desired carbonate were determined either with isolated product or by ^1H NMR spectroscopy using mesitylene as internal standard.

Table S1 Recycling experiments (1st and 2nd run) for various plasma treating times.

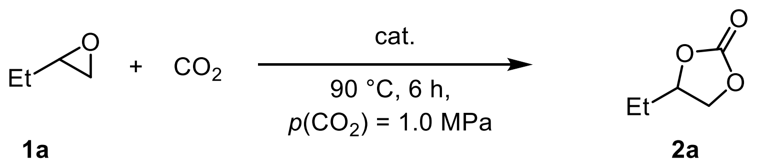

Entry	Cat.	Recycling run	Impregnated catalyst loading / mol%	Plasma treating time / min	Yield 2a / %
1	**5b**ⓅTiO_2	1st	1	6.5 / 25 / 39	95 / 93 / 85
2	**5b**ⓅFeO	1st	1	6.5 / 25 / 39	85 / 72 / 85
3	**5b**ⓅSiO_2	1st	1	6.5 / 25 / 39	98 / >99 / >99
4	**5b**ⓅTiO_2	2nd	1	6.5 / 25 / 39	45 / 56 / 16
5	**5b**ⓅFeO	2nd	1	6.5 / 25 / 39	3 / 2 / 20
6	**5b**ⓅSiO_2	2nd	1	6.5 / 25 / 39	82 / >99 / >99

Reaction conditions: 45 cm^3 stainless-steel autoclave, **1a** (13.9 mmol, 1.0 equiv), 500 mg of the immobilized catalyst (1.0 mol% loading), solvent-free. Yield determined by ^1H NMR with mesitylene as internal standard.

Table S2 Recycling experiments (1st and 2nd run) with impregnated catalyst on SiO_2

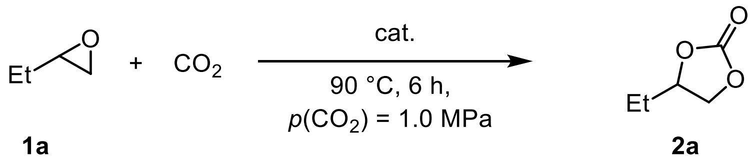

1a **2a**

Entry	Support	Recycling run	Impregnated catalyst loading / mol%	Yield 2a / %
1	**5b**@TiO_2	1st	1	87
2	**5b**@TiO_2	2nd	1	19
3	**5b**@FeO	1st		78
4	**5b**@FeO	2nd		0
5	**5b**@SiO_2	1st	1	88
6	**5b**@SiO_2	2nd	1	31

Reaction conditions: 45 cm^3 stainless-steel autoclave, **1a** (13.9 mmol, 1.0 equiv), 500 mg of the impregnated catalyst (1.0 mol% loading), solvent-free. Yield determined by ^1H NMR with mesitylene as internal standard.

Table S3 Recycling experiments (5 runs) for various plasma treating time with SiO_2 as the support.

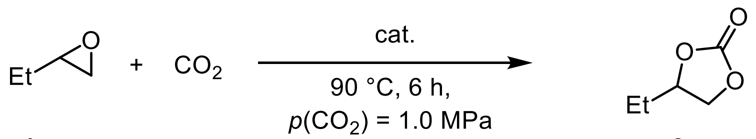

1a **2a**

Entry	Support	Recycling run	Impregnated catalyst loading / mol%	Plasma treating time / min	Yield 2a / %
1	**5b**ⓅSiO_2	1st	1	6.5 / 25 / 39	98 / >99 / >99
2	**5b**ⓅSiO_2	2nd	1	6.5 / 25 / 39	82 / >99 / >99
3	**5b**ⓅSiO_2	3rd	1	6.5 / 25 / 39	74 / 81 / 70
4	**5b**ⓅSiO_2	4th	1	6.5 / 25 / 39	43 / 50 / 38
5	**5b**ⓅSiO_2	5th	1	6.5 / 25 / 39	20 / 20 / 15

Reaction conditions: 45 cm^3 stainless-steel autoclave, **1a** (13.9 mmol, 1.0 equiv), 500 mg of the immobilized catalyst (1.0 mol% loading), solvent-free. Yield determined by ^1H NMR with mesitylene as internal standard.

Table S4 Recycling experiments (5 runs) for different plasma treating methods with SiO_2 as the support.

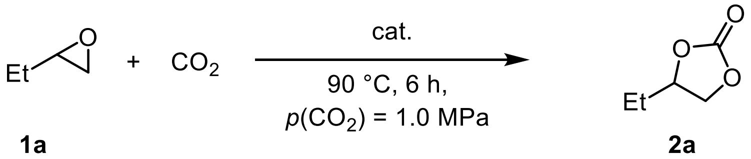

Entry	Support	Recycling run	Impregnated catalyst loading / mol%	Plasma treating time / min	Yield 2a / %
1	**5b**®SiO_2	1st	1	12 + 13 / 25	88 / >99
2	**5b**®SiO_2	2nd	1	12 + 13 / 25	84 / >99
3	**5b**®SiO_2	3rd	1	12 + 13 / 25	75 / 81
4	**5b**®SiO_2	4th	1	12 + 13 / 25	38 / 50
5	**5b**®SiO_2	5th	1	12 + 13 / 25	22 / 20

Reaction conditions: 45 cm^3 stainless-steel autoclave, **1a** (13.9 mmol, 1.0 equiv), 500 mg of the immobilized catalyst (1.0 mol% loading), solvent-free. Yield determined by ^1H NMR with mesitylene as internal standard.

Table S5 Recycling experiments (5 runs) for optimization of reaction time with SiO_2 as the support.

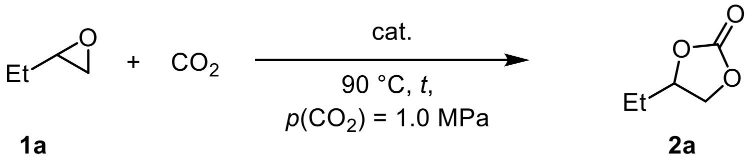

Entry	Support	Recycling run	Impregnated catalyst loading / mol%	Reaction time *t* / h	Yield 2a / %
1	**5b**®SiO_2	1st	1	3 / 6	88 / >99
2	**5b**®SiO_2	2nd	1	3 / 6	84 / >99
3	**5b**®SiO_2	3rd	1	3 / 6	75 / 81
4	**5b**®SiO_2	4th	1	3 / 6	38 / 50
5	**5b**®SiO_2	5th	1	3 / 6	22 / 20

Reaction conditions: 45 cm^3 stainless-steel autoclave, **1a** (13.9 mmol, 1.0 equiv), 500 mg of the immobilized catalyst (1.0 mol% loading), solvent-free. Yield determined by ^1H NMR with mesitylene as internal standard.

Table S6 Recycling experiments (5 runs) for optimization of reaction temperature with SiO_2 as the support.

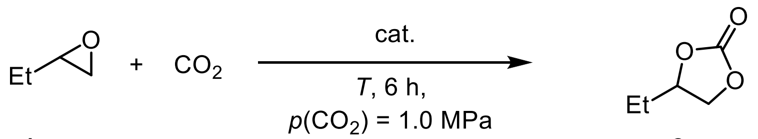

1a **2a**

Entry	Support	Recycling run	Impregnated catalyst loading / mol%	*T* / °C	Yield 2a / %
1	**5b**℗SiO_2	1st	1	45 / 90	>99 / >99
2	**5b**℗SiO_2	2nd	1	45 / 90	81 / >99
3	**5b**℗SiO_2	3rd	1	45 / 90	46 / 81
4	**5b**℗SiO_2	4th	1	45 / 90	23 / 50
5	**5b**℗SiO_2	5th	1	45 / 90	5 / 20

Reaction conditions: 45 cm^3 stainless-steel autoclave, **1a** (13.9 mmol, 1.0 equiv), 500 mg of the immobilized catalyst (1.0 mol% loading), solvent-free. Yield determined by ^1H NMR with mesitylene as internal standard.

Table S7 Recycling experiments (5 runs) for optimization of reaction temperature with TiO_2 and SiO_2 as the support.

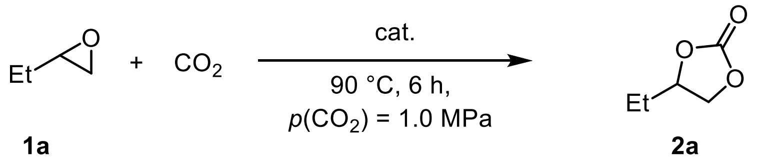

1a **2a**

Entry	Support	Recycling run	Impregnated catalyst loading / mol%	Yield 2a / %
1	**5b**℗TiO_2	1st	2 /1	81 / 93
2	**5b**℗TiO_2	1st	2 /1	80 / 56
3	**5b**℗SiO_2	1st	2 /1	93 / >99
4	**5b**℗SiO_2	2nd	2 /1	93 / >99
5	**5b**℗SiO_2	3rd	2 /1	90 / 81
6	**5b**℗SiO_2	4th	2 /1	59 / 50
7	**5b**℗SiO_2	5th	2 /1	41 / 20

Reaction conditions: 45 cm^3 stainless-steel autoclave, **1a** (13.9 mmol, 1.0 equiv), 500 mg of the immobilized catalyst (1.0 mol% or 2 mol% loading), solvent-free. Yield determined by ^1H NMR with mesitylene as internal standard.

3.3 Solid state ^{13}C NMR and ^{31}P NMR recycled catalyst 5bb®SiO$_2$ after 5 runs.

Solid state ^{13}C NMR of the catalyst **5bb®SiO$_2$** after 5 runs

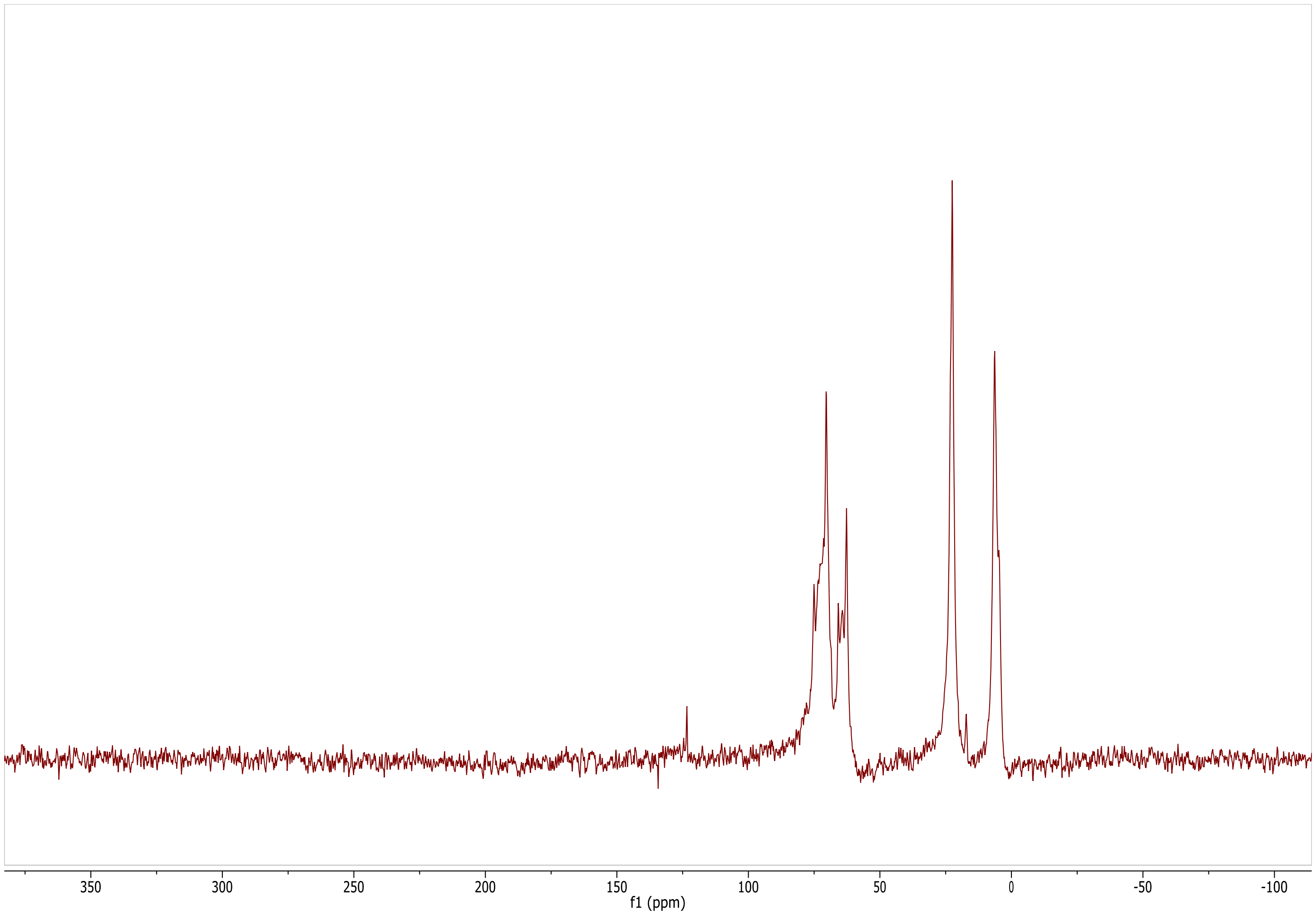

Solid state ^{31}P NMR of the catalyst **5bb®SiO$_2$** used after 5 runs

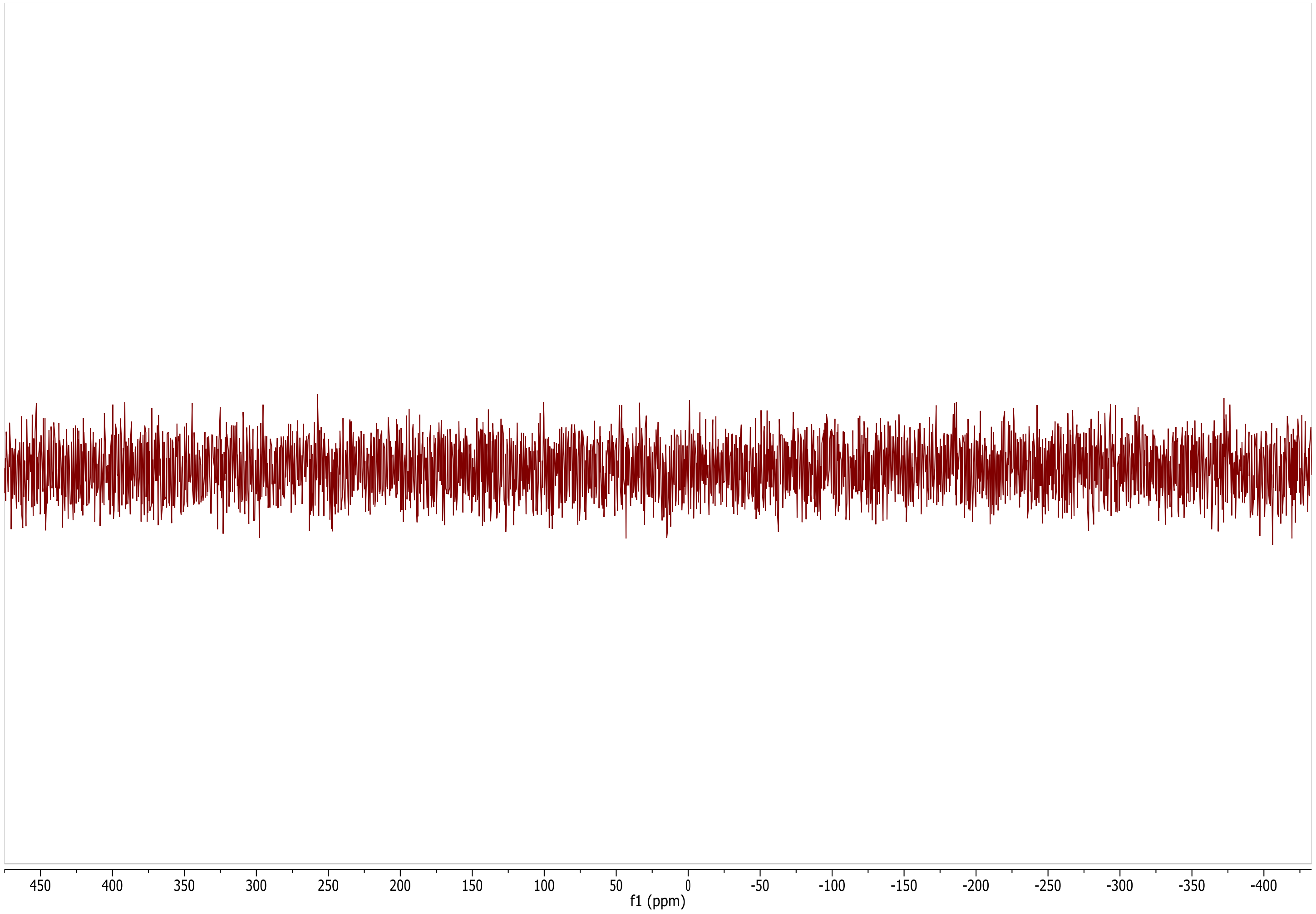

4. SEM images and EDX mappings

The EDX mapping was performed within the boundary of the green frame (mapping area of 800×600 Pixel) to identify only elements within the central area of the corn.

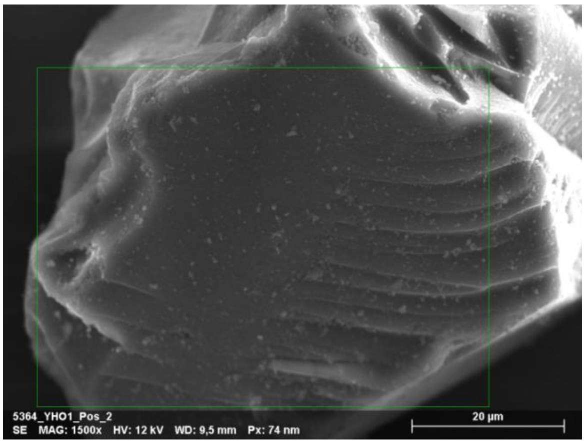

Figure S3 SEM image of SiO_2 support with scale (see also Figure 6, Ia in the manuscript).

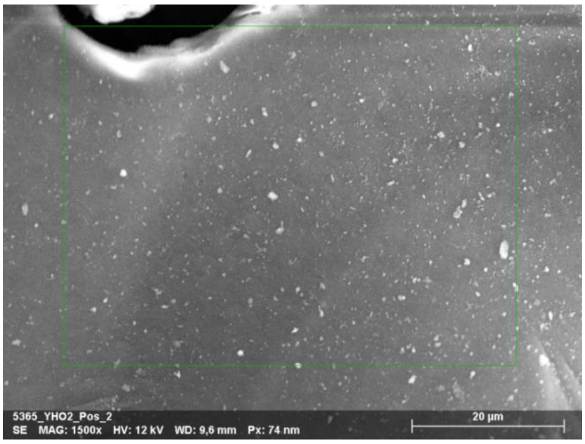

Figure S4 SEM image of impregnated catalyst **5b**@SiO_2 with scale.

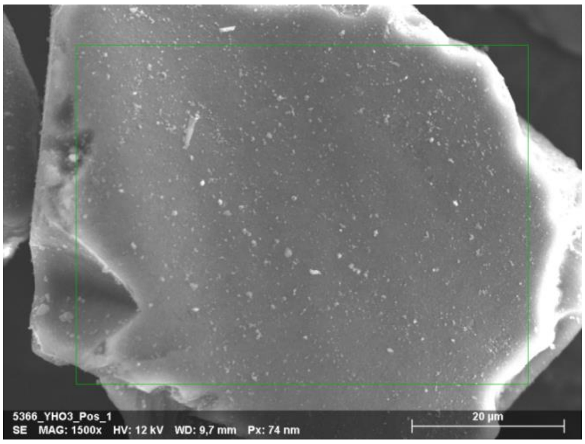

Figure S5 SEM image of plasma immobilized catalyst **5bb**℗SiO_2 with scale (see also Fig. 6, IIa in the manuscript).

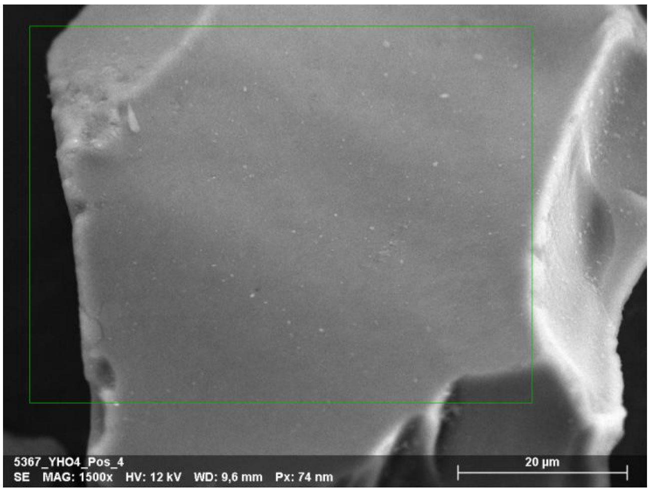

Figure S6 SEM image of plasma immobilized catalyst **5bb®SiO$_2$** after 5 cycles with scale.

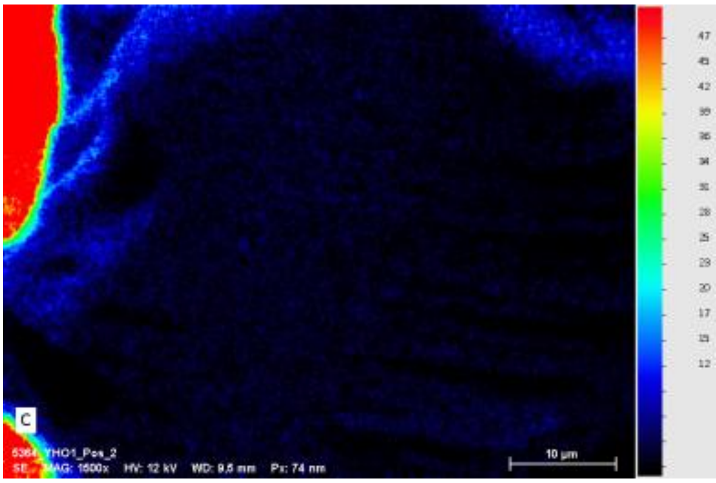

Figure S7 EDX mapping with color coded intensity range for carbon of SiO$_2$ support with color scale (see also Fig. 6, Ib in the manuscript).

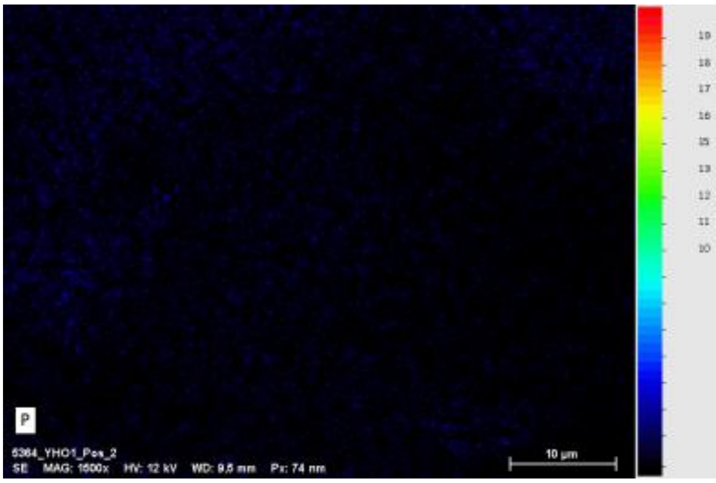

Figure S8 EDX mapping with color coded intensity range for phosphorus of SiO$_2$ support with color scale (see also Fig. 6, Ic in the manuscript).

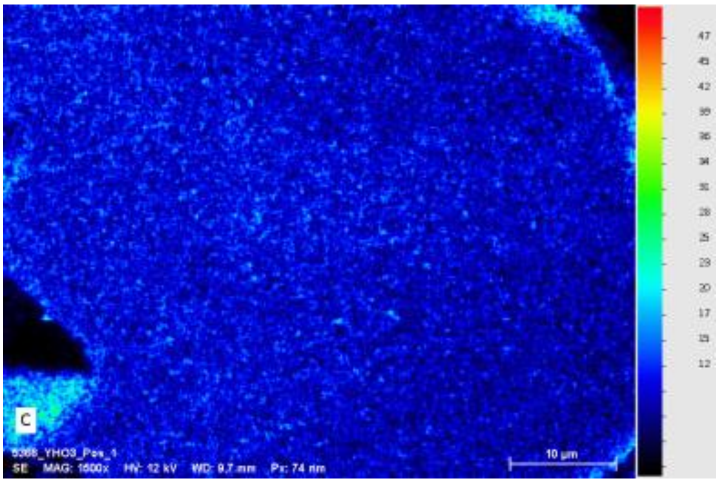

Figure S9 EDX mapping with color coded intensity range for carbon of plasma treated catalyst **5bb**®SiO_2 with color scale (see also Fig. 6, IIb in the manuscript).

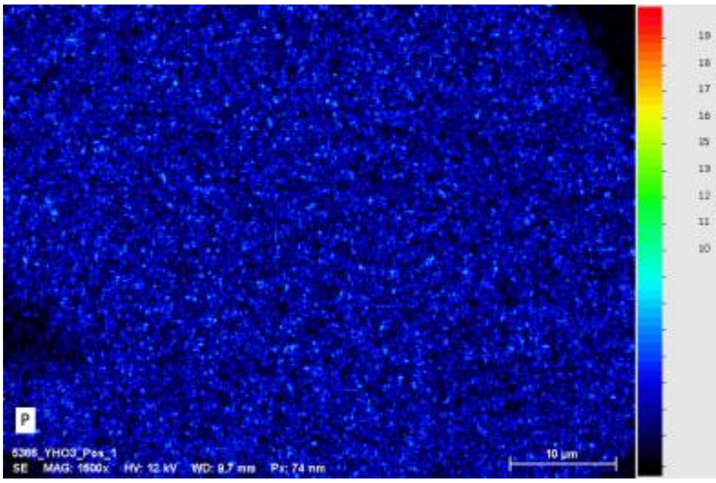

Figure S10 EDX mapping with color coded intensity range for phosphorus of plasma treated catalyst **5bb**®SiO_2 with color scale (see also Fig. 6, IIc in the manuscript).

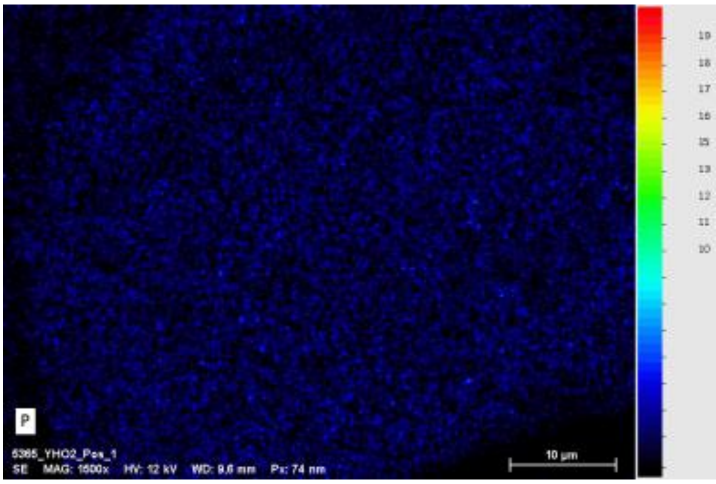

Figure S11 EDX mapping with color coded intensity range for phosphorus of catalyst **5b@SiO_2** with color scale.

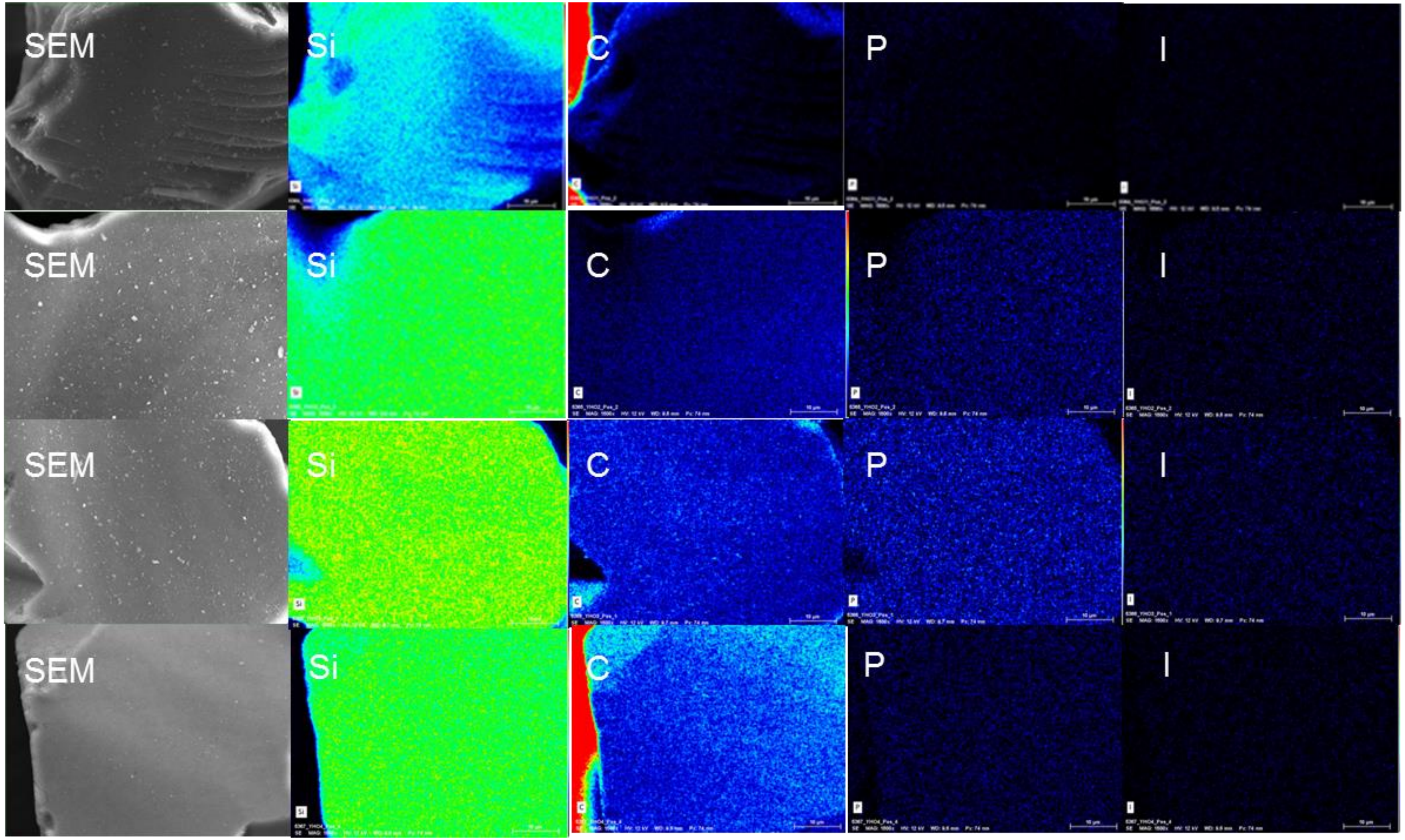

Figure S12 SEM pictures (left column) and EDX mapping of silicon, phosphorus, iodine and carbon for SiO_2 support (first row), impregnated catalyst **5b**@SiO_2 (second row), plasma immobilized fresh catalyst **5b**ⓅSiO_2 (third row) and catalyst **5b**ⓅSiO_2 after 5 runs (forth row).

5. Synthesis of cyclic carbonates 2

General procedure (GP) for the synthesis of various cyclic carbonates 2 using 5bb®SiO₂: A 45 cm^3 stainless-steel autoclave was charged with catalyst **5bb**®SiO_2 (500 mg, 1.0 mol%) and epoxide **1** (13.9 mmol, 1.0 equiv). The autoclave was purged with CO_2 and the reactor was heated to 45 °C for 6 h, while p(CO_2, 45 °C) was kept constant at 1.0 MPa. The reactor was cooled with an ice bath below 20 °C and CO_2 was released slowly. The crude mixture was diluted with EtOAc (30 mL) and filtered over SiO_2. Subsequently, all volatiles were removed in vacuo to obtain carbonates **6**.

4-Ethyl-1,3-dioxalan-2-one (2a)[4]

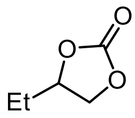

According to the **GP**, 1,2-epoxybutane (**1a**) (1.00 g, 13.9 mmol), and CO_2 were converted to yield **2a** (1.61 g, 13.9 mmol, >99%) as a light yellow oil. ^{1}H NMR (300 MHz, $CDCl_3$, 25 °C): δ = 0.99 (t, J= 7.4 Hz, 3H), 1.70–1.82 (m, 2H), 4.06 (dd, J= 8.4, 7.0 Hz, 1H), 4.51 (dd, J= 8.4, 7.9 Hz, 1H), 4.60–4.67 (m, 1H) ppm.

4-Methyl-1,3-dioxalan-2-one (2b)[4]

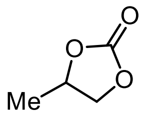

According to the **GP**, 1,2-epoxypropane (**1b**) (810 mg, 13.9 mmol) and CO_2 were converted to yield **2b** (1.41 g, 13.8 mmol, 99%) as a light yellow liquid. ^{1}H NMR (300 MHz, $CDCl_3$, 25 °C): δ = 1.43 (d, J = 6.0 Hz, 3H), 3.95–4.02 (dd, J = 8.4, 7.2 Hz, 1H), 4.49–4.54 (dd, J = 8.4, 7.6 Hz, 1H), 4.81 (m, 1H), ppm.

4-Hexyl-1,3-dioxolan-2-one (2c)[4]

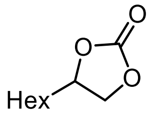

According to the **GP**, 1-(oxiran-2-yl)hexan-1-one (**1c**) (1.98 g, 13.9 mmol), and CO_2 were converted to yield **2c** (2.59 g, 13.9 mmol, >99%) as a yellow oil. ^{1}H NMR (300 MHz, $CDCl_3$, 25 °C): δ = 0.87–0.91 (m, 3H), 1.23–1.52 (m, 8H), 1.60–1.88 (m, 2H), 4.07 (dd, J= 8.4, 7.2 Hz, 1H), 4.52 (dd, J= 8.3, 7.8 Hz, 1H), 4.62–4.79 (m, 1H) ppm.

4-(but-3-en-1-yl)-1,3-dioxolan-2-one (2d)[4]

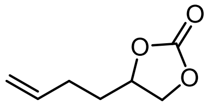

According to the **GP**, 2-(but-3-en-1-yl)oxirane (**1d**) (1.36 g, 13.9 mmol), and CO_2 were converted to yield **2d** (1.98 g, 13.9 mmol, >99%) as a yellow oil. ^1H NMR (300 MHz, $CDCl_3$, 25 °C): δ = 1.84 – 1.71 (m, 1H), 1.93 (td, *J* = 14.0, 7.9 Hz, 1H), 2.32–2.11 (m, 2H), 4.14–4.03 (m, 1H), 4.59–4.48 (m, 1H), 4.80–4.66 (m, 1H), 5.14–5.02 (m, 2H), 5.86–5.69 (m, 1H) ppm.

4-Phenyl-1,3-dioxalan-2-one (2e)[4]

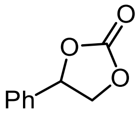

According to the **GP**, 2-phenyloxirane (**1e**) (1.67 g, 13.9 mmol), and CO_2 were converted in the desired carbonate. After purification *via* column chromatography (SiO_2, *c*Hex:EtOAc= 5:1) the product **2e** (2.09 g, 12.7 mmol, 92%) was obtained as a light yellow solid. ^1H NMR (300 MHz, $CDCl_3$, 25 °C): δ = 4.30–4.36 (m, 1H), 4.77–4.82 (dd, *J*= 8.6, 8.2 Hz, 1H), 5.65–5.70 (m, 1H), 7.35–7.38 (m, 2H), 7.43–7.45 (m, 3H) ppm.

4-(Hydroxymethyl)-1,3-dioxalan-2-one (2f)[4]

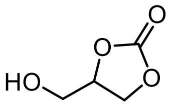

According to the **GP**, 4-(Hydroxymethyl)-1,3-dioxolan-2-one (**1f**) (1.03 g, 13.9 mmol), and CO_2 were converted to yield **2f** (1.40 g, 11.9 mmol, 85%) as a colorless liquid. ^1H NMR (300 MHz, $CDCl_3$, 25 °C): δ = 2.82 (br s, 1H), 3.68–3.74 (dd, *J* = 12.9, 3.5 Hz, 1H), 3.90–4.01 (dd, *J* = 12.9, 3.0 Hz, 1H), 4.44–4.56 (m, 2H), 4.78–4.86 (m, 1H) ppm.

4-(chloromethyl)-1,3-dioxolan-2-one (2g)[4]

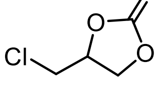

According to the **GP**, 2-(chloromethyl)oxirane (**1g**) (1.29g, 13,9 mmol), and CO_2 were converted to yield **2g** (1.90 g, 13.9 mmol, >99%) as a yellow oil. ^1H NMR (300 MHz, $CDCl_3$, 25 °C): δ = 3.82–3.71 (m, 2H), 4.40–5.02 (m, 1H), 4.42 (dd, *J* = 8.9, 5.7 Hz, 1H), 4.60 (dd, *J* = 8.8, 8.3 Hz, 1H) ppm.

4-(Methoxymethyl)-1,3-dioxalan-2-one (2h)[4]

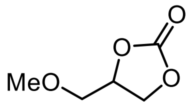

According to the **GP**, 4-(methoxymethyl)-1,3-dioxolan-2-one (**1h**) (1.23 g, 13.9 mmol), and CO_2 were converted to yield **2h** (1.81 g, 13.7 mmol, 99%) as a yellow liquid. ^{1}H NMR (300 MHz, $CDCl_3$, 25 °C): δ = 3.42 (s, 3H), 3.53–3.67 (m, 2H), 4.40–4.33 (m, 1H), 4.49 (t, *J* = 8.3 Hz, 1H), 4.77–4.84 (m, 1H) ppm.

4-(*tert*-Butoxymethyl)-1,2-dioxalan-2-one (2i)[4]

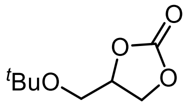

According to the **GP**, 4-(*tert*-butoxymethyl)-1,3-dioxolan-2-one (**1i**) (1.81 g, 13.9 mmol) and CO_2 were converted to yield **2i** (2.32 g, 96%) as a colorless liquid. ^{1}H NMR (300 MHz, $CDCl_3$, 25 °C): δ = 1.17 (s, 9H), 3.49 (m, 1H), 3.55–3.62 (m, 1H), 4.33–4.38 (m, 1H), 4.38–4.48 (m, 1H), 4.77 (m, 1H) ppm.

4-((Allyloxy)methyl)-1,3-dioxolan-2-one (2j)[4]

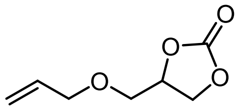

According to the **GP**, 2-((allyloxy)methyl)oxirane (**1j**, 1.59 g, 13.9 mmol), and CO_2 were converted to yield **2j** (1.89 g, 12.0 mmol, 86%) as a yellow liquid. ^{1}H NMR (300 MHz, $CDCl_3$, 25 °C): δ = 3.58–3.73 (m, 2H), 4.02–4.08 (m, 2H), 4.40 (dd, *J*= 8.4, 6.1 Hz, 1H), 4.50 (dd, *J* = 8.3 Hz, 8.3 Hz, 1H), 4.77–4.89 (m, 1H), 5.18–5.33 (m, 2H), 5.79–5.94 (m, 1H) ppm.

4-((2,2,3,3-Tetraflouropropoxy)methyl-1,3-dioxalan-2-one (2k)[4]

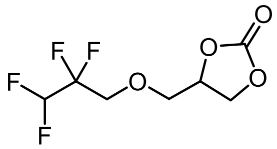

According to the **GP**, 2-((2,2,3,3-tetrafluoropropoxy)methyl)oxirane (**1k**) (2.61 g, 13.9 mmol), and CO_2 were converted to yield **2k** (2.88 g, 12.4 mmol, 89%) as a colorless liquid. ^{1}H NMR (300 MHz, $CDCl_3$, 25 °C): δ = 3.76–4.04 (m, 4H), 4.39 (dd, *J*= 8.5, 6.0 Hz, 1H), 4.54 (t, *J*= 8.5 Hz, 1H), 4.81–4.90 (m, 1H), 5.88 (m, 1H) ppm.

2-(Oxo-1,3-dioxalan-4-yl)methyl methacrylate (2l)[4]

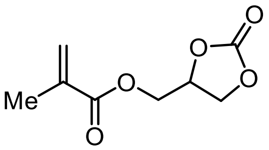

According to the **GP**, oxiran-2-ylmethyl methacrylate (**1l**) (1.98 g, 13.9 mmol), and CO_2 were converted to yield **2l** (2.46 g, 13.2 mmol, 95%) as a dark yellow oil. ^{1}H NMR (300 MHz, $CDCl_3$, 25 °C): δ = 1.95 (s, 3H), 4.30–4.49 (m, 3H), 4.59 (t, *J*= 8.6 Hz, 1H), 4.93–5.02 (m, 1H), 5.66 (s, 1H), 6.13 (s, 1H) ppm.

4-((3-(Triethoxysilyl)propoxy)methyl)-1,3-dioxolan-2-one (2m)

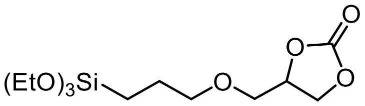

According to the **GP**, (3-glycidoxypropyl)triethoxysilane (**1m**) (3.87 g, 13.9 mmol), and CO_2 were converted to yield **2m** (4.25 g, 13.2 mmol, 95%) as colorless liquid. ^{1}H NMR (300 MHz, $CDCl_3$, 25 °C): δ = 0.57–0.63 (m, 2H), 1.21 (t, *J*= 7.0 Hz, 9H), 1.61–1.71 (m, 2H), 3.49 (m, 2H), 3.61 (dd, *J*= 10.9, 3.8 Hz, 1H), 3.68 (dd, *J*= 10.9, 4.2 Hz, 1H), 3.82 (q, *J*= 7.0 Hz, 6H), 4.40 (dd, *J*= 8.3, 6.2 Hz, 1H), 4.49 (dd, *J*= 8.3 Hz, 1H), 4.75–4.85 (m, 1H) ppm. ^{13}C NMR (75 MHz, $CDCl_3$, 25 °C) δ= 6.4, 18.3, 22.9, 58.4, 66.4, 69.6, 74.1, 75.0, 77.3, 154.9 ppm. IR Neat: 1792.6 cm^{-1} (C=O). HRMS (ESI-TOF/MS): *m/z* calcd. $C_{13}H_{26}O_7SiNa$ [M$^+$+Na]: 345.1340; *m/z* found $C_{13}H_{26}O_7SiNa$ [M$^+$+Na]: 345.1342. Elemental analysis calcd. (%) for $C_{13}H_{26}O_7Si$ (322.43 g mol^{-1}): C 48.43, H 8.13; found: C 50.57, H 8.06.

Hexahydrobenzo[d][1,3]dioxol-2-one (2n)[4]

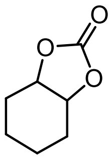

According to the **GP**, 7-oxabicyclo[4.1.0]heptane (**1n**) (1.36 g, 13.9 mmol), and CO_2 were converted to yield **2n** (1.20 g, 8.44 mmol, 61%, *cis*/*trans*> 99:1) as a colorless oil. ^{1}H NMR (300 MHz, $CDCl_3$, 25 °C): δ = 1.35–1.49 (m, 2H), 1.55–1.71 (m, 2H), 1.87–1.93 (m, 4H), 4.58–4.82 (m, 2H) ppm.

Tetrahydrofuro[3,4-d][1,3]dioxol-2-one (2o)[4]

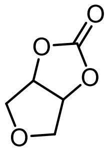

According to the **GP**, 3,6-dioxabicyclo[3.1.0]hexane (**1o**) (1.20 g, 13.9 mmol), and CO_2 were converted to yield **2o** (556 mg, 4.27 mmol, 31%, *cis*/*trans*> 99:1) as a colorless oil. ^{1}H NMR (300 MHz, $CDCl_3$, 25 °C): δ = 3.60–3.53 (m, 2H), 4.26 (d, *J* = 12.4 Hz, 2H), 5.21 (d, *J* = 3.3 Hz, 2H) ppm.

4,5-Diphenyl-1,3-dioxalan-2-one (2p)[4]

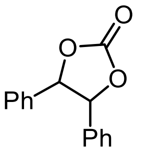

According to the **GP**, *cis* 2,3-diphenyloxiran (**1p**) (0.50 g, 2.55 mmol), 1.0 mL *n*-butanol and CO_2 were converted to yield **2p** (80 mg, 0.33 mmol, 13%, *cis*/*trans*< 1:99) as a yellow solid. ^{1}H NMR (300 MHz, $CDCl_3$, 25 °C): δ= 5.45 (s, 2H), 7.30–7.36 (m, 4H), 7.42–7.48 (m, 6H) ppm.

Methyl 8-(5-octyl-2-oxo-1,3-dioxalan-4-yl)octanoate (2q)[2]

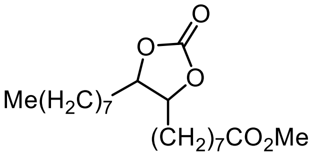

According to the **GP**, epoxidized *cis* methyl oleat (**1q**) (4.34 g, 13.9 mmol), and CO_2 were converted at 90°C for 24 h. After purification *via* column chromatography (SiO_2, *c*Hex:EtOAc= 20:1) the product **2q** (1.49 g, 4.2 mmol, 30%, *cis*/*trans*= 28:72) was obtained as a light yellow oil. *cis*-Isomer: ^{1}H NMR (300 MHz, $CDCl_3$, 25 °C): δ= 0.83–0.94 (m, 3H), 1.16–1.47 (m, 18H), 1.47–1.78 (m, 8H), 2.31 (t, *J*= 7.5 Hz, 2H), 3.67 (s, 3H), 4.58–4.67 (m, 2H) ppm. *trans*-Isomer: ^{1}H NMR (300 MHz, $CDCl_3$, 25 °C) δ= 0.83–0.94 (m, 3H), 1.16–1.47 (m, 18H), 1.47–1.78 (m, 8H), 2.31 (t, *J*= 7.5 Hz, 2H), 3.67 (s, 3H), 4.16–4.28 (m, 2H) ppm.

6. NMR spectra of the synthesized carbonates

^{1}H NMR ($CDCl_3$) of 4-ethyl-1,3-dioxolan-2-one

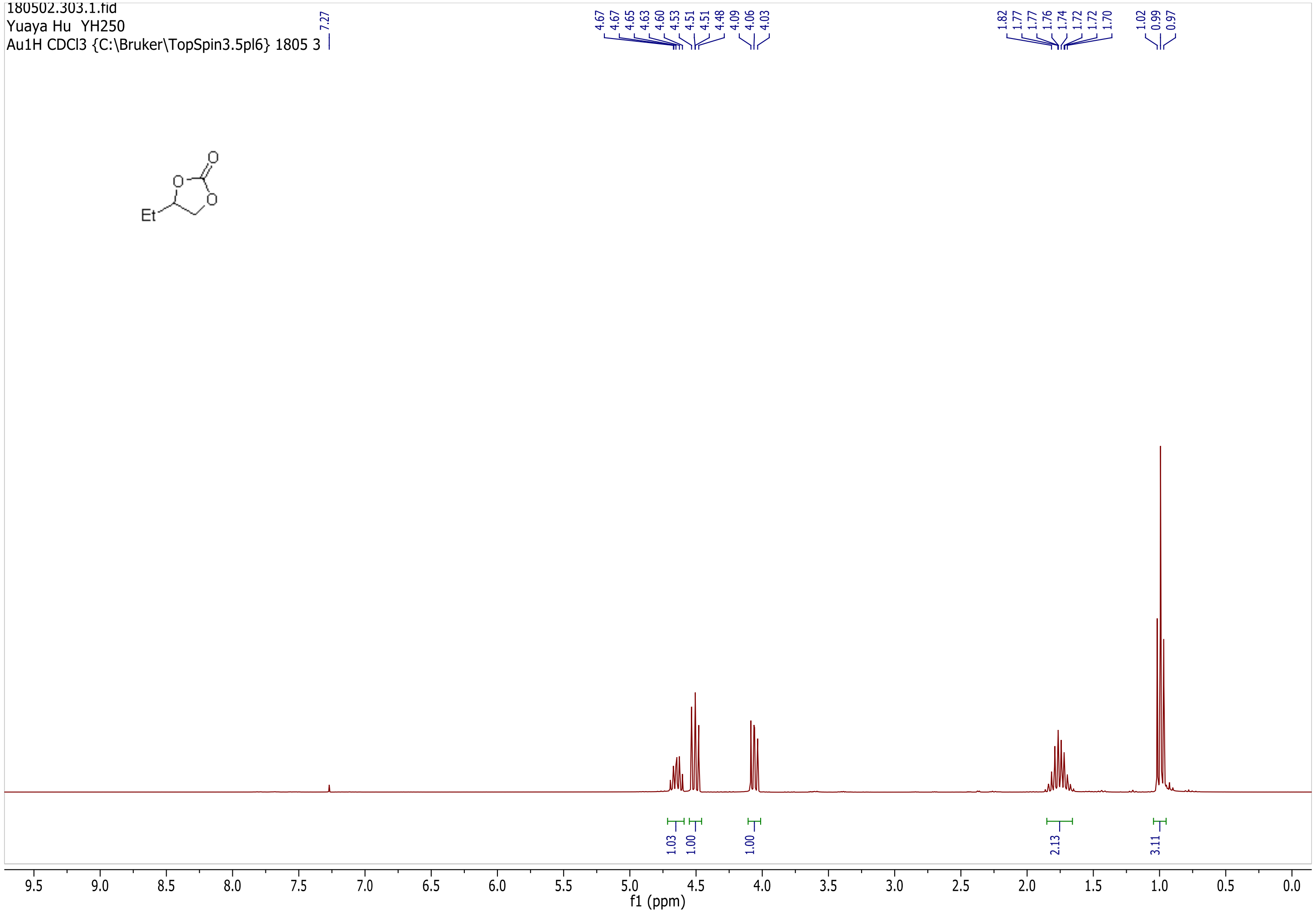

^{1}H NMR ($CDCl_3$) of 4-Methyl-1,3-dioxolan-2-one

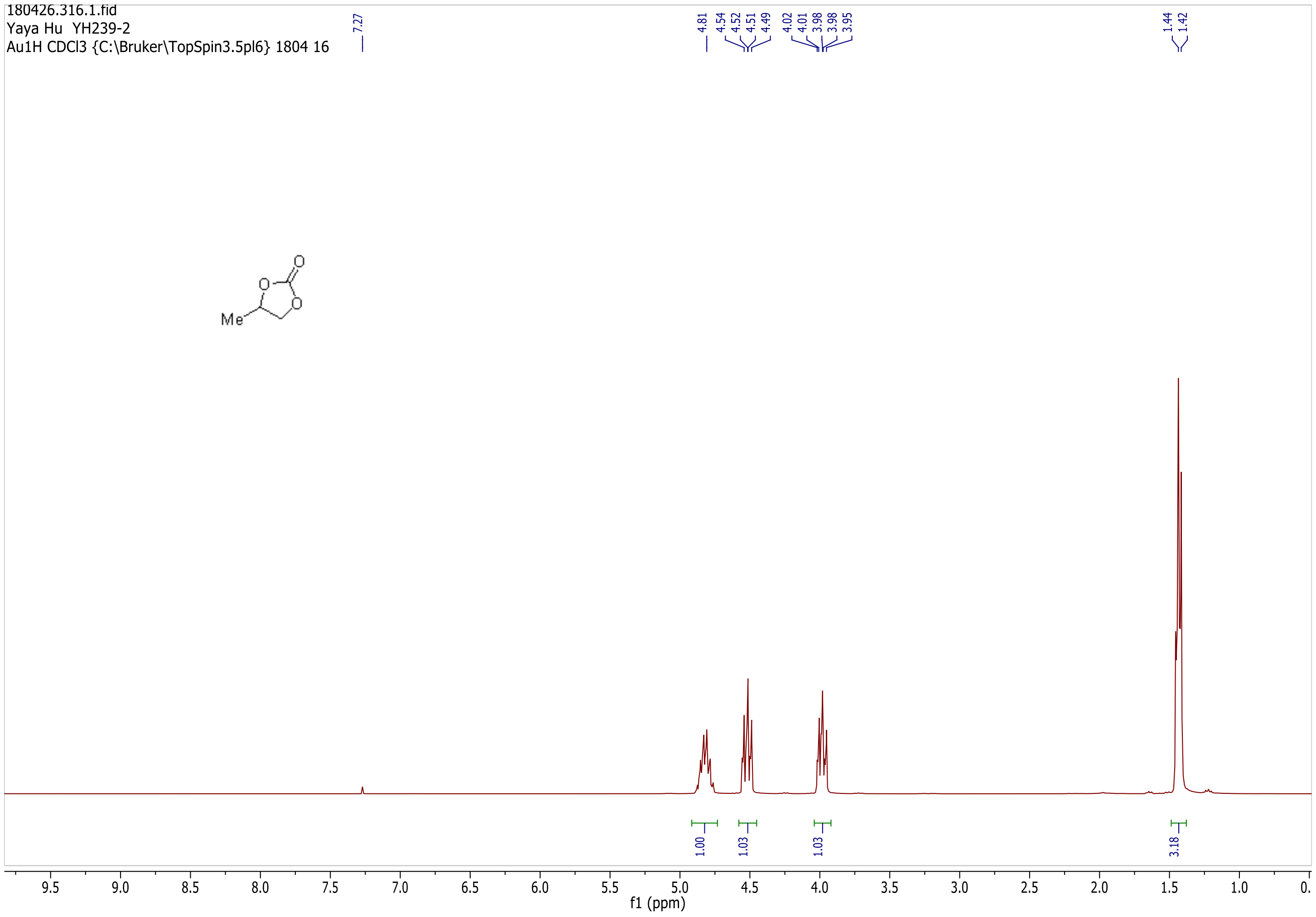

^{1}H NMR ($CDCl_3$) of 4-hexanoyl-1,3-dioxolan-2-one

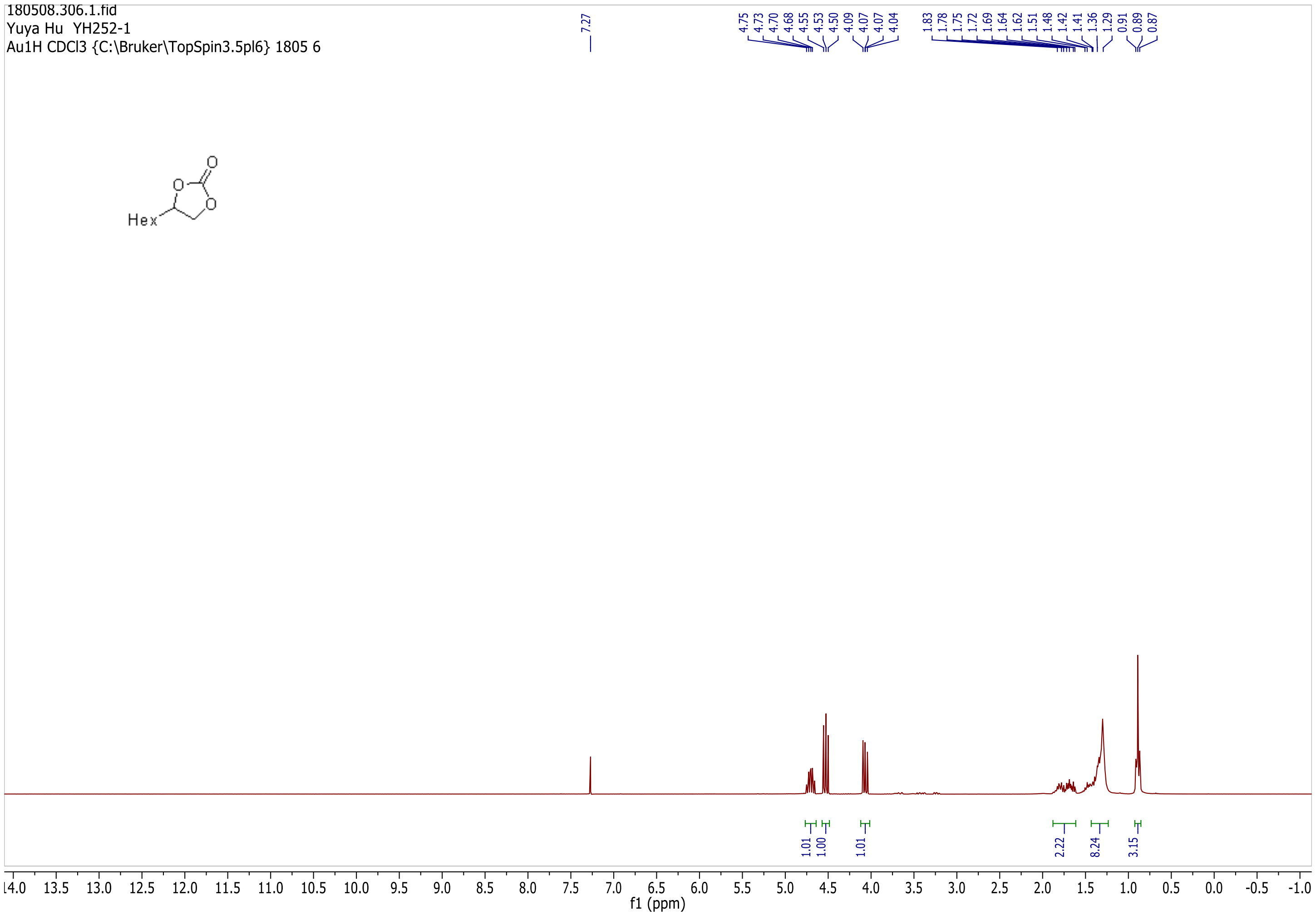

^{1}H NMR ($CDCl_3$) of 4-(but-3-en-1-yl)-1,3-dioxolan-2-one

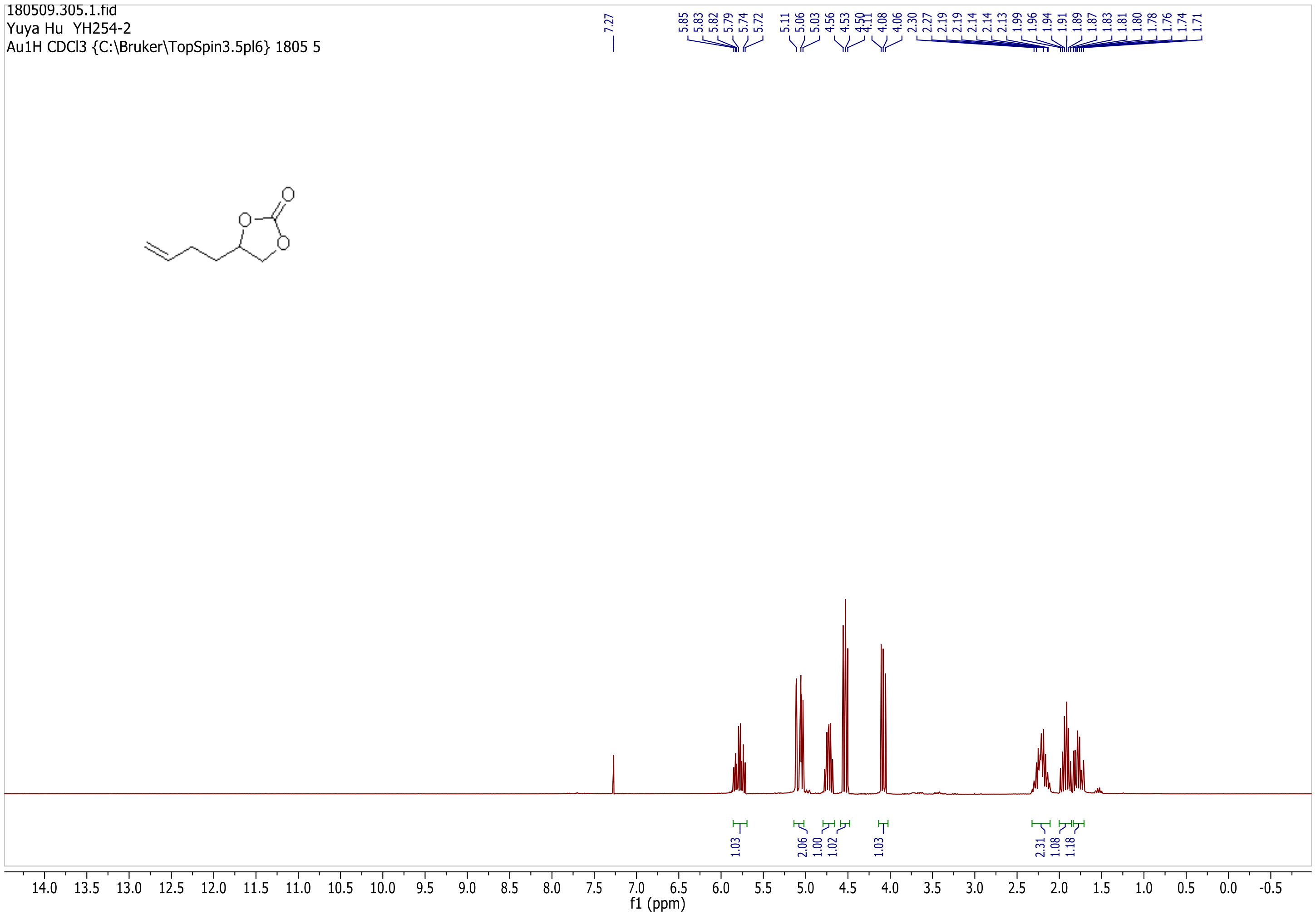

^{1}H NMR ($CDCl_3$) of 4-(chloromethyl)-1,3-dioxolan-2-one

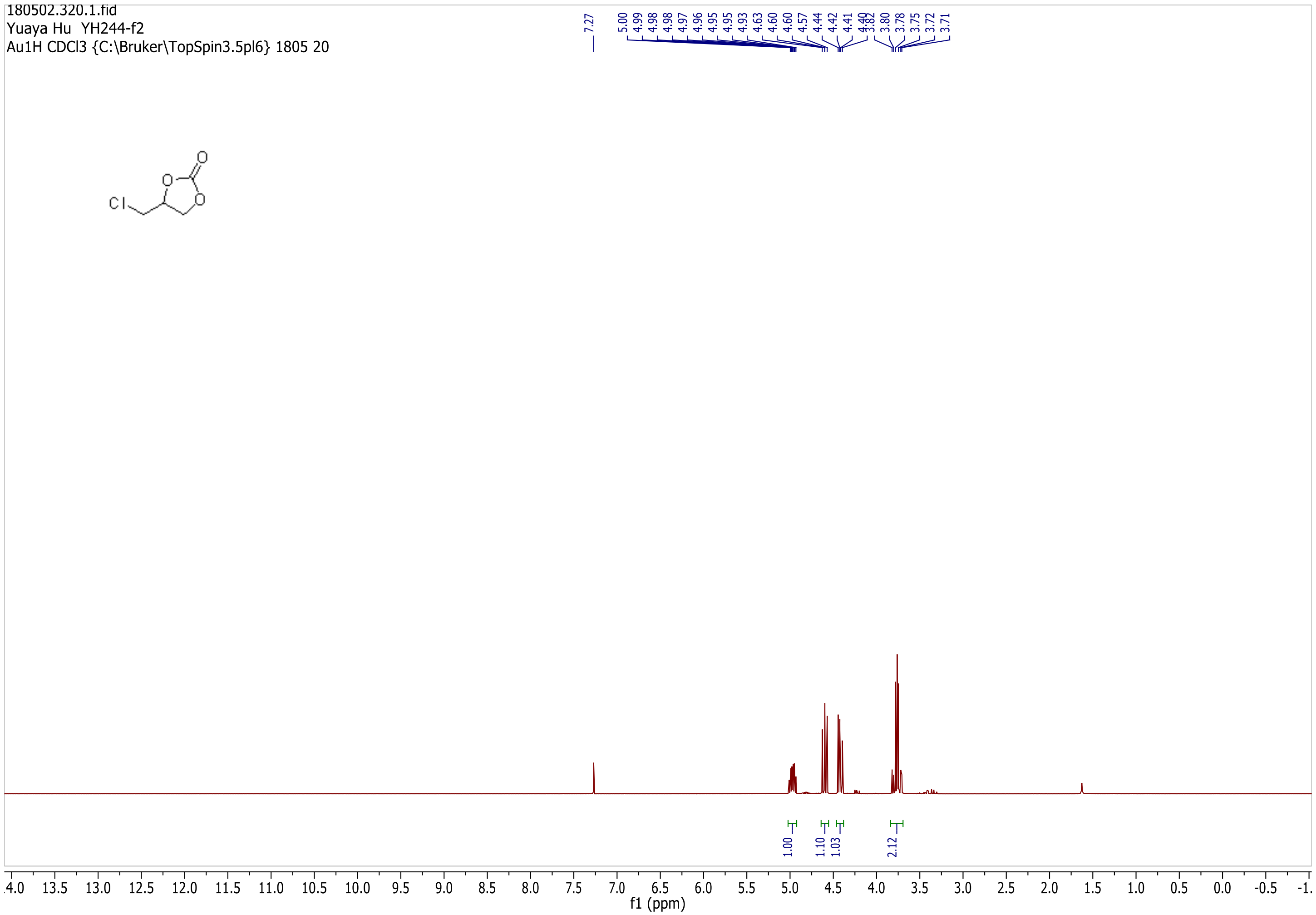

^{1}H NMR ($CDCl_3$) of 4-phenyl-1,3-dioxolan-2-one

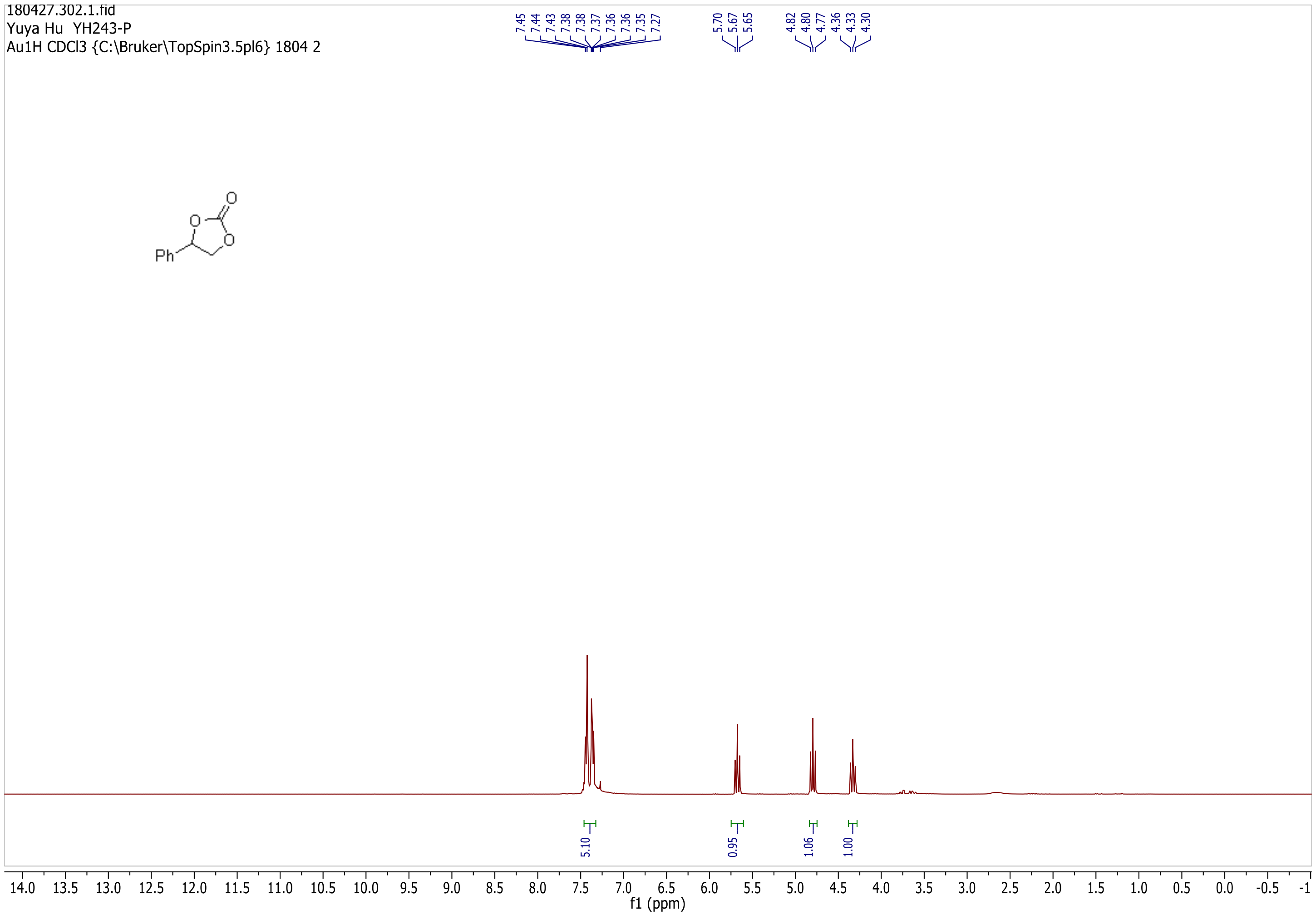

^{1}H NMR ($CDCl_3$) of 4-(hydroxymethyl)-1,3-dioxolan-2-one

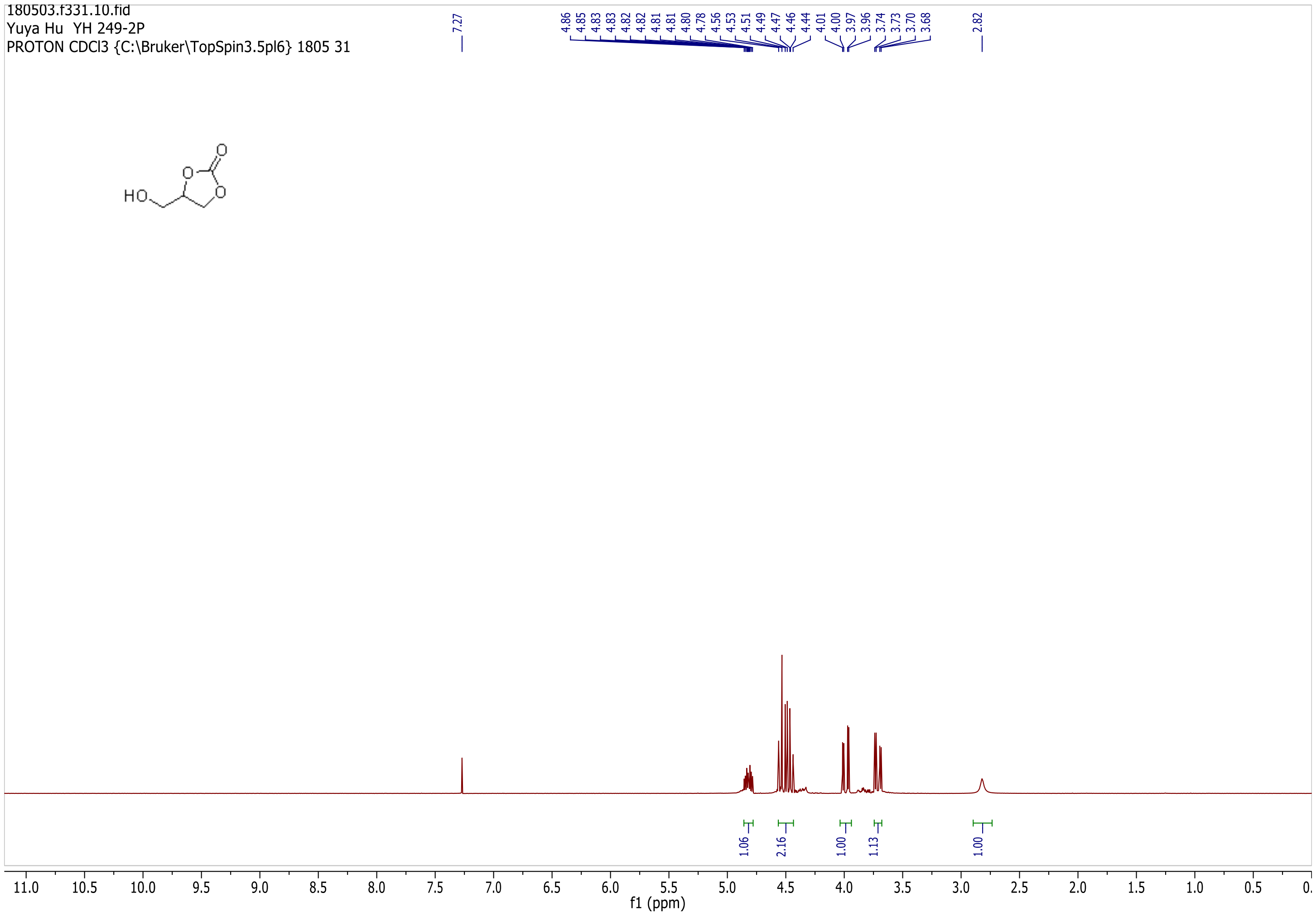

^1H NMR ($CDCl_3$) of 4-(methoxymethyl)-1,3-dioxolan-2-one

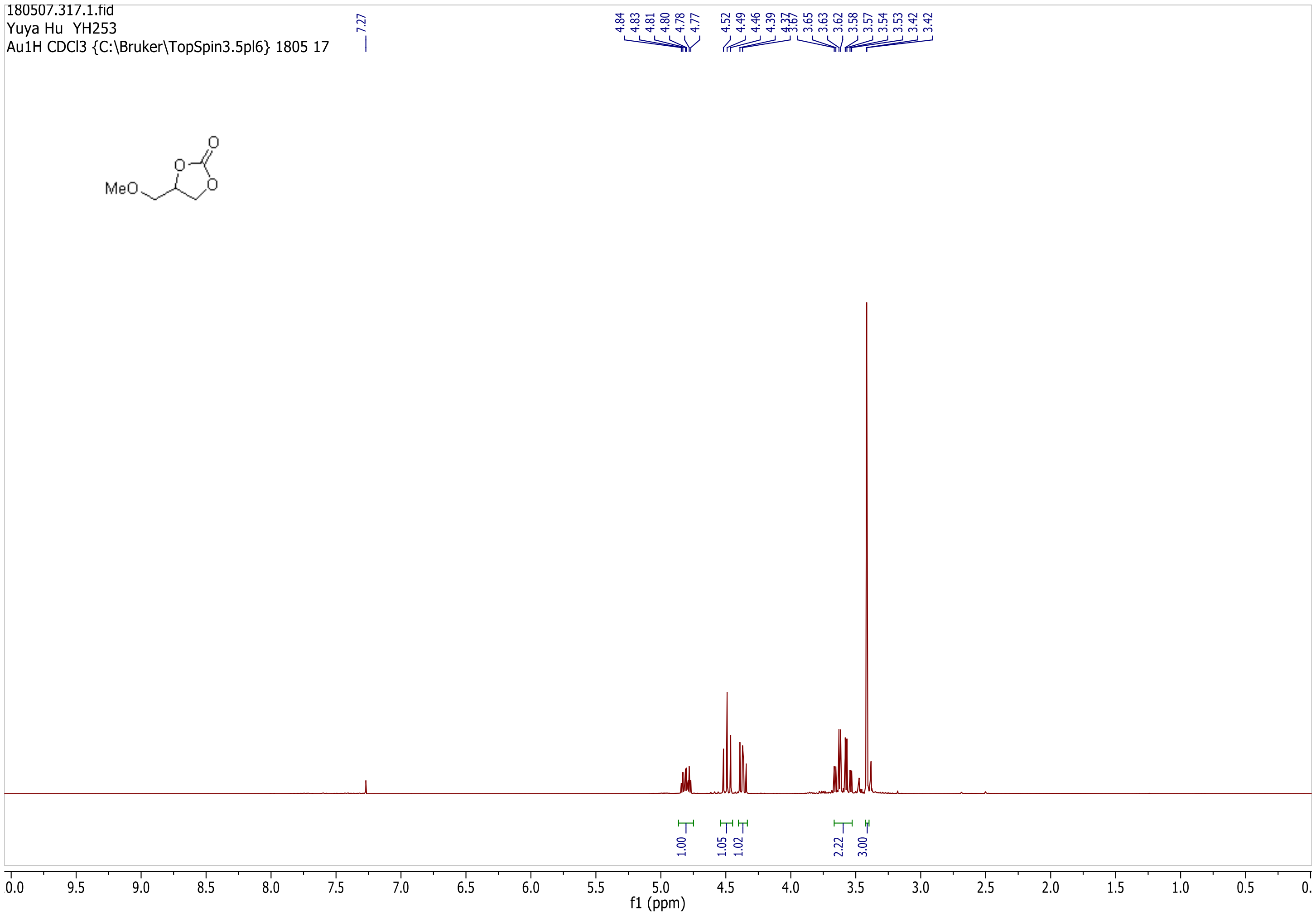

1H NMR ($CDCl_3$) of 4-(*tert*-butoxymethyl)-1,3-dioxolan-2-one

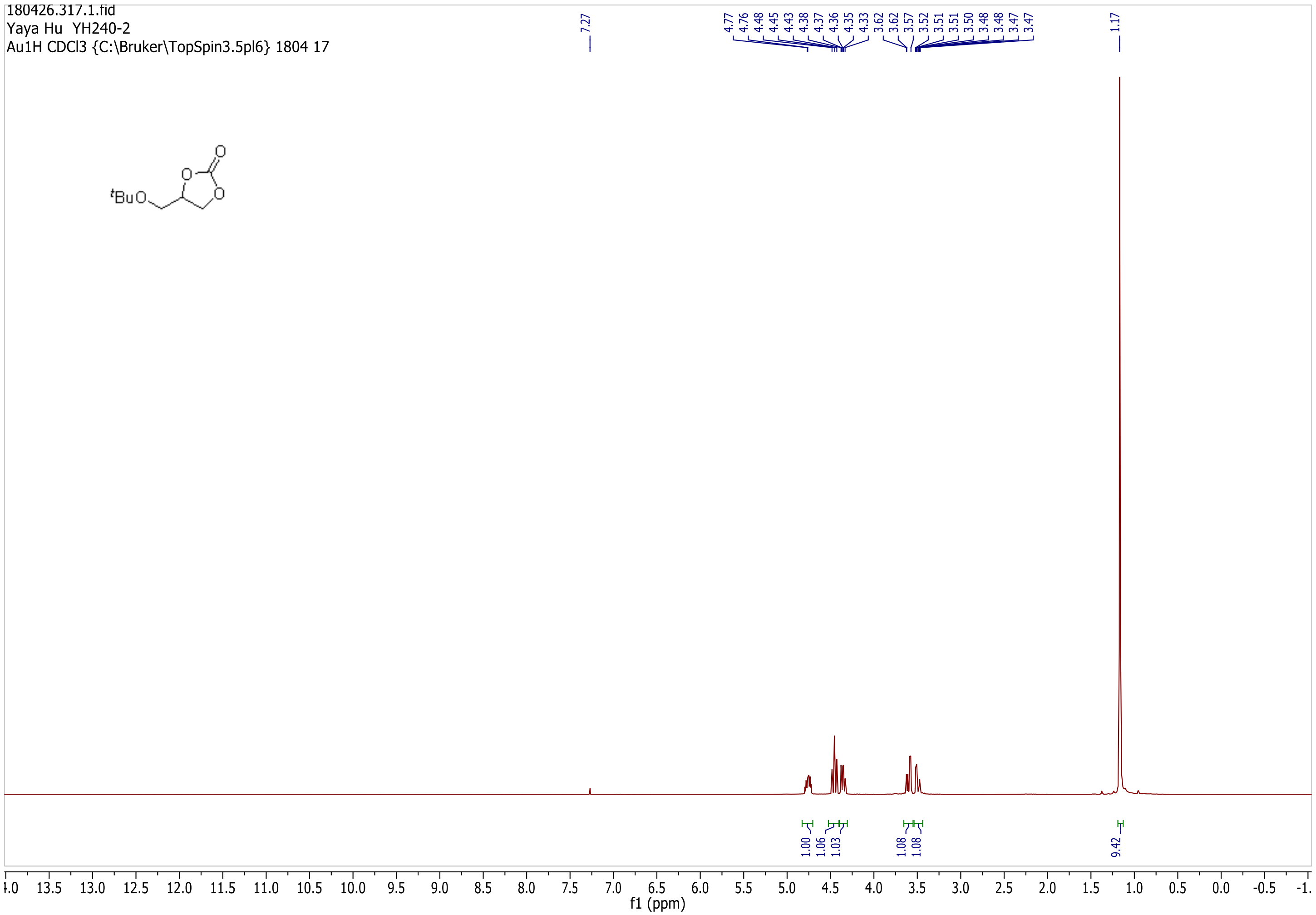

^{1}H NMR ($CDCl_3$) of 4-((allyloxy)methyl)-1,3-dioxolan-2-one

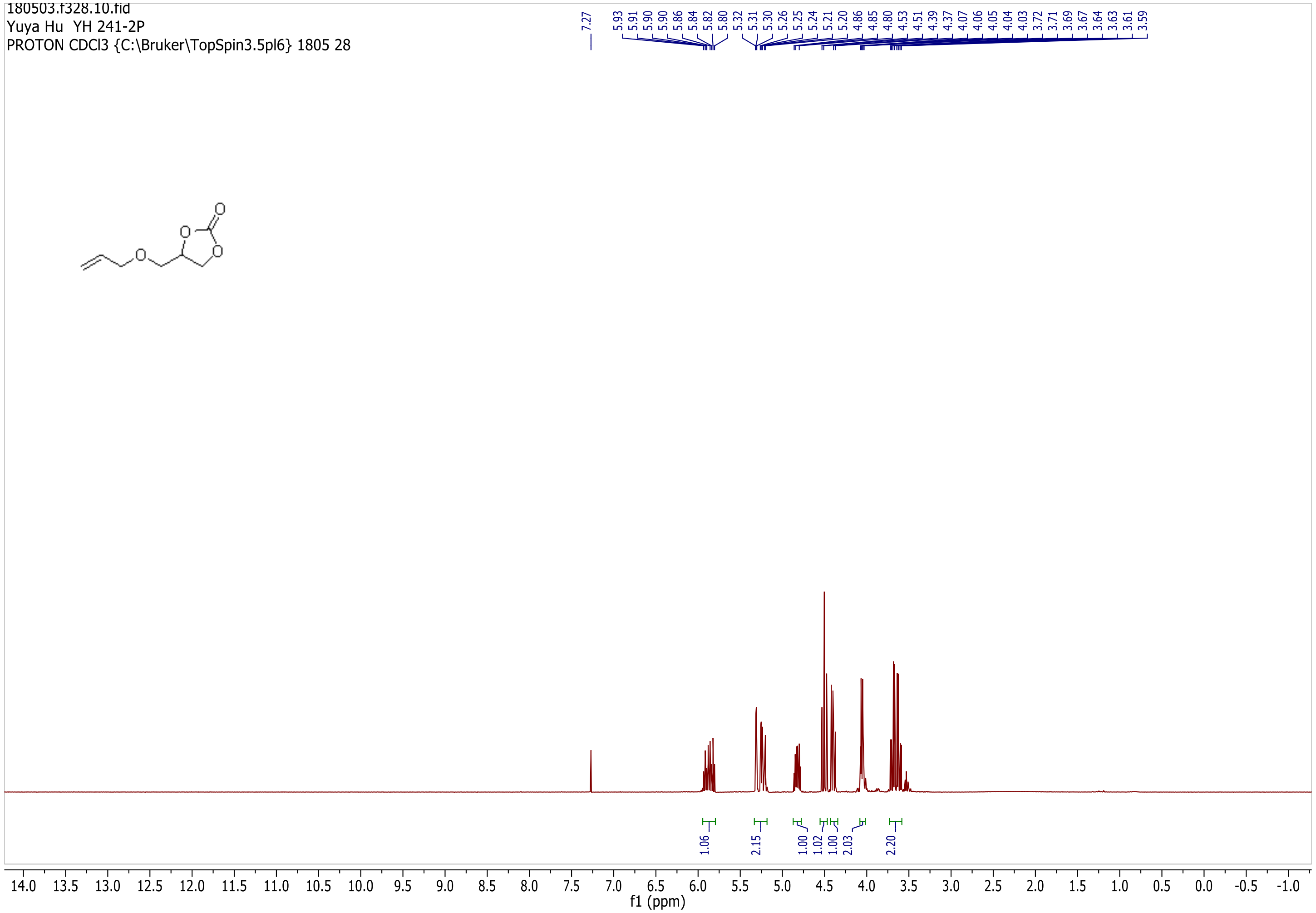

^{1}H NMR ($CDCl_3$) of 4-((2,2,3,3-tetrafluoropropoxy)methyl)-1,3-dioxolan-2-one

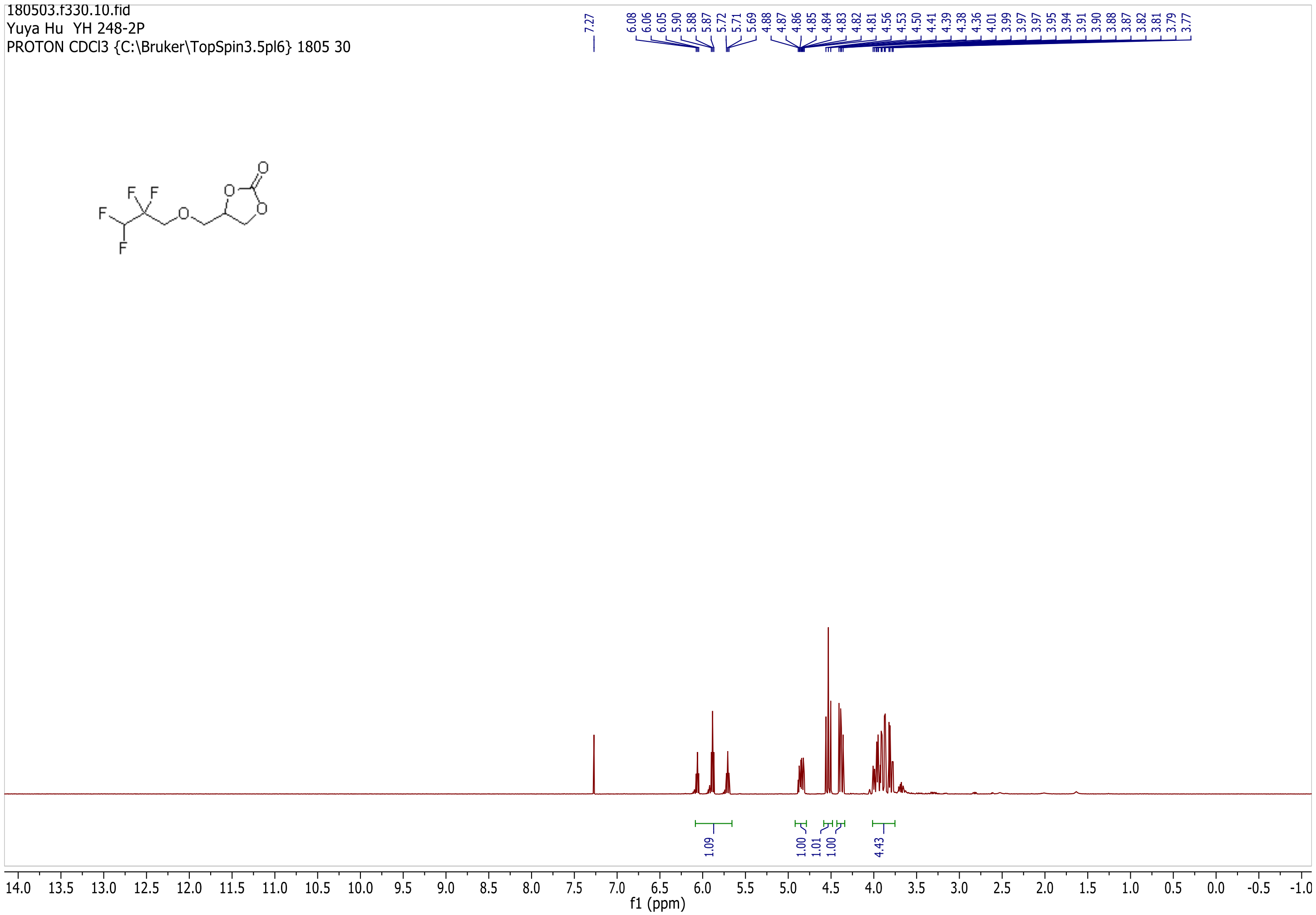

^{1}H NMR ($CDCl_3$) of (2-oxo-1,3-dioxolan-4-yl)methyl 2-methylenebutanoate

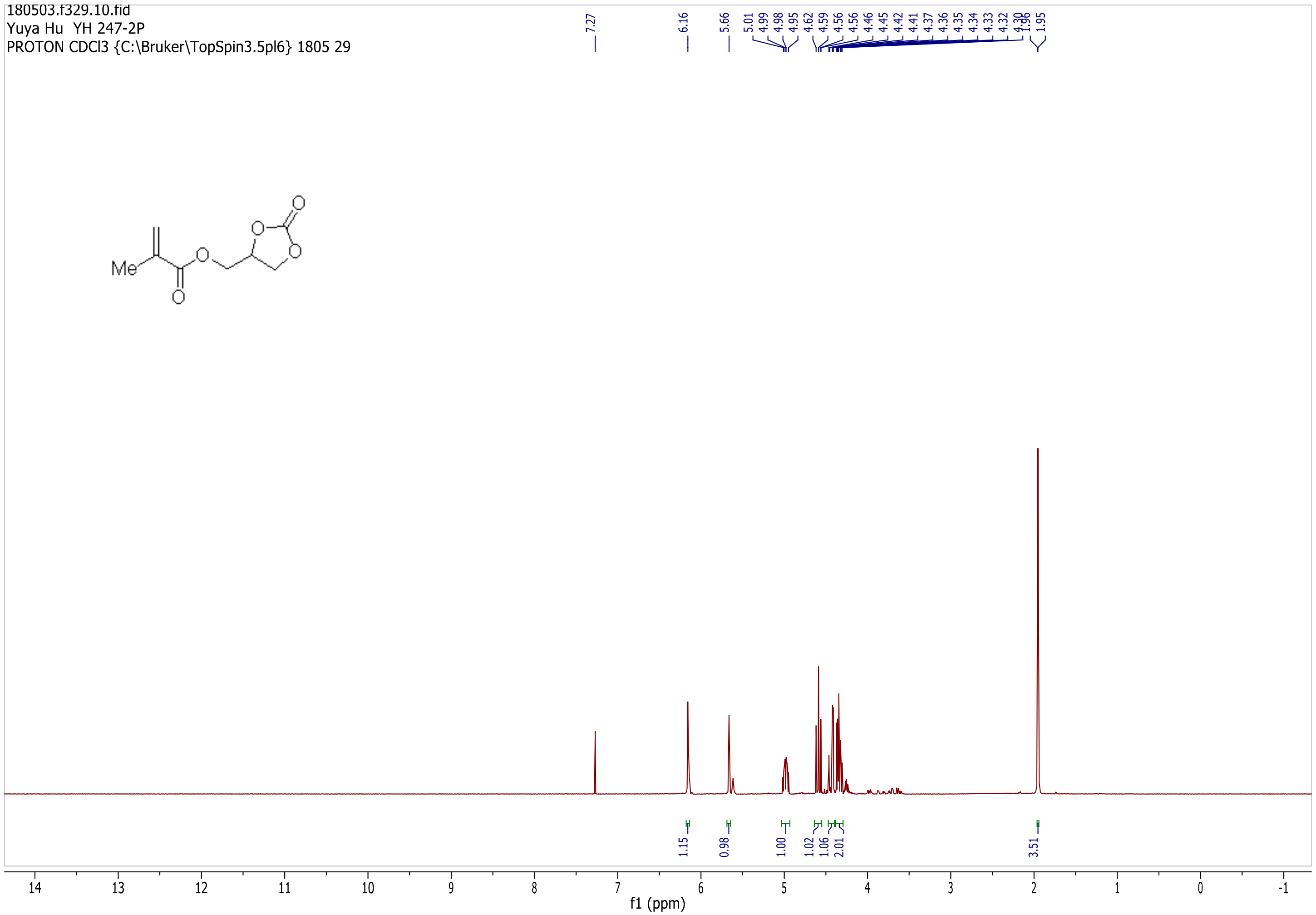

^{1}H NMR ($CDCl_3$) of 4-((3-(triethoxysilyl)propoxy)methyl)-1,3-dioxolan-2-one

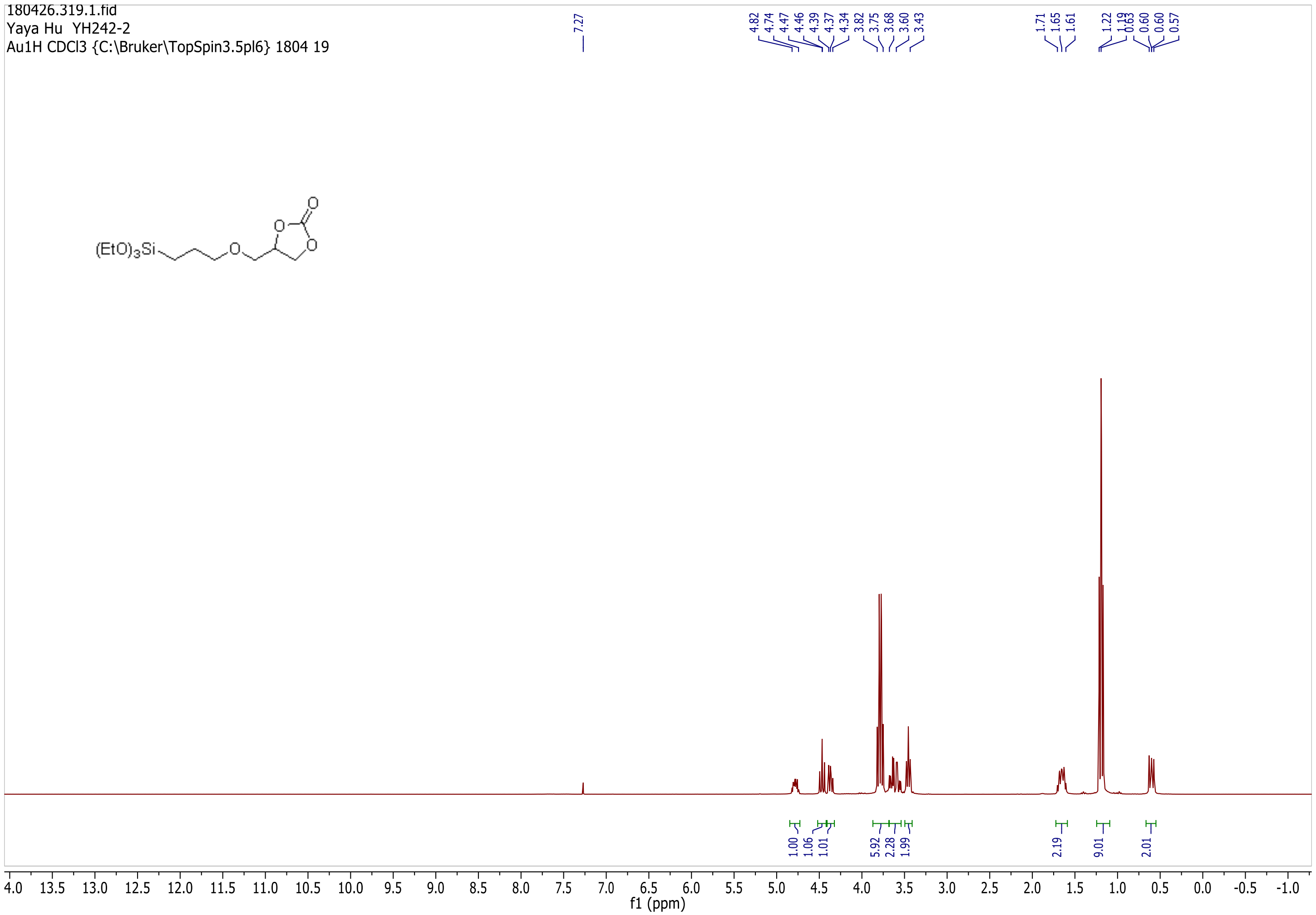

^{1}H NMR ($CDCl_3$) of hexahydrobenzo[d][1,3]dioxol-2-one

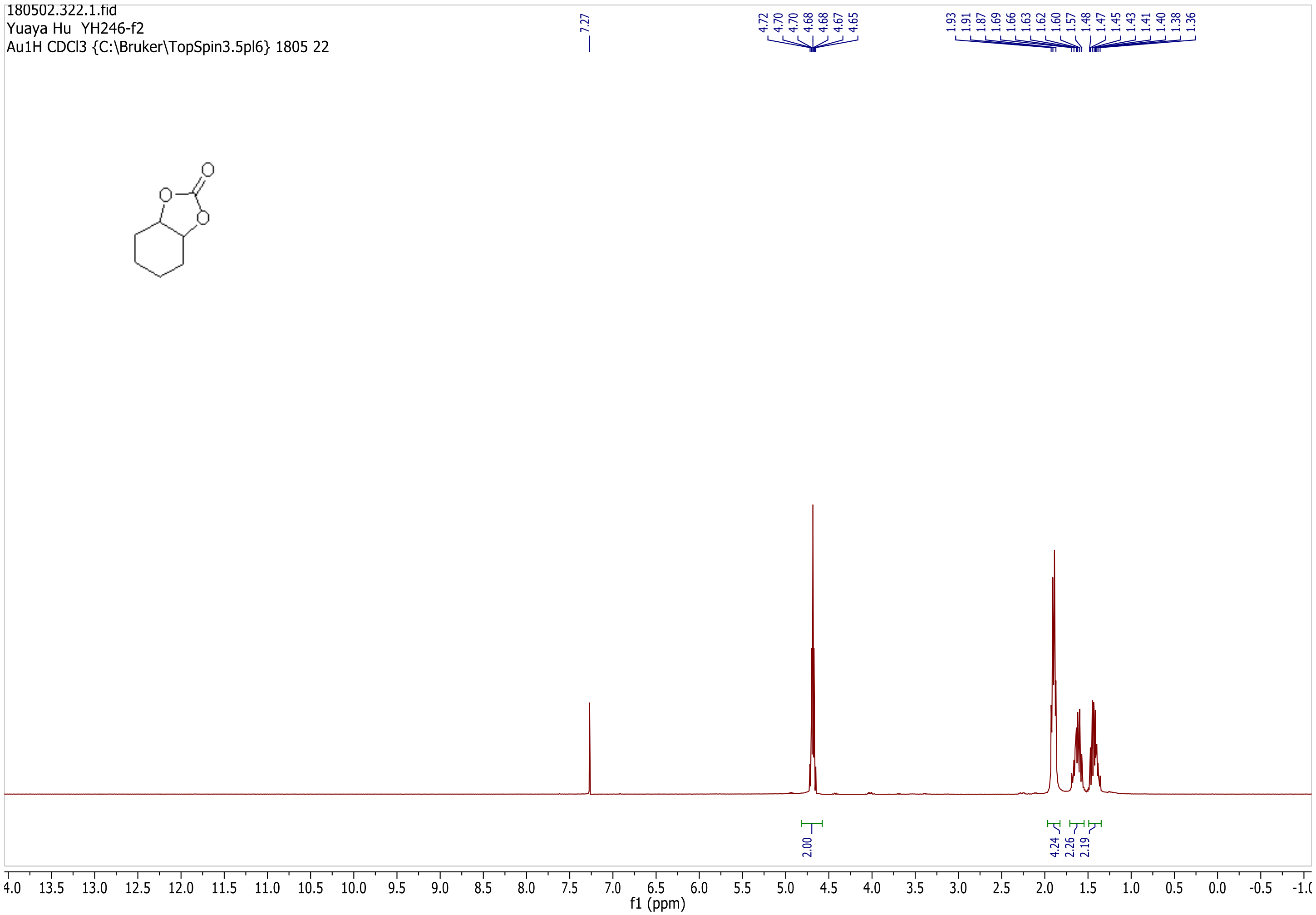

^{1}H NMR ($CDCl_3$) of tetrahydrofuro[3,4-d][1,3]dioxol-2-one

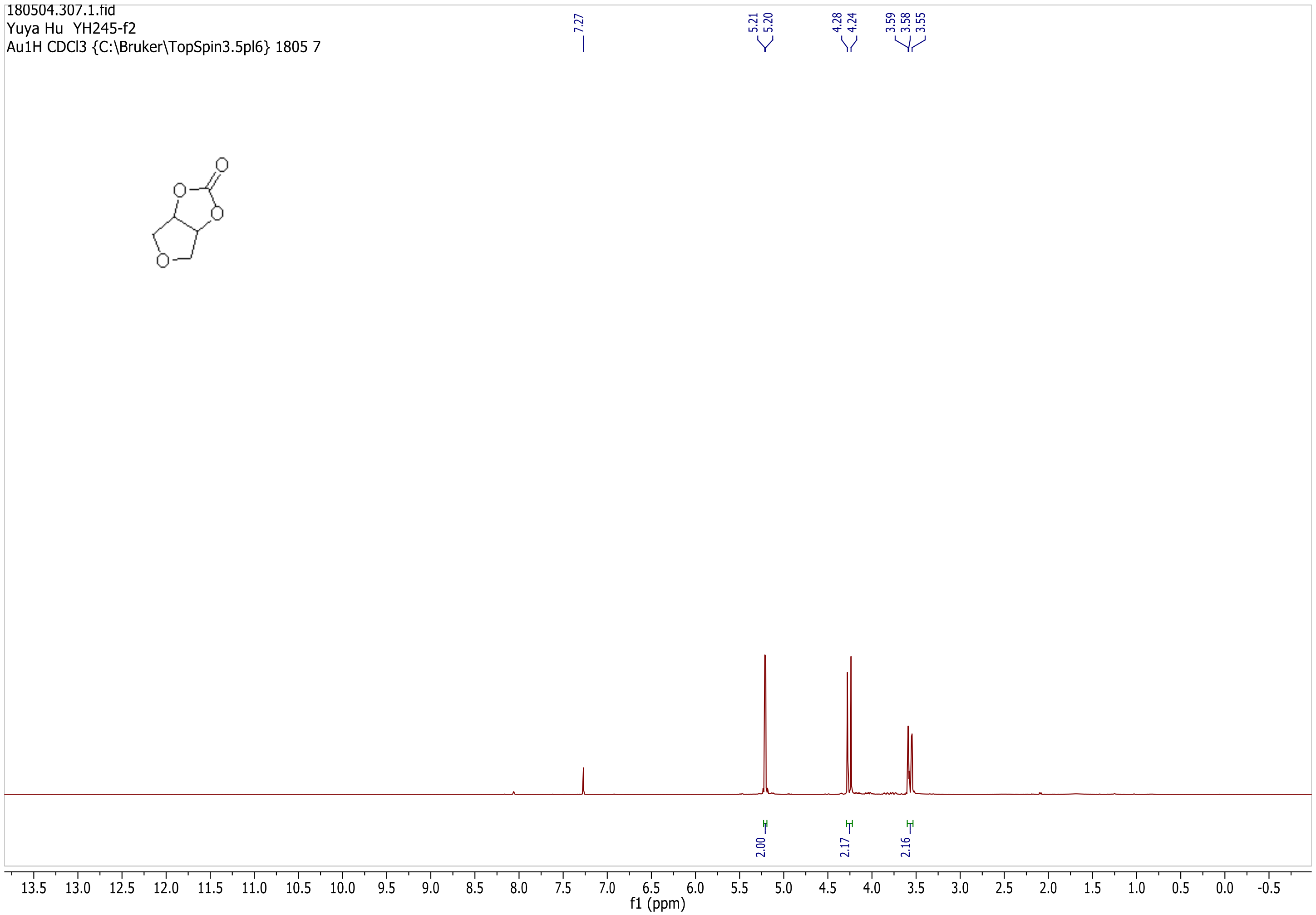

^{1}H NMR ($CDCl_3$) of 4,5-diphenyl-1,3-dioxolan-2-one

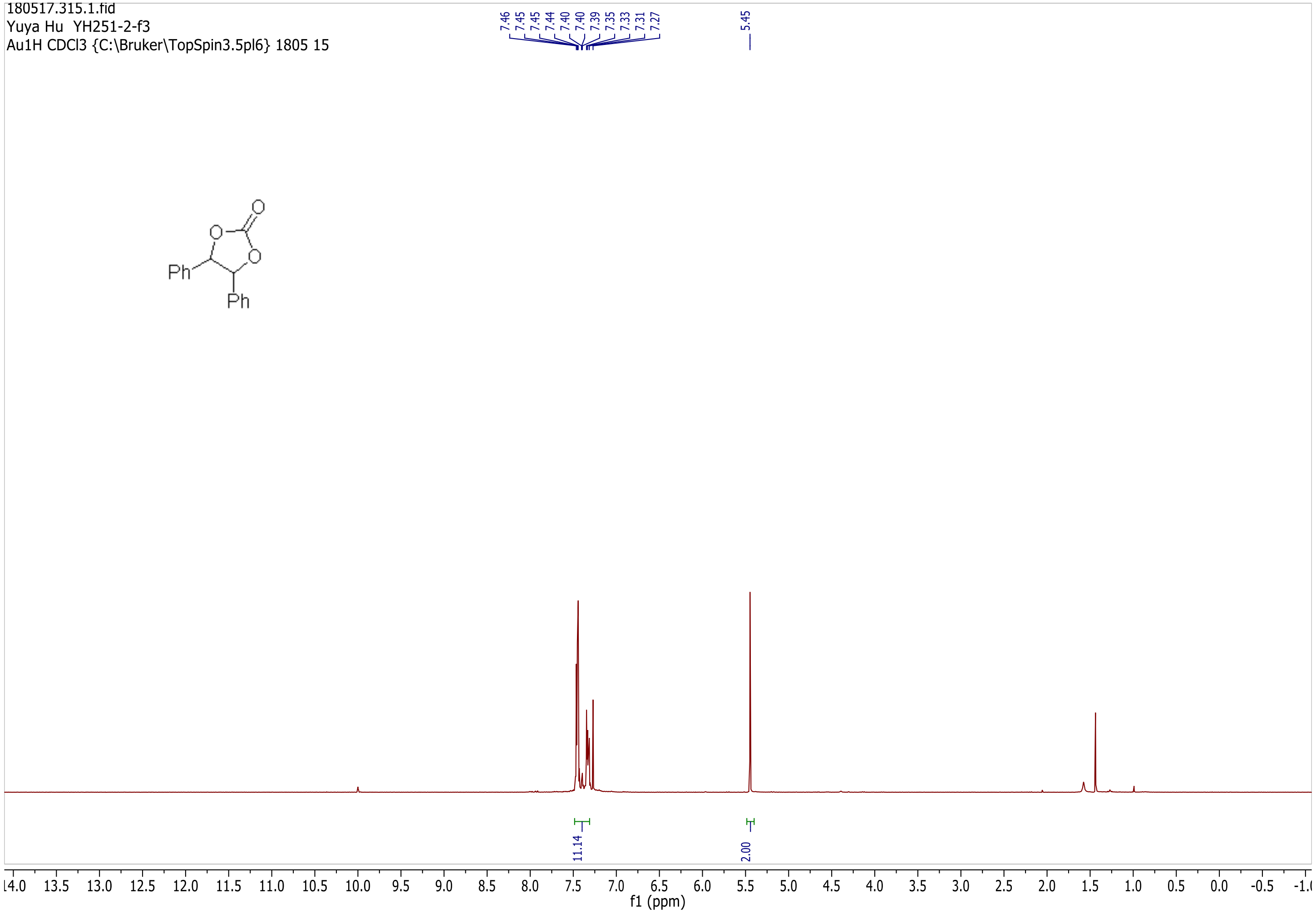

^1H NMR ($CDCl_3$) of methyl 8-(5-octyl-2-oxo-1,3-dioxolan-4-yl)octanoate

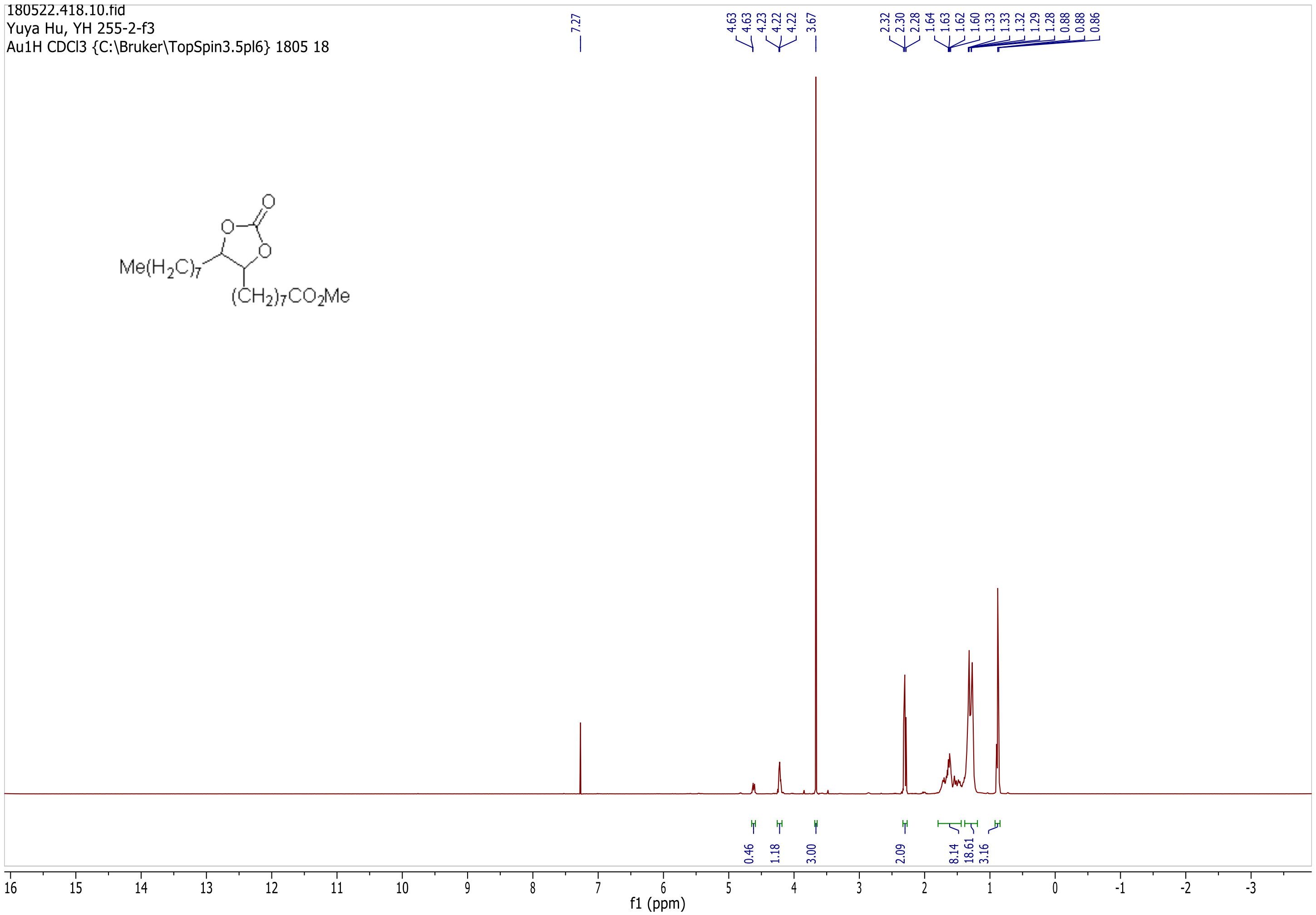

7. References

1 P. B. Kapadnis, E. Hall, M. Ramstedt, W. R. J. D. Galloway, M. Welch and D. R. Spring, *Chem. Comm.* 2009, 538–540.

2 J. Steinbauer, L. Longwitz, M. Frank, J. Epping, U. Kragl and T. Werner, *Green Chem.* 2017, **19**, 4435–4445.

3 H. Büttner, J. Steinbauer, C. Wulf, M. Dindaroglu, H. G. Schmalz and T. Werner, *ChemSusChem*, 2017, **10**, 1076–1079.

4 J. Steinbauer, A. Spannenberg and T. Werner, *Green Chem.* 2017, **19**, 3769–3779.
